# Nivolumab for mismatch-repair-deficient or hypermutated gynecologic cancers: a phase 2 trial with biomarker analyses

**DOI:** 10.1038/s41591-024-02942-7

**Published:** 2024-04-23

**Authors:** Claire F. Friedman, Beryl L. Manning-Geist, Qin Zhou, Tara Soumerai, Aliya Holland, Arnaud Da Cruz Paula, Hunter Green, Melih Arda Ozsoy, Alexia Iasonos, Travis Hollmann, Mario M. Leitao, Jennifer J. Mueller, Vicky Makker, William P. Tew, Roisin E. O’Cearbhaill, Ying L. Liu, Maria M. Rubinstein, Tiffany Troso-Sandoval, Stuart M. Lichtman, Alison Schram, Chrisann Kyi, Rachel N. Grisham, Pamela Causa Andrieu, E. John Wherry, Carol Aghajanian, Britta Weigelt, Martee L. Hensley, Dmitriy Zamarin

**Affiliations:** 1 Gynecologic Medical Oncology Service, Department of Medicine, Memorial Sloan Kettering Cancer Center, New York, NY USA; 2 Department of Medicine, Weill Cornell Medical College, New York, NY USA; 3 Parker Institute for Cancer Immunotherapy, Memorial Sloan Kettering Cancer Center, New York, NY USA; 4 Gynecology Service, Department of Surgery, Memorial Sloan Kettering Cancer Center, New York, NY USA; 5 Department of Epidemiology and Biostatistics, Memorial Sloan Kettering Cancer Center, New York, NY USA; 6 Department of Pathology and Laboratory Medicine, Memorial Sloan Kettering Cancer Center, New York, NY USA; 7 Department of Obstetrics and Gynecology, Weill Cornell Medical College, New York, NY USA; 8 Department of Radiology, Memorial Sloan Kettering Cancer Center, New York, NY USA; 9 Institute of Immunology,University of Pennsylvania, Philadelphia, PA USA; 10 Tisch Cancer Institute,Icahn School of Medicine at Mount Sinai, New York, NY USA

**Keywords:** Medical research, Cancer immunotherapy

## Abstract

Programmed death-1 (PD-1) inhibitors are approved for therapy of gynecologic cancers with DNA mismatch repair deficiency (dMMR), although predictors of response remain elusive. We conducted a single-arm phase 2 study of nivolumab in 35 patients with dMMR uterine or ovarian cancers. Co-primary endpoints included objective response rate (ORR) and progression-free survival at 24 weeks (PFS24). Secondary endpoints included overall survival (OS), disease control rate (DCR), duration of response (DOR) and safety. Exploratory endpoints included biomarkers and molecular correlates of response. The ORR was 58.8% (97.5% confidence interval (CI): 40.7–100%), and the PFS24 rate was 64.7% (97.5% one-sided CI: 46.5–100%), meeting the pre-specified endpoints. The DCR was 73.5% (95% CI: 55.6–87.1%). At the median follow-up of 42.1 months (range, 8.9–59.8 months), median OS was not reached. One-year OS rate was 79% (95% CI: 60.9–89.4%). Thirty-two patients (91%) had a treatment-related adverse event (TRAE), including arthralgia (*n* = 10, 29%), fatigue (*n* = 10, 29%), pain (*n* = 10, 29%) and pruritis (*n* = 10, 29%); most were grade 1 or grade 2. Ten patients (29%) reported a grade 3 or grade 4 TRAE; no grade 5 events occurred. Exploratory analyses show that the presence of dysfunctional (CD8^+^PD-1^+^) or terminally dysfunctional (CD8^+^PD-1^+^TOX^+^) T cells and their interaction with programmed death ligand-1 (PD-L1)^+^ cells were independently associated with PFS24. PFS24 was associated with presence of *MEGF8* or *SETD1B* somatic mutations. This trial met its co-primary endpoints (ORR and PFS24) early, and our findings highlight several genetic and tumor microenvironment parameters associated with response to PD-1 blockade in dMMR cancers, generating rationale for their validation in larger cohorts.

ClinicalTrials.gov identifier: NCT03241745.

## Abstract (web summary)

In a phase 2 trial, nivolumab achieved a response rate of 58% in patients with mismatch-repair-deficient gynecological cancers, meeting the primary endpoint, and genomic and immunologic features correlated with response.

## Main

Endometrial cancer and ovarian cancer are among the most common and fatal malignancies for women in the United States, with an estimated 13,030 and 13,270 deaths, respectively, in 2023 (ref. ^1^). In patients with advanced or recurrent disease, the survival benefit with single-agent chemotherapy or hormone therapy is modest at best^2–4^. Work from The Cancer Genome Atlas (TCGA) has demonstrated that, within endometrial cancer, there are four distinct molecular subtypes: *POLE* ultramutated, microsatellite instability (MSI) hypermutated, copy number-low and copy number-high^5^. These molecular subtypes are associated with progression-free survival (PFS) and overall survival (OS), with *POLE* ultramutated tumors having the best outcomes and copy number-high tumors having the worst. Approximately 30% to 35% of endometrial cancers are classified as MSI or DNA mismatch repair deficient (dMMR)^5,6^. MSI leads to the accumulation of mismatches, insertions and deletions in repeat sequences of DNA—and, thus, a mutation burden approximately 10-fold greater than microsatellite stable (MSS) tumors. Although most common in endometrioid or clear cell histologies, a subset of ovarian cancers also demonstrates dMMR^7^. Based on findings from five single-arm studies that reported durable responses in approximately 50% of treated patients, pembrolizumab, an anti-programmed death-1 (PD-1) monoclonal antibody, was granted accelerated approval by the US Food and Drug Administration (FDA) as the first tissue-site-agnostic agent in patients with MSI-high (MSI-H) or dMMR cancers that progressed after prior treatment^6,8–10^. Subsequently, dostarlimab was granted accelerated approval for patients with dMMR endometrial cancer who progressed on or after treatment with platinum-based chemotherapy^11^. More recently, the randomized, placebo-controlled, phase 3 RUBY (ref. ^12^) and NRG-GY018 (ref. ^13^) studies demonstrated a PFS benefit with the addition of PD-1 blockade to platinum-based chemotherapy for patients with advanced or recurrent endometrial cancer; there was substantial benefit in the dMMR/MSI population.

Aside from the presence of dMMR, predictors of response to PD-1/programmed death ligand-1 (PD-L1) inhibition remain elusive. MSI can arise from somatic or germline mutations in MMR genes (that is, *MLH1*, *MSH2*, *MSH2* and *PMS2*) or hypermethylation of the *MLH1* promoter. It has been suggested that the etiology of dMMR (genetic versus epigenetic) is associated with distinct biology and may be predictive of response to immunotherapy in endometrial cancer^14,15^, and mutations in *JAK1* and *B2M* are associated with resistance^16^. Several tumor microenvironment (TME) features, such as expression of PD-L1 (ref. ^17^) and presence of CD8^+^ T cells in tumors^18^, were demonstrated to be predictive of response to PD-1/PD-L1 inhibitors in some cancer types; however, their predictive value in dMMR gynecologic cancers is unknown. Emerging evidence in other cancer types indicates that T cell functional states, rather than absolute numbers, may serve as a stronger predictor of response to immune checkpoint blockade^19^. Specifically, upregulation of markers of T cell dysfunction/exhaustion, such as PD-1, is associated with tumor antigen reactivity. The transcription factor TOX was identified as a master regulator driving the molecular and epigenetic programs of T cell dysfunction/exhaustion in tumors^20^. However, how these parameters predict response to PD-1 inhibition in patients with dMMR gynecologic cancers is unknown.

Here we report on a phase 2 study of nivolumab, a fully humanized monoclonal antibody to PD-1, in patients with dMMR/MSI-H advanced or recurrent endometrial or ovarian cancer (ClinicalTrials.gov identifier NCT03241745). We demonstrate that nivolumab is active in patients with dMMR gynecologic cancers, and we identify genomic and TME parameters predictive of response that may help guide patient selection for future trials.

## Results

### Study design

Eligible patients had recurrent endometrial cancer or a carcinosarcoma or an endometrioid or clear cell carcinoma that appeared to have originated in the ovary/fallopian tube or peritoneum and met one of the following criteria: (1) dMMR, as determined by loss of expression assessed by immunohistochemistry of one or more of the MMR proteins (MSH2, MSH6, MLH1 and PMS2)*;* (2) MSI-H, as determined by next-generation sequencing (NGS) using Memorial Sloan Kettering Cancer Center-Integrated Mutation Profiling of Actionable Cancer Targets (MSK-IMPACT; MSIsensor)^21,22^; or (3) hypermutated tumors, defined as 20 or more non-synonymous somatic mutations on MSK-IMPACT. The study protocol underwent two noteworthy amendments. Additional details about these amendments, patient selection and trial design are provided in the Methods section.

**Fig. 1:**
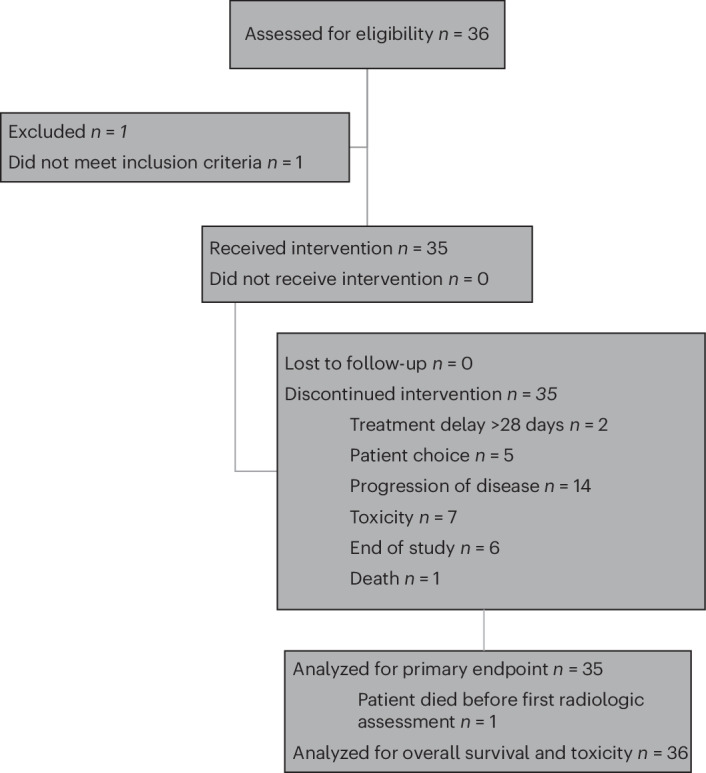
CONSORT (Consolidated Standards of Reporting Trials) diagram showing the flow of patients and their disposition. CONSORT diagram. The trial was terminated as of 1 July 2022, and all remaining patients on therapy were transitioned to standard care.

In total, 35 patients were enrolled in this study; the first patient consented on 27 September 2017 and the final patient on 24 May 2021. (Fig. 1). Patients received nivolumab 240 mg every 2 weeks or 480 mg every 4 weeks until progressive disease or unacceptable toxicity. Baseline patient characteristics are reported in Table 1. The median age was 64 years (range, 36–87 years), and 77% of patients were White, 11% were Black and 6% were Asian. Most patients (83%) had endometrioid endometrial cancer. All enrolled patients were biologically female; gender information was not collected on the study.

**Table 1:** Demographics and baseline characteristics of patients with dMMR, MSI-H or hypermutated endometrial or ovarian cancer

Characteristic	
Age (median, range)	64 (36–87)
Ethnicity (*n*, %)	
Non-Hispanic	31 (89%)
Hispanic	3 (9%)
Ethnicity not known	1 (3%)
Race (*n*, %)	
White	27 (77%)
Black	4 (11%)
Asian	2 (6%)
Other/Unknown	2 (6%)
Stage at diagnosis (*n*, %)	
I	10 (29%)
II	6 (17%)
III	12 (34%)
IV	7 (20%)
Histology (*n*, %)	
Endometrioid FIGO G1	8 (23%)
Endometrioid FIGO G2	8 (23%)
Endometrioid FIGO G3	13 (37%)
Clear cell carcinoma	2 (6%)
Dedifferentiated/Undifferentiated	4 (11%)
Molecular subtype (*n*, %)	
Germline MMR mutation	5 (14%)
*MLH1* promoter hypermethylation	23 (66%)
Other/Unknown^a^	7 (20%)
No. of prior lines of cytotoxic therapy (*n*, %)	
1	30 (86%)
2	4 (11%)
3	1 (3%)

^a^ Other molecular subtypes; see Supplementary Table 1 for more information.

FIGO, International Federation of Gynecology and Obstetrics.

### Primary endpoint results

**Fig. 2:**
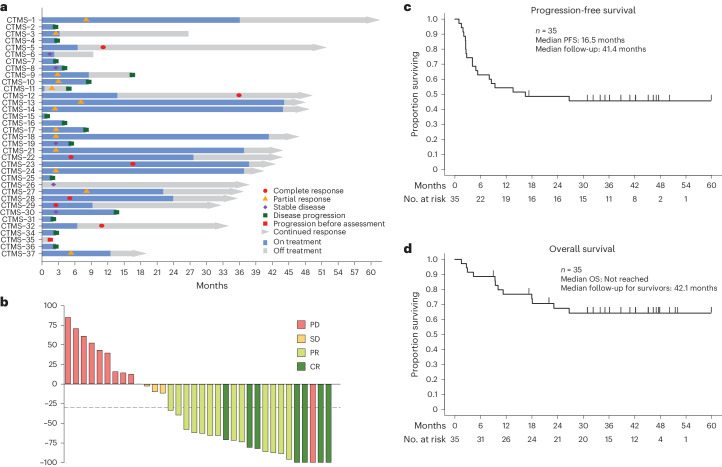
Efficacy outcomes for patients. **a**, Swimmer plot depicting individual duration of treatment, response and clinical outcome at data cutoff (*n* = 35). **b**, Waterfall plot of best percentage tumor change from baseline (*n* = 34) **c**, Kaplan–Meier graphical representation of PFS (*n* = 35). **d**, Kaplan–Meier graphical representation of OS (*n* = 35). CR, complete response; PD, progressive disease; PR, partial response; SD, stable disease.

The co-primary endpoints were to define (1) the objective response rate (ORR) and (2) the PFS at 24 weeks (PFS24). A total of 35 of a planned 40 patients were enrolled and treated. One patient was classified as non-evaluable due to progression of disease and death before the first scheduled follow-up assessment. The trial met its primary endpoint early, with 20 of 34 evaluable patients achieving an objective response by Response Evaluation Criteria in Solid Tumors (RECIST) version 1.1; thus, it was closed early. In the evaluable cohort (*n* = 34), the ORR was 58.8% (97.5% confidence interval (CI): 40.7–100%), with seven complete responses and 13 partial responses (Fig. 2a,b and Extended Data Table 1). Objective responses were noted in both ovarian and endometrial cancers across histologic subtypes and grades and mechanisms of dMMR (*MLH1* hypermethylation, somatic mutation or germline mutation in an MMR gene) (Extended Data Table 2). Of note, one patient was enrolled based on partial MLH1 loss by immunohistochemistry but was found to have MSS disease and low tumor mutational burden (TMB) on MSK-IMPACT testing, suggesting that she actually had MMR-proficient disease. This patient had progression of disease at her first follow-up scan and came off study. PFS24 was a co-primary endpoint; in 34 evaluable patients, the PFS24 rate was 64.7% (97.5% one-sided CI: 46.5–100%), meeting the pre-specified endpoint of 50%.

### Secondary endpoint results

Secondary endpoints included PFS, OS, duration of response (DOR), disease control rate (DCR) and safety. The DCR was 73.5% (95% CI: 55.6–87.1%) (Extended Data Table 1). Among the responders, with a median follow-up of 28 months (range, 2.5–42.5 months), the median DOR was not reached (Fig. 2a). At the time of data cutoff, with a median follow-up of 42.1 months (range, 8.9–59.8 months), the median PFS was 21.6 months (95% CI: 4.9–not evaluable (NE)), and the median OS was not reached; the 1-year OS rate was 79% (95% CI: 60.9–89.4%) (Fig. 2c,d).

All 35 patients were evaluated for adverse events (AEs) (Table 2 and Extended Data Table 3). Thirty-two patients (91%) had a treatment-related adverse event (TRAE) of any grade (Extended Data Tables 4 and 5). Among all 35 patients, the most common TRAEs were arthralgia (*n* = 10, 29%), fatigue (*n* = 10, 29%), pain (*n* = 10, 29%) and pruritis (*n* = 10, 29%) (Table 2). Ten patients (29%) reported a grade 3 or grade 4 TRAE, including immune-mediated toxicities such as myocarditis (*n* = 1, 3%), optic neuritis (*n* = 1, 3%), hemolysis (*n* = 1, 3%) and type 1 diabetes (*n* = 1, 3%) (Extended Data Table 4); there were no grade 5 events. The patient who developed insulin-dependent diabetes remained on study after her blood sugars were stabilized. The patients who developed non-endocrine grade 3/4 events discontinued nivolumab therapy and were treated with steroids and other steroid-sparing immunosuppressive agents per published guidelines. In all patients, the events resolved with appropriate medical management.

**Table 2:** TRAEs

Toxicity (selected TRAEs^a^)	Grade 1/2, *n*(%)	Grade 3/4, *n* (%)	Any grade, *n* (%)
Arthralgia	9 (26)	1 (3)	10 (29)
Diarrhea	7 (20)	0 (0)	7 (20)
Dyspnea	7 (20)	1 (3)	8 (23)
Fatigue	10 (29)	0 (0)	10 (29)
Gastrointestinal disorders	7 (20)	0 (0)	7 (20)
Myalgia	4 (11)	0 (0)	4 (11)
Nausea/vomiting	8 (23)	0 (0)	8 (23)
Nervous system disorders	5 (14)	0 (0)	5 (14)
Pain	9 (26)	1 (3)	10 (29)
Pruritis	10 (29)	0 (0)	10 (29)
Rash	6 (17)	1 (3)	7 (20)
Skin disorders	5 (14)	0 (0)	5 (14)

^a^Shown are TRAEs of any grade that occurred in more than 10% of patients.

Grade 3 myocarditis was diagnosed in one patient who presented for evaluation for fatigue and double vision 2 weeks after the first dose of nivolumab. An electrocardiogram (EKG) was performed, which recorded complete atrioventricular block with ventricular escape rhythm. She was admitted to an outside cardiac intensive care unit where she was treated with prednisone, mycophenolate mofetil and beta blocker; she did not require a permanent pacemaker. She had a concurrent diagnosis of grade 2 myasthenia gravis. Her EKG normalized, and her myasthenia gravis symptoms resolved, and she was able to be tapered off all immunosuppression.

### TME analyses

**Fig. 3:**
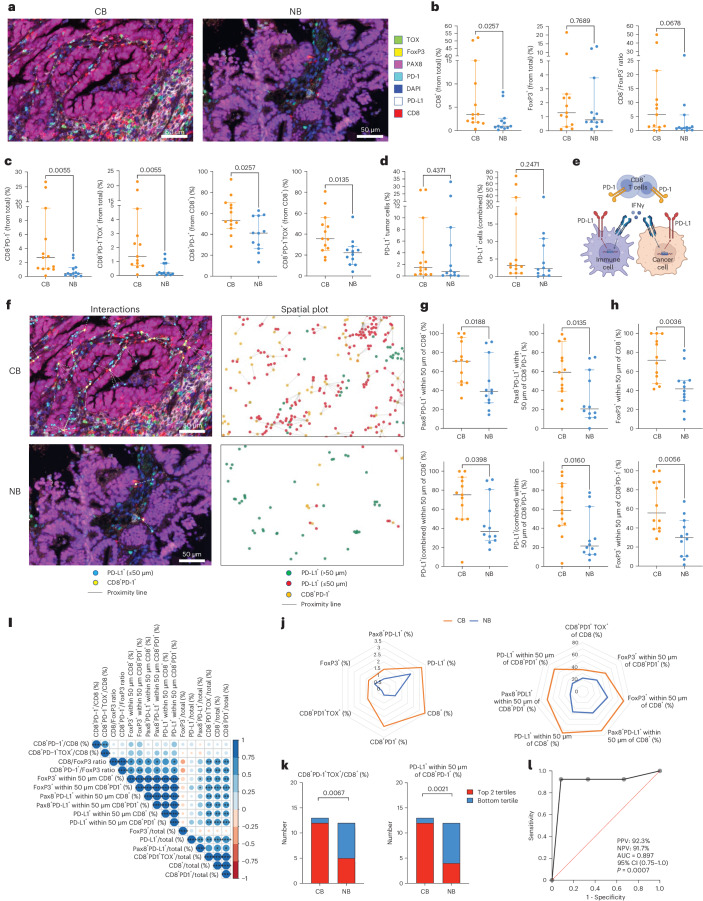
TME predictors of clinical benefit. Multiplex immunofluorescence imaging using the Vectra system was performed on archival tissue. Analyses were performed on the tumor compartment after excluding stroma. **a**, Representative tumor images from the clinical benefit (CB) and no benefit (NB) patients. Markers assessed in the panel are shown. **b**, Association of CD8^+^ T cells, FoxP3^+^ Treg cells and CD8^+^/FoxP3^+^ ratios with CB. **c**, Association of dysfunctional T cells (CD8^+^PD-1^+^) and terminally dysfunctional T cells (CD8^+^PD-1^+^TOX^+^) with CB. Relative percentages of the indicated populations out of total cells (left two panels) and out of CD8^+^ T cells (right two panels) are shown. **d**, Association of PD-L1 positivity in tumor cells (Pax8^+^) or tumor and immune cells (combined) with CB. **e**, Schematic of CD8^+^ T cell interactions with PD-L1^+^ cells in the TME. **f**, Representative interaction maps of CD8^+^PD-1^+^ T cells with PD-L1^+^ cells in the TME. Spatial plots accounting for all PD-L1^+^ T cells in the TME are shown on the right. **g**, Interaction CD8^+^ T cells with PD-L1^+^ tumor cells (top) and all PD-L1^+^ cells (bottom). **h**, Interaction of CD8^+^ T cells and dysfunctional CD8^+^ T cells with FoxP3^+^ cells in the TME. **i**, Correlation plot of the TME parameters using hierarchical clustering. **j**, Overall association of TME parameters with CB. **k**, Association of percentage of CD8^+^PD-1^+^TOX^+^ T cells and proximity of CD8^+^PD-1^+^ T cells to PD-L1^+^ cells with CB using bottom tertiles as cutoffs. **l**, ROC curve using percentage of CD8^+^PD-1^+^TOX^+^ T cells and proximity of CD8^+^PD-1^+^ T cells to PD-L1^+^ cells as variables. Figure 3b–d,g: *n* = 25; Fig. 3h: *n* = 24. Measure of center represents the median, with error bars representing 95% CI. Two-sided *P* value by Mann–Whitney test without multiple comparison adjustment is shown. Figure 3i: Pearson correlation without multiple comparison adjustment is indicated by circle size and color. *P* < 0.05, **P < 0.01, ***P < 0.001 and ****P < 0.0001, all two-sided. Figure 3k: *n* = 25, chi-square statistical comparisons are shown, two-sided *P* value. NPV, negative predictive value; PPV, positive predictive value. Image in panel (**e**) was created with BioRender.

**Extended Data Fig. 1:**
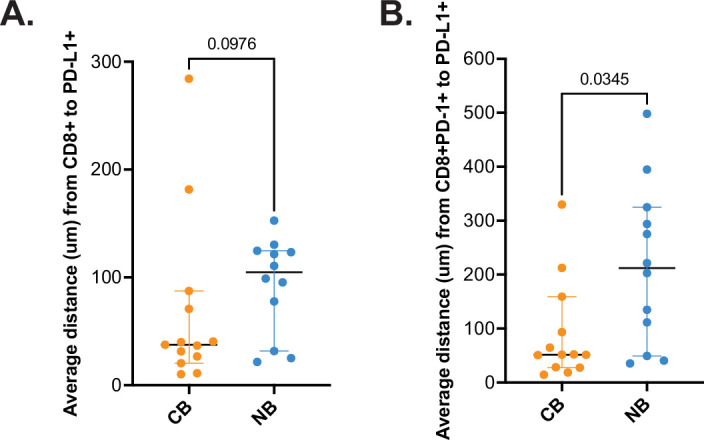
Proximity between T cells and the nearest PD-L1+ cells. Extended Data Fig. 1 Proximity between T cells and the nearest PD-L1+ cells. Average distances from the CD8+ T cell. (**a**) or CD8+PD-1+ T cell (**b**) to the nearest PD-L1+ cell in um were calculated in each sample. Measure of centre represents the median with error bars representing 95% CI. Two-sided *P* value by Mann-Whitney test without multiple comparison adjustment is shown.

We further explored the pre-treatment immune phenotype as an exploratory objective. Archival formalin-fixed paraffin-embedded (FFPE) tissue was available from 25 patients for evaluation by multiplexed immunofluorescence microscopy imaging. We stratified patients based on the co-primary endpoint of PFS24—13 who had PFS24 (clinical benefit) and 12 who did not (no clinical benefit). Segmentation was performed to isolate tumor and stromal compartments, and quantification of the relative percentages of cell populations and their functional states and interactions was performed in the tumor compartment (Fig. 3a)^23^. Overall CD8^+^ T cell infiltration was associated with clinical benefit (*P* = 0.026) in contrast to tumor infiltration with regulatory T cells (FoxP3^+^) or CD8^+^/FoxP3 ratio, which were not (Fig. 3b). When focusing on CD8^+^ T cell functional states, we specifically assessed the expression of PD-1, a marker of chronic antigen stimulation and dysfunction that was demonstrated to enrich for tumor-specific T cells across a number of cancers^24–27^, and TOX, a transcriptional master regulator responsible for terminal T cell dysfunction^20^. Increases in both dysfunctional (CD8^+^PD-1^+^) and terminally dysfunctional (CD8^+^PD-1^+^TOX^+^) T cells were strongly associated with clinical benefit (*P* = 0.006 and *P* = 0.001, respectively), with a large proportion of the CD8^+^ T cell compartment being dysfunctional or terminally dysfunctional in the patients who benefited (Fig. 3c). Expression of PD-L1 was not associated with response, neither when looking at PD-L1 expression in Pax8^+^ tumor cells nor combined (tumor and immune cell) PD-L1 expression (Fig. 3d). Because PD-L1 expression is a dynamic marker upregulated in response to interferon-gamma (IFN-γ) secretion by CD8^+^ T cells, we hypothesized that upregulation of PD-L1 in proximity of CD8^+^ T cells or dysfunctional CD8^+^ T cells could serve as a surrogate marker of T cell activation (Fig. 3e and Extended Data Fig. 1). Tumors from patients with clinical benefit exhibited closer proximity between the CD8^+^PD-1^+^ cells and the nearest PD-L1^+^ cells, with a median distance of 52 μm versus 212 μm in patients without benefit (Extended Data Fig. 1). Based on the reported distance of T-cell-produced IFN-γ action on the neighboring cells estimated to be at 30–40 μm (ref. ^28^), we examined the interaction of PD-L1^+^ cells with CD8^+^ and CD8^+^PD-1^+^ T cells using 50 μm as a cutoff. Both interactions were strongly associated with clinical benefit (Fig. 3f,g). By the same analogy, the interaction of regulatory T cells with CD8^+^ and CD8^+^PD-1^+^ T cells was strongly associated with clinical benefit (Fig. 3h). Due to association of multiple variables with clinical benefit, as an additional post hoc analysis, we built a multiple logistic regression model based on the relative magnitude of difference for each parameter between those who did and did not benefit and the correlation between the parameters to identify a set of variables independently associated with clinical benefit. We found minimal correlation between the percentage of CD8^+^PD-1^+^TOX^+^ T cells out of the total CD8 population (parameter 1) and percentage of PD-L1^+^ cells within 50 μm of CD8^+^PD-1^+^ T cells (parameter 2) (Fig. 3i). Based on the magnitude of difference between these parameters in the two groups (Fig. 3c,g,j), bottom tertiles (41% for parameter 1 and 25.6% for parameter 2) were selected as cutoffs for each of the biomarkers to differentiate clinical benefit from no benefit (Fig. 3k). A receiver operating characteristic (ROC) curve was used to plot the sensitivity along the *y* axis and the ‘1-Specificity’ along the *x* axis for multivariate model prediction of clinical benefit as outcome, resulting in an area under the curve (AUC) of 0.897 (*P* = 0.0007), demonstrating a strong ability of these variables to predict immunotherapy outcomes in these patients (Fig. 3l).

### Genomic analyses

**Fig. 4:**
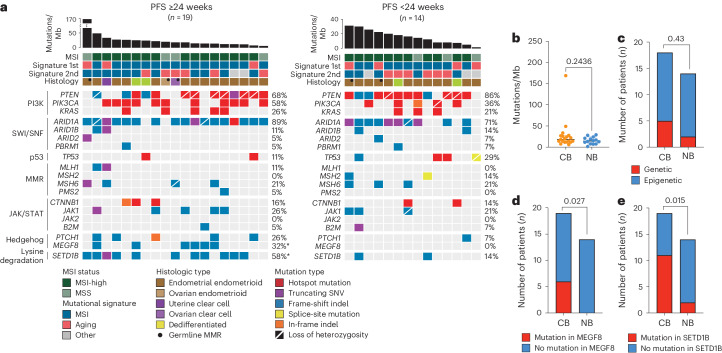
Somatic mutations in ovarian and endometrial cancers according to clinical benefit. **a**, TMB, MSIsensor score, mutational signatures and recurrent non-synonymous somatic mutations identified by whole-exome sequencing in endometrial cancers with clinical benefit (PFS ≥ 24 weeks) and without clinical benefit (PFS < 24 weeks). Cases are represented by columns and genes by rows. Only pathogenic mutations are shown. MSI status, dominant mutational signature, histologic types and mutation types are color-coded according to the legend. Percentages in bold indicate statistically significantly different. *P < 0.05, two-sided Fisherʼs exact test. First, dominant mutational signature; Second, secondary mutational signature. **b**, Association of CB (defined by PFS24) with TMB. **c**, Association of type of dMMR (genetic versus epigenetic) with CB (*n* = 32; one patient had no information available). **d**,**e**, Association of mutations in *MEGF8* and *SETD1B* with CB (*n* = 33). Figure 4b: measure of center represents the median, with error bars representing 95% CI. Two-sided *P* value by Mann–Whitney test is shown. Figure 4c–e: chi-square statistical comparisons are shown, two-sided *P* value. No multiple comparison adjustment was used for any of the indicated analyses. CB, clinical benefit; NB, no benefit.

**Extended Data Fig. 2:**
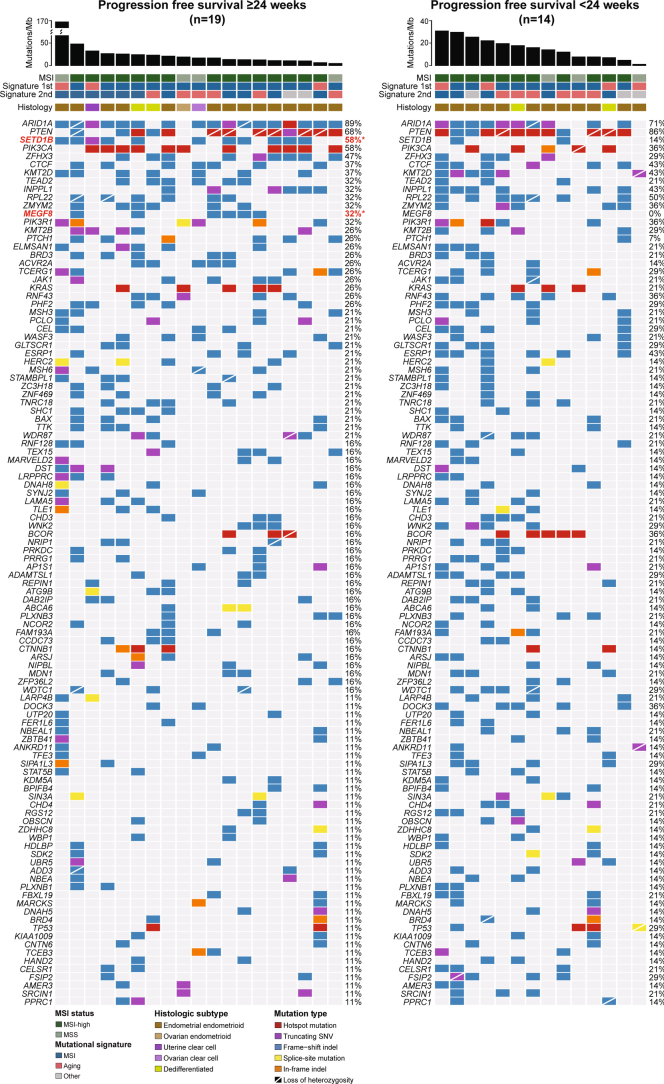
Full mutation Oncoprint for the entire patient cohort. Extended Data Fig. 2 Full mutation Oncoprint for the entire patient cohort. Patients are separated on the basis of clinical benefit, defined by progression-free survival (PFS) ≥24 weeks (n = 19) and PFS < 24 weeks (n = 14).

As an exploratory objective, we correlated the somatic mutational burden with clinical benefit from nivolumab. Tumors from 33 of 34 evaluable patients were subjected to whole-exome sequencing. In the overall cohort, somatic mutations affecting the PI3K signaling pathway, including *PTEN* (76%) and *PIK3CA* (48%); the SWI/SNF chromatin-remodeling genes, including *ARID1A* (82%); the JAK/STAT signaling pathway, including *JAK1* (24%) and *CTNNB1* (15%); and the Hedgehog signaling pathway, including *MEGF8* (18%) and *PTCH1* (18%), were found (Fig. 4a and Extended Data Fig. 2). Ten cases (30%) harbored pathogenic somatic mutations affecting an MMR gene, including *MLH1*, *MSH2*, *MSH6* or *PMS2*. Whole-exome sequencing-based MSI analysis revealed that 79% (26 of 33) of endometrial/ovarian cancers were MSI-H, whereas seven endometrial cancers (21%) were MSS. Mutational signature analysis further showed that 79% (26 of 33) of tumors had a dominant mutational signature related to dMMR (that is, signatures 6, 15 and 20; Fig. 4a). Of the seven endometrial/ovarian cancers with a dominant aging-related mutational signature, six had a secondary dMMR signature (Fig. 4a). Notably, the case with the highest TMB in this cohort was MSS; had a dominant aging-related, a secondary dMMR-related and a polymerase epsilon (*POLE)*-related mutational signature; and harbored a pathogenic *POLE* exonuclease domain hotspot mutation (p.F367S (ref. ^29^)), in addition to a pathogenic *MSH6* somatic mutation. Comparison between endometrial/ovarian cancers from patients who had clinical benefit (PFS ≥ 24 weeks; *n* = 19) versus those who did not (PFS < 24 weeks; *n* = 14) revealed no statistically significant differences in the TMB (18.1 versus 14.4, *P* = 0.24) (Fig. 4b), MSI status (79% versus 79% MSI-H, *P* = 1) and dominant dMMR mutational signature (84% versus 71%, *P* = 0.374). When focusing only on patients with dMMR and available mechanism of dMMR (genetic versus epigenetic; *n* = 32), type of dMMR was not associated with clinical benefit (*P* = 0.43) (Fig. 4c and Extended Data Table 2).

**Extended Data Fig. 3:**
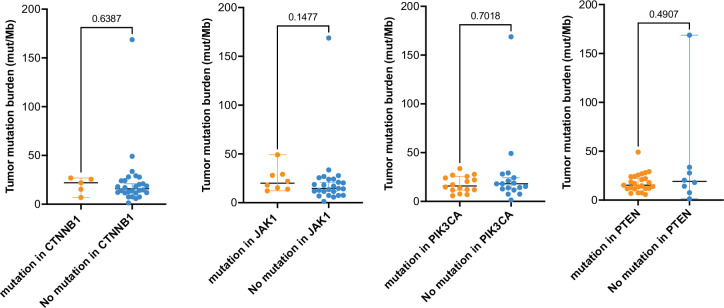
Association of individual mutations with tumor mutational burden (TMB). Extended Data Fig. 3 Association of individual mutations with tumor mutational burden (TMB). Presence or absence of the individual mutations known to be associated with immune resistance in other cancers was plotted against TMB. Measure of centre represents the median with error bars representing 95% CI. Two-sided *P* value by Mann-Whitney test without multiple comparison adjustment is shown.

**Extended Data Fig. 4:**
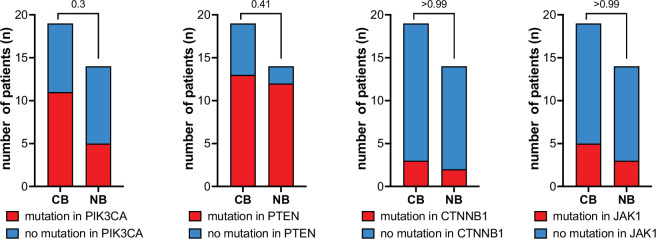
Association of individual mutations with clinical benefit. Extended Data Fig. 4 Association of individual mutations with clinical benefit. Presence or absence of the individual mutations known to be associated with immune resistance in other cancers was compared between patients with clinical benefit vs no benefit. N = 33, Chi-square statistical comparisons without multiple comparison adjustment are shown, two-sided *P* value. CB, clinical benefit; NB, no benefit.

**Extended Data Fig. 5:**
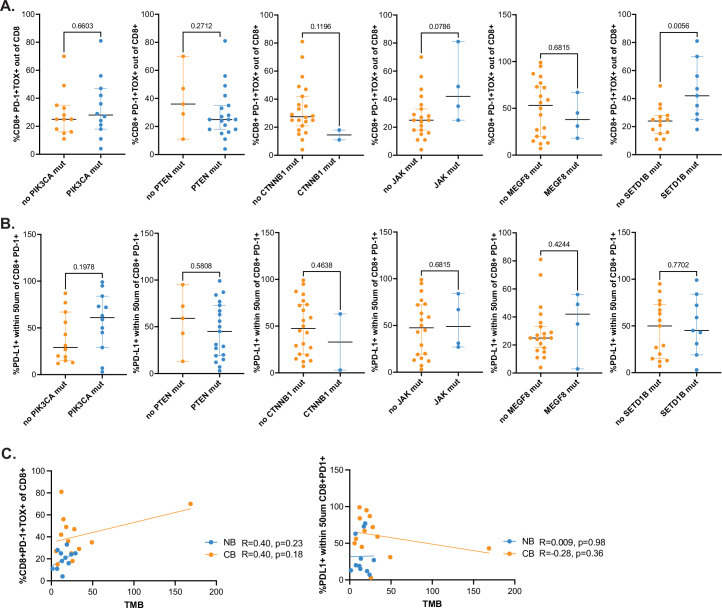
Association of molecular alterations with immune phenotypes. Extended Data Fig. 5 Association of molecular alterations with immune phenotypes. Presence or absence of the individual mutations known to be associated with immune resistance in other cancers was compared to the immune phenotypes associated with clinical benefit in the current study. **a**. Association between the individual mutations and CD8+PD-1+TOX+ T cells. **b**. Association between the individual mutations and interaction between CD8+PD-1+ T cells and PD- L1+ cells. (**a**,**b**): N = 24. Statistical comparisons were performed using Mann-Whitney test. Measure of centre represents the median with error bars representing 95% CI. Two-sided *P* value by Mann-Whitney test without multiple comparison adjustment is shown. **c**. Association between tumor mutational burden and immune phenotypes. Pearson correlation coefficients with two-sided *P* values are shown (N = 24).

Alterations in PI3K and beta-catenin signaling pathway-related genes, as well as mutations in *JAK1* and *JAK2*, were previously reported to be associated with resistance to immunotherapy in melanoma^30^. We performed a post hoc analysis focusing on these specific alterations in our cohort, but we observed no strong association between the mutations in *PIK3CA*, *PTEN, JAK* or *CTNNB1* and TMB (Extended Data Fig. 3) or with clinical benefit (Extended Data Fig. 4). Furthermore, none of these mutations was significantly associated with immune recognition, as defined by the percentage of CD8^+^PD-1^+^TOX^+^ T cells and interaction between CD8^+^PD-1^+^ T cells and PD-L1^+^ cells, although these analyses were limited by small group sizes (Extended Data Fig. 5). Finally, we observed no strong correlation between the TMB and the immune markers in patients with or without clinical benefit, although these analyses were limited by small numbers (Extended Data Fig. 5). We did observe, however, that mutations affecting the Hedgehog signaling pathway, including *MEGF8* (32% in patients with PFS ≥ 24 weeks versus 0% in patients with PFS < 24 weeks, *P* = 0.027) and SET domain-containing 1B (*SETD1B)*, a gene encoding histone lysine methyltransferase (58% in patients with PFS ≥ 24 weeks versus 14% in patients with PFS < 24 weeks, *P* = 0.015), were statistically significantly different between the tumors from patients who had clinical benefit versus those who did not (Fig. 4d,e). Mutations in *SETD1B* were also strongly associated with increased tumor infiltration with CD8^+^PD-1^+^TOX^+^ T cells (Extended Data Fig. 5), highlighting a potential link between this genetic alteration and immune recognition.

## Discussion

In this investigator-initiated phase 2 study, the use of nivolumab for dMMR/MSI-H endometrial or ovarian cancer met the pre-specified endpoint for clinical efficacy, with an ORR of 57% in the evaluable population and 64.7% of patients remaining progression free at 24 weeks. Response to nivolumab was observed in patients regardless of mechanism of dMMR or histologic subtype. Patients benefitted from substantial disease control, with median DOR and OS not yet reached with nearly 3 years of median follow-up. No new safety signals were noted, although rare immune-mediated toxicities, including myocarditis and type 1 diabetes, were seen.

Anti-PD-1/PD-L1 monotherapy has demonstrated robust clinical activity in patients with dMMR/MSI-H tumors^6,31–33^, both as a tissue-agnostic indication as well as specifically in endometrial cancer. Unfortunately, up to 60% of patients fail to respond or have progression of disease within 6 months. There remains a need to identify additional biomarkers for response and resistance, even in a favorable patient population, especially given the available data to support adding pembrolizumab or dostarlimab to platinum-based chemotherapy for patients with advanced or recurrent disease^12,13^.

TMB was used in several studies as a biomarker for response to immunotherapy^34,35^, and pembrolizumab now has an FDA-approved indication for advanced solid tumors with a TMB of ≥10 mutations per megabase (mut/Mb) as measured by the FoundationOne CDx assay. This cutoff was based on studies in lung cancer and has not been rigorously investigated in gynecologic malignancies. TMB has been conceived as a surrogate for tumor neoantigens; however, the quantity of mutations might not be directly related to the quality of mutation necessary to generate a robust T cell response. Moreover, available TMB assays have not been harmonized, leading to inconsistent scoring^36^. Recent data suggest that small insertions and deletions generate a much stronger immune response compared to single-nucleotide variants (SNVs)^37^. In our cohort, no significant difference was observed in TMB between responders and non-responders, suggesting that, among patients with dMMR/MSI tumors, TMB has no additional benefit in predicting likely response.

PD-L1 expression, in immune cells, tumor cells or both, has been used as a biomarker for response to PD-1/PD-L1 blockade in several solid tumor types, including lung, cervix and bladder cancers^38,39^. The value of PD-L1 as a predictive biomarker in dMMR cancers is unclear, and, in our dataset, PD-L1 expression was not predictive of objective response (Fig. 3d). This finding is consistent with previously published studies on PD-L1^+^ endometrial cancer regardless of dMMR/MSI status. In these studies, the ORR to single-agent PD-L1 blockade was modest, with ORRs ranging from 13% to 23% (refs. ^40–42^). Similarly, data from multiple cancer types have highlighted the prognostic and occasionally predictive value of tumor-infiltrating lymphocytes (TILs). In our dataset, the presence of CD8^+^ T cells was associated with response (Fig. 3), although association was not strong. Emerging data indicate that T cell functional states, rather than T cell numbers, serve as better biomarkers of tumor immunogenicity and potentially response to immune checkpoint blockade^18,43,44^. Chronic antigen exposure within the context of the TME leads to progressive differentiation of T cells through the stages of activation, early dysfunction and terminal dysfunction, characterized by progressive upregulation of transcriptional programs and surface receptors, such as PD-1, that dampen T cell function^45^. The transcription factor TOX has been identified as the master regulator driving the molecular and epigenetic programs of terminal T cell dysfunction/exhaustion. Several studies have demonstrated that expression of exhaustion markers by T cells is strongly predictive of tumor antigen reactivity. Interestingly, in our dataset, increased proportion of dysfunctional T cells and terminally dysfunctional T cells, defined as CD8^+^PD-1^+^ and CD8^+^PD1^+^TOX^+^, respectively, was significantly correlated with response^20^.

As an additional measure of T cell functional states, we examined interaction of CD8^+^ T cells with PD-L1^+^ cells in the TME without distinguishing whether PD-L1 was expressed by cancer cells or cancer-associated myeloid cells. Although PD-L1 expression alone was not predictive of response, the proximity of PD-L1^+^ cells to CD8^+^ TILs was predictive. These findings resonate with recently reported findings in ovarian cancer, in which co-localization of CD8^+^PD-1^+^ T cells with PD-L1-expressing myeloid cells was found to be important for T cell licensing^46^. Findings by Färkkilä et al.^47^ demonstrated that spatial proximity between PD-1^+^ TILs with PD-L1^+^ myeloid cells was associated with improved response to niraparib and pembrolizumab in patients with ovarian cancer.

In an exploratory genomic analysis, we found that alterations in *MEGF8* and *SETD1B* were enriched in patients who derived benefit from nivolumab. *MEGF8* acts as a negative regulator of the Hedgehog signaling pathway^48^. Recently, Hedgehog signaling was shown to modulate the TME by inducing immunosuppressive mechanisms, such as upregulating PD-L1 expression and recruiting immunosuppressive cell populations, including myeloid-derived suppressor cells (MDSCs) and regulatory T cells (Tregs)^49^. *SETD1B* is an important component of the histone methyltransferase complex that generates trimethylated histone H3 at Lys4 and has been implicated in multiple biological processes^50^. Interestingly, a related histone methyltransferase SETDB1 was recently demonstrated to play a role in anti-tumor immunity by regulating expression of endogenous retroelements^51^. In other tumor models, mutations in the SWI/SNF chromatin remodeling complex, including *PBRM1* and *ARID2*, have been found to sensitize tumor cells to T-cell-mediated killing^52,53^. In this cohort, we did not identify any significant difference in the rates of *PBRM1* and *ARID2* mutations between patients who did and did not derive clinical benefit. The enrichment of *SETD1B* mutations in patients who derived clinical benefit, however, suggests that chromatin remodeling may play a larger role in mediating sensitivity to PD-1 blockade beyond the SWI/SNF complex and should be explored further in larger cohorts of patients with dMMR/MSI tumors.

In dMMR/MSI colorectal cancer and melanoma, genomic alterations in antigen-presenting machinery, including loss of beta-2-microglobulin and human leukocyte antigen (HLA) genes, are associated with resistance to checkpoint blockade^54–56^. In this cohort, we did not note enrichment of any genomic alterations previously implicated in immunotherapy resistance, including *JAK*^56,57^*, CTNNB1* and *PTEN*^58^. Some previous studies also suggested that patients with Lynch syndrome may have a superior response to pembrolizumab^59^, and a study by Chow et al.^15^ demonstrated that genetic, rather than epigenetic, mechanism of dMMR was associated with stronger response to pembrolizumab in patients with dMMR endometrial cancer. Contrary to these findings, in our cohort, no significant difference was observed in the ORR among patients with germline or somatic dMMR alterations and those with *MLH1* hypermethylation.

Our study had several limitations, including a small sample size and access to limited archival tissue, which limited our ability to perform additional correlative studies. In addition, our population may not be reflective of the broader population of patients with dMMR/MSI-H tumors. Most patients in our cohort had high-grade disease (endometrioid or undifferentiated/dedifferentiated), which differs from the published literature, where only 29% of patients with dMMR endometrial cancer had high-grade disease^60^. Lastly, given the small number of patients with Lynch syndrome (*n* = 5), we cannot make any definitive conclusions about this population and how responses may differ from patients with somatic dMMR disease.

In summary, nivolumab achieved a high ORR and durable PFS with acceptable toxicity in patients with dMMR/MSI-H recurrent endometrial or ovarian cancer. In this dMMR-selected patient population, previously described immunotherapy response biomarkers, such as TMB or PD-L1 expression, were not associated with objective response or PFS. Instead, we identified two potential biomarkers associated with PFS in our cohort, including presence of dysfunctional T cells and spatial proximity between T cells and PD-L1-expressing cells in the TME. These parameters could be adaptable for future testing using clinically available immunohistochemistry assays, in which evaluation of 3–4 markers per slide (CD8, PD-1, TOX and PD-L1) would be sufficient. Overall, our findings highlight markers of pre-existing T cell response, as defined by T cell functionality, that may present a strategy for patient selection for anti-PD-1 therapy in dMMR gynecologic cancers and generate rationale for validation of these markers in larger cohorts of patients with dMMR disease.

## Methods

### Study design and procedures

This was a single-center, investigator-initiated, single-arm, phase 2 study conducted at MSK. This study was approved by the institutional review board at MSK and was registered at ClinicalTrials.gov (NCT03241745). This study was performed in accordance with the International Conference on Harmonization of Good Clinical Practice guidelines and the principles of the Declaration of Helsinki. All patients provided informed consent. Sex and/or gender were not considered in the trial design, but all participants were female because of the disease types enrolled. No compensation was provided for participation. The first patient consented on 27 September 2017, and the final patient consented on 24 May 2021. The trial completed as of 1 July 2022. The MSK Data and Safety Monitoring Committee provided independent monitoring for safety, data integrity and study conduct from the enrollment of the first participant until all patients completed treatment. The most recent version of the protocol is provided in the Supplementary Information.

The study underwent two noteworthy amendments. The first, dated 24 April 2018, clarified that clear cell carcinoma and endometrioid carcinoma were eligible histologies; decreased the hemoglobin eligibility value from 9 g dl^−1^ to 8 g dl^−1^; clarified that urinalysis could be collected at physician discretion; and added the collection of cell-free DNA (cfDNA) at specified timepoints. The second, dated 30 October 2018, changed the nivolumab dosing from 240 mg intravenously every 2 weeks to 480 mg every 4 weeks as per the Investigational Brochure Version 16.0; removed day 15 visits after cycle 1; and added primary peritoneal cancers as a possible eligible cancer site.

### Treatments and follow-up

Nivolumab was administered as 240 mg intravenously every 2 weeks until progression or unacceptable toxicity; this was amended on 30 October 2018 to reflect an updated dosing schedule of 480 mg every 4 weeks. Anti-tumor activity was assessed through radiologic tumor assessments conducted at baseline of starting therapy and every 12 weeks thereafter. RECIST version 1.1 were used to determine response and progression^61^. Toxicity data were collected at each visit and classified according to the National Cancer Institute Common Terminology Criteria for Adverse Events (CTCAE) version 5.0.

### Eligibility

#### Patient inclusion criteria included the following

1. Histologically confirmed diagnosis of metastatic or recurrent uterine cancer (endometrial carcinoma, carcinosarcoma, clear cell carcinoma, leiomyosarcoma, undifferentiated sarcoma and high-grade endometrial stromal sarcoma) by MSK. Carcinosarcomas and endometrioid and clear cell carcinomas that appeared to have arisen in the ovary/fallopian tube or peritoneum were also eligible. Recurrences could not be amenable to curative approaches, such as surgical resection or chemoradiotherapy.
2. Tumor was confirmed to be one of the following: MSI-H or dMMR or hypermutated defined as ≥20 somatic mutations in the tumor by MSK-IMPACT.
3. One or more prior lines of cytotoxic treatment for advanced disease (prior hormonal therapy was not considered to count as prior lines of therapy).
4. Measurable disease by RECIST version 1.1.
5. No known central nervous system metastases.
6. Eastern Cooperative Oncology Group (ECOG) performance status between 0 and 1.
7. White blood cell count more than 2,000 per microliter, absolute neutrophil count (ANC) more than 1,500 per microliter, platelet count more than 100,000 per microliter and hemoglobin level more than 8 g dl^−1^.
8. Serum creatinine less than 1.5× the upper limit of normal (ULN) or creatinine clearance of more than 40 ml min^−1^ by the Cockroft–Gault formula.
9. Aspartate aminotransferase (AST/SGOT) and alanine aminotransferase (ALT/SGPT) less than 3× ULN.
10. Total bilirubin less than 1.5× ULN, except patients with Gilbert’s syndrome who could have total bilirubin less than 3.0 mg dl^−1^.
11. Able to sign voluntary written informed consent.
12. Female, 18 years of age or older.
13. Available archival tumor tissue or patient was willing to undergo new biopsy.
14. Premenopausal women of childbearing potential must have had a normal urine or serum beta-human chorionic gonadotropin (HCG) before enrollment and must have agreed to use effective contraception during treatment with nivolumab and for at least 5 months after the last dose of nivolumab.

Of note, all patients were assigned as female based on sex at birth given the diseases studied; to our knowledge, none of the patients self-identified as another gender.

#### Patient exclusion criteria included the following

1. Disease eligible for potentially curative treatment with standard chemotherapy, surgical resection or chemoradiotherapy.
2. Known or suspected autoimmune disease, except for patients with vitiligo, diabetes mellitus, resolved childhood asthma/atopy, residual hypothyroidism due to an autoimmune immune condition only requiring thyroid hormone replacement, psoriasis not requiring systemic treatment or conditions not expected to recur in the absence of an external trigger.
3. Serious uncontrolled medical disorder or active infection that would impair the ability of the patient to receive protocol therapy or whose control would be jeopardized by protocol therapy.
4. History of bowel obstruction, refractory ascites or bowel perforation due to advanced disease within the past 3 months from start of study treatment.
5. Prior therapy with anti-PD-1, anti-PD-L1, anti-PD-L2, anti-CD137, anti-CTLA-4 antibody or any other antibody or drug specifically targeting T cell co-stimulation or immune checkpoint pathways.
6. Patients with a condition that requires systemic treatment with corticosteroids within 7 d of enrollment (systemic corticosteroid therapy is defined as more than 10 mg daily prednisone or its equivalent) or who required other immunosuppressive medications within 14 d of study drug administration. Inhaled or topical steroids and adrenal replacement doses more than 10 mg daily prednisone equivalents were permitted in the absence of active autoimmune disease.
7. Prior history of malignancy or a concurrent malignancy, with the exception of cutaneous basal cell carcinoma or squamous cell carcinoma, superficial bladder cancer or in situ carcinoma of the uterine cervix, prostate or breast, unless a complete remission was achieved at least 3 years before study entry and no additional therapy was required or anticipated to be required during the study period.
8. Breastfeeding women and pregnant women.
9. Prisoners or patients who are involuntarily incarcerated.
10. Patients who are compulsorily detained for treatment of either a psychiatric or a physical illness.
11. Positive test for hepatitis B virus surface antigen (HBV sAg) or hepatitis C virus ribonucleic acid (HCV antibody) indicating acute or chronic infection (if patient had documented hepatitis B or C from within 6 months of enrollment, these tests did not need to be repeated).
12. Known history of testing positive for HIV or known AIDS.
13. Known allergy or adverse drug reaction to nivolumab or a history of allergy to study drug components.

### Correlative assessments

#### Multiplexed immunofluorescence analysis

Primary antibody staining conditions were initially optimized using standard immunohistochemical analysis on the Leica Bond RX automated research stainer with DAB detection (Leica Bond Polymer Refine Detection DS9800). Using 4-µm FFPE tissue sections and serial antibody titrations, the optimal antibody concentration was determined, followed by transition to a seven-color multiplex assay with equivalency. Multiplex immunohistochemical analysis was performed on a Leica Bond RX automated research stainer with DAB detection (Leica Bond Polymer Refine Detection DS9800). The antibody panel included FOXP3 (236A/E7, Biocare), PD-L1 (1:400, E1L3N, Cell Signaling Technology), CD8 (4B11, 1:500, Leica), PAX8 (EPR18715, 1:1,000, Abcam), PD-1 (EPR4877(2), 1:400, Abcam), TOX (E6I3Q, 1:7,000, Cell Signaling Technology) as well as DAPI. The 4-µm FFPE tissue sections were baked for 3 h at 62 °C with subsequent deparaffinization performed on the Leica Bond RX, followed by six sequential cycles of staining, with each round including a 30-min combined block and primary antibody incubation (PerkinElmer antibody diluent/block ARD1001). For all antibodies, detection was performed using a secondary horseradish peroxidase (HRP)-conjugated polymer (PerkinElmer Opal polymer HRP Ms + Rb ARH1001, 10-min incubation). The HRP-conjugated secondary antibody polymer was detected using fluorescent tyramide signal amplification using Opal dyes 520 (TOX), 540 (PD-1), 570 (CD8), 620 (PD-L1), 650 (FoxP3) and 690 (PAX8) (Perkin Elmer FP1487a, FP1494a, FP1488a, FP1496a, FP1495a and FP1497a). The covalent tyramide reaction was followed by heat-induced stripping of the primary/secondary antibody complex using PerkinElmer AR9 buffer (AR900250ML) at 100 °C for 20 min preceding the next cycle. After six sequential rounds of staining, sections were stained with Hoechst (Invitrogen, 33342) to visualize nuclei and mounted with ProLong Gold antifade reagent mounting medium (Invitrogen, P36930). Seven-color multiplex-stained slides were imaged using the Vectra Multispectral Imaging System version 3 (PerkinElmer). Scanning was performed at ×20 (×200 final magnification). Filter cubes used for multispectral imaging were DAPI, FITC, Cy3, Texas Red and Cy5. A spectral library containing the emitted spectral peaks of the fluorophores in this study was created using Vectra image analysis software (PerkinElmer). Using multispectral images from single-stained slides for each marker, the spectral library was used to separate each multispectral cube into individual components (spectral unmixing), allowing for identification of the seven marker channels of interest using inForm 2.4 image analysis software.

#### Immunofluorescence analyses

Images were exported to Indica Labs HALO image analysis platform, and cell segmentation and signal thresholding were performed separately on each case using a supervised algorithm. The entire scanned slide area was analyzed with a median number of 66,935 DAPI^+^ cells (range, 641–164,584). Individual cell populations were quantified in both tumor and stromal compartments, with quantifications in tumor compartment reported. For individual cell populations, percentages out of total nucleated cells are reported unless otherwise specified. To quantify the cell interactions, average distances between the specified cells of interest were initially computed. The distance of 50 µm was arbitrarily chosen as a cutoff for further proximity analyses, as it represents a neighborhood of 2–3 cells and is consistent with a reported distance of T-cell-produced IFN-γ action on the neighboring cells, which is estimated to be 30–40 μm (ref. ^28^). This is similar to cutoffs used in studies of other cancer types^62^. To quantify the cell interactions, the number of the specified cells located within 50 µm of the interacting cells was divided by the total number of the specified cells quantified in the analyzed slide area. For example, to calculate the percentage of PD-L1^+^ cells that are located within 50 µm of CD8^+^PD-1^+^ cells, the number of PD-L1^+^ cells located within 50 µm of CD8^+^PD-1^+^ cells was divided by the total number of PD-L1^+^ cells in the analyzed area. For the cases in which more than one archival tumor was analyzed, data presented represent an average of measurements between the individual samples.

#### Whole-exome sequencing

Whole-exome recapture of tumor and patient-matched germline DNA libraries that previously underwent clinical FDA-authorized MSK-IMPACT targeted NGS was performed, as previously described^22,63^ Whole-exome sequencing data were analyzed as previously described^64^. Mutations affecting hotspot codons were annotated according to Chang et al.^65^. A somatic mutation was defined as pathogenic if it affected a mutational hotspot or was deleterious/loss-of-function in the case of tumor suppressor genes, as previously described^66^. deconstructSigs^67^ at default parameters was used to infer mutational signatures (COSMIC, version 3.1) using all SNVs of a given endometrial/ovarian cancer, as previously described^68^. MSIsensor was employed according to Niu et al.^69^, and samples with MSIsensor score ≥3.5 were considered MSI-H. TMB was calculated by dividing the number of non-synonymous mutations by the total size of the capture panel in megabases. Germline mutational status as indicated in Extended Data Table 2 was established via sequencing of matched normal blood using MSK-IMPACT assay after patient consent to germline analysis.

#### Statistical methods

The co-primary objectives were to define (1) PFS24 and (2) the proportion of patients who achieved objective tumor response (ORR) by RECIST version 1.1 (ref. ^61^). Secondary objectives included PFS, OS, safety and toxicity, DOR and DCR. Exploratory objectives were to (1) correlate the somatic mutational burden with ORR and PFS24; (2) correlate the somatic mutational burden with MSIsensor score; (3) correlate MSIsensor score with MMR immunohistochemistry status; and (4) correlate the pre-treatment immune phenotype with ORR and PFS24.

The sample size calculation for this study was based on a non-promising ORR of 5% and a promising ORR of 25%. To that end, we used a Simon two-stage minimax design. In the first stage, we enrolled 23 eligible patients, and at least two patients were required to achieve a response to proceed to stage II. In stage II, an additional 17 patients were enrolled. Among the total 40 patients, if six or more patients achieved a response, this treatment regimen would be declared promising. This decision rule had a type I error rate of 0.025 and a type II error rate of 0.05.

PFS24 was the co-primary endpoint. The sample size calculation for this study was based on a non-promising PFS24 of 25% and a promising PFS24 of 50%. Using a Simon two-stage minimax design, in the first stage, we enrolled 23 eligible patients; at least five patients of the initial 17 were required to be progression free at 24 weeks to proceed to stage II. In stage II, an additional 17 patients were enrolled. Among the total 40 patients, if 16 or more patients were progression free at 24 weeks, this treatment regimen would be declared promising. This decision rule had a type I error rate of 0.025 and a type II error rate of 0.09. The study continued to stage II if either ORR or PFS24 was promising. If there was one or fewer objective responses in stage I out of 23 patients, then accrual would be held to determine if at least five of 17 patients remained progression free at 24 weeks.

Patients were evaluable for efficacy if they had received at least one dose of therapy and had at least one post-baseline efficacy assessment. Patients who were evaluable for response and were lost to follow-up or died before the 24-week PFS assessment were considered events.

PFS was calculated from start of treatment to progression/recurrence or death or last follow-up, whichever occurred first. OS was calculated from start of treatment to death or last follow-up, whichever occurred first. DOR was calculated from time of response (for complete response or partial response) to progression, death or last follow-up. OS, PFS and DOR rates were estimated using the Kaplan–Meier method. Adverse events were tabulated.

The study opened to accrual on 3 August 2017. As per the Simon two-stage minimax design, the study met the criteria to continue to the second stage and was closed to accrual on 2 March 2022, because the primary endpoint of at least six objective responses was met with a final accrual of 35 patients.

Correlation of response with translational parameters was performed by dichotomizing patients based on PFS24, and distribution of the continuous biomarkers (for example, percentages of CD8^+^PD1^+^ cells) between the two groups was compared using the Mann–Whitney test. TMBs were compared using the Mann–Whitney test, and comparisons of frequency of mutations were performed using two-tailed Fisherʼs exact tests. For exploratory translational analyses, no adjustments for multiple comparisons were performed. Figures were generated using GraphPad Prism 9.5 and R 4.2.3 software.

### Reporting summary

Further information on research design is available in the Nature Portfolio Reporting Summary linked to this article.

## Online content

Any methods, additional references, Nature Portfolio reporting summaries, source data, extended data, supplementary information, acknowledgements, peer review information; details of author contributions and competing interests; and statements of data and code availability are available at 10.1038/s41591-024-02942-7.

### Supplementary information

Supplementary Information Full therapeutic protocol.

Reporting Summary

Supplementary Data 1 Antibody validations.

## Figures and Tables

**Extended Data Table 1:**
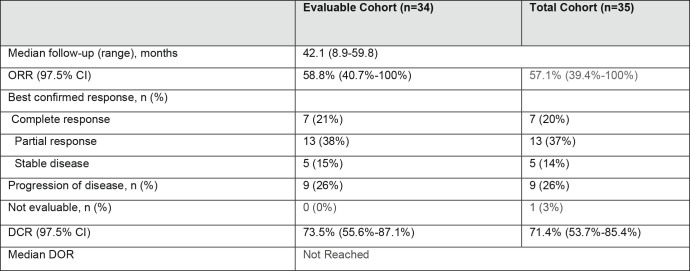
ORRs in patients with dMMR or MSI-H endometrial or ovarian cancer

Abbreviations: ORR, objective response rate; CI, confidence interval; DCR, disease control rate; DOR, duration of response.

**Extended Data Table 2:**
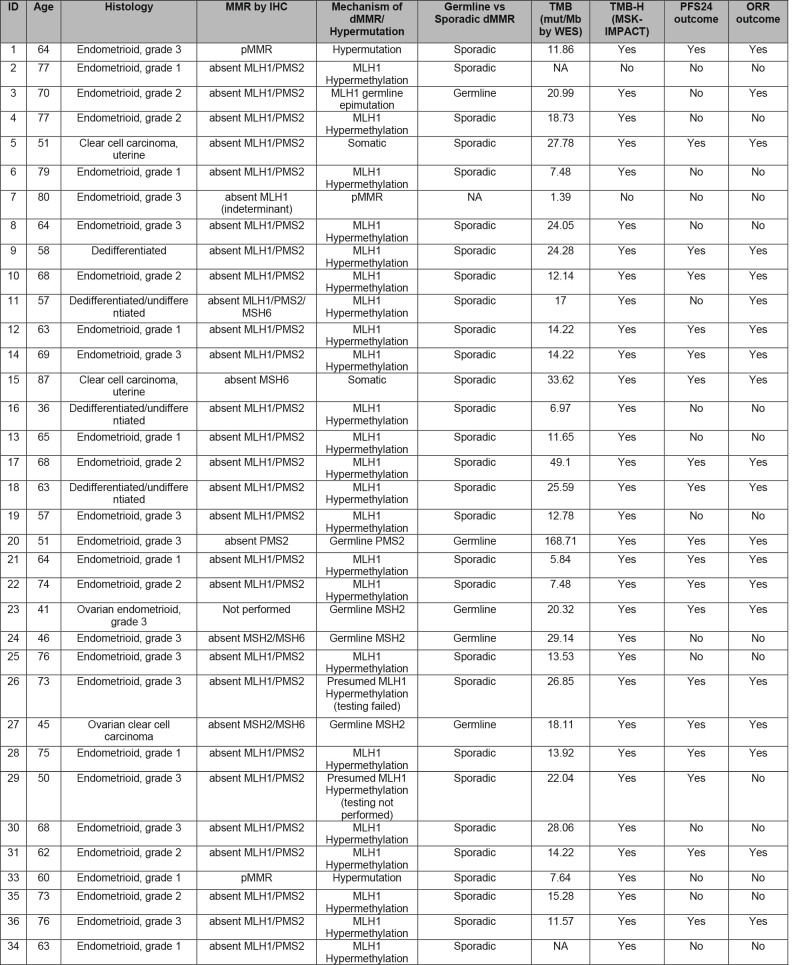
Association of clinical and dMMR characteristics with clinical outcomes

Abbreviations: MMR, mismatch repair; dMMR, mismatch repair deficient; MSI-H, microsatellite instability high; IHC, immunohistochemistry; pMMR, mismatch repair proficient; TMB, tumor mutational burden; PFS24, progression-free survival at 24 weeks; ORR, objective response rate; mut/Mb, mutations per megabase; WES, whole exome sequencing; TMB-H, high tumor mutational burden; MSK-IMPACT, Memorial Sloan Kettering Cancer Center-Integrated Mutation Profiling of Actionable Cancer Targets.

**Extended Data Table 3:**
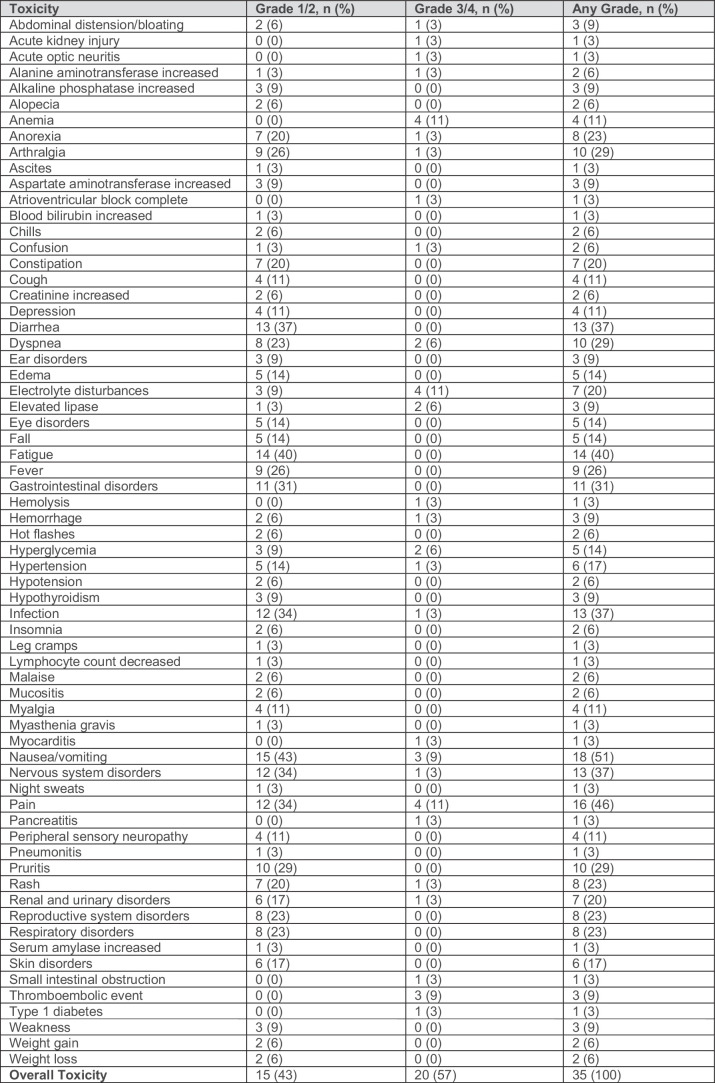
All TRAEs

**Extended Data Table 4:**
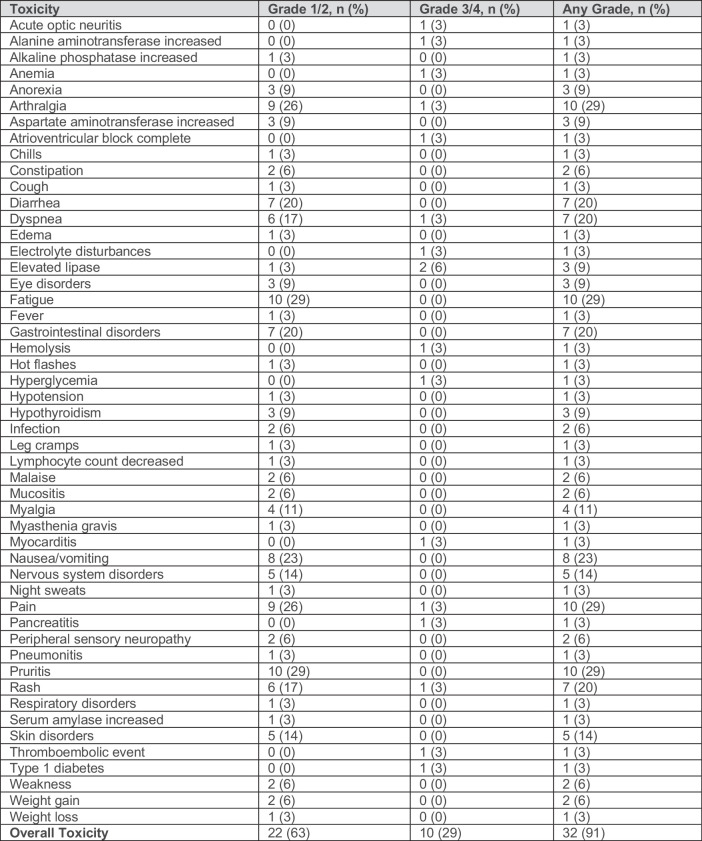
All TRAEs

**Extended Data Table 5:**
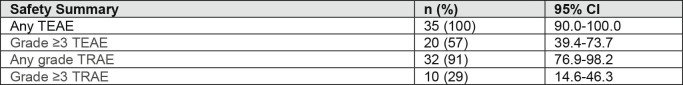
Summary of AEs

Abbreviations: TEAE, treatment-emergent adverse event; TRAE, treatment-related adverse event; CI, confidence interval.

## Data Availability

Datasets generated and analyzed in this study are available for general research use. The MSK-IMPACT dataset is available for browsing via cBioPortal (https://www.cbioportal.org/study/summary?id=ucec_msk_2024). MpIF images will be available from Synapse (https://www.synapse.org/#!Synapse:syn53699039/files/). Controlled-tier datasets requiring access approval are available by requesting authorization to the Data Access Committee via dbGAP (https://www.ncbi.nlm.nih.gov/projects/gap/cgi-bin/study.cgi?study_id=phs001783.v6.p1). External requests for the data (friedmac@mskcc.org) will be evaluated within a period of 2–3 weeks to ensure that they are in compliance with the data-sharing policies outlined in the informed consent.

## References

[1] Siegel RL, Miller KD, Wagle NS, Jemal A (2023). Cancer statistics, 2023. CA Cancer J. Clin..

[2] Muggia FM, Blessing JA, Sorosky J, Reid GC (2002). Phase II trial of the pegylated liposomal doxorubicin in previously treated metastatic endometrial cancer: a Gynecologic Oncology Group study. J. Clin. Oncol..

[3] Garcia AA, Blessing JA, Nolte S, Mannel RS (2008). A phase II evaluation of weekly docetaxel in the treatment of recurrent or persistent endometrial carcinoma: a study by the Gynecologic Oncology Group. Gynecol. Oncol..

[4] Miller DS, Blessing JA, Lentz SS, Waggoner SE (2002). A phase II trial of topotecan in patients with advanced, persistent, or recurrent endometrial carcinoma: a Gynecologic Oncology Group study. Gynecol. Oncol..

[5] Levine DA (2013). Integrated genomic characterization of endometrial carcinoma. Nature.

[6] Le DT (2017). Mismatch-repair deficiency predicts response of solid tumors to PD-1 blockade. Science.

[7] Xiao X, Melton DW, Gourley C (2014). Mismatch repair deficiency in ovarian cancer—molecular characteristics and clinical implications. Gynecol. Oncol..

[8] Le DT (2015). PD-1 blockade in tumors with mismatch-repair deficiency. N. Engl. J. Med..

[9] Ott PA (2017). Safety and antitumor activity of pembrolizumab in advanced programmed death ligand 1-positive endometrial cancer: results from the KEYNOTE-028 study. J. Clin. Oncol..

[10] Marcus L, Lemery SJ, Keegan P, Pazdur R (2019). FDA approval summary: pembrolizumab for the treatment of microsatellite instability-high solid tumors. Clin. Cancer Res..

[11] US Food and Drug Administration. Highlights of prescribing information: JEMPERLI. https://www.accessdata.fda.gov/drugsatfda_docs/label/2021/761174s000lbl.pdf (2021).

[12] Mirza MR (2023). Dostarlimab for primary advanced or recurrent endometrial cancer. N. Engl. J. Med..

[13] Eskander RN (2023). Pembrolizumab plus chemotherapy in advanced endometrial cancer. N. Engl. J. Med..

[14] Manning-Geist BL (2022). Microsatellite instability–high endometrial cancers with *MLH1* promoter hypermethylation have distinct molecular and clinical profiles. Clin. Cancer Res..

[15] Chow RD (2023). Distinct mechanisms of mismatch-repair deficiency delineate two modes of response to anti-PD-1 immunotherapy in endometrial carcinoma. Cancer Discov..

[16] Gulhan DC (2020). Genomic determinants of de novo resistance to immune checkpoint blockade in mismatch repair-deficient endometrial cancer. JCO Precis. Oncol..

[17] Patel SP, Kurzrock R (2015). PD-L1 expression as a predictive biomarker in cancer immunotherapy. Mol. Cancer Ther..

[18] Tumeh PC (2014). PD-1 blockade induces responses by inhibiting adaptive immune resistance. Nature.

[19] Huang AC (2017). T-cell invigoration to tumour burden ratio associated with anti-PD-1 response. Nature.

[20] Scott AC (2019). TOX is a critical regulator of tumour-specific T cell differentiation. Nature.

[21] Middha, S. et al. Reliable pan-cancer microsatellite instability assessment by using targeted next-generation sequencing data. *JCO Precis. Oncol.* **2017**, PO.17.00084 (2017). 10.1200/PO.17.00084 PMC6130812 30211344

[22] Zehir A (2017). Mutational landscape of metastatic cancer revealed from prospective clinical sequencing of 10,000 patients. Nat. Med..

[23] Tan WCC (2020). Overview of multiplex immunohistochemistry/immunofluorescence techniques in the era of cancer immunotherapy. Cancer Commun. (Lond.).

[24] Li H (2019). Dysfunctional CD8 T cells form a proliferative, dynamically regulated compartment within human melanoma. Cell.

[25] Verma V (2019). PD-1 blockade in subprimed CD8 cells induces dysfunctional PD-1^+^CD38^hi^ cells and anti-PD-1 resistance. Nat. Immunol..

[26] Philip M, Schietinger A (2019). Heterogeneity and fate choice: T cell exhaustion in cancer and chronic infections. Curr. Opin. Immunol..

[27] Zhou J (2020). Clinical significance of CD38 and CD101 expression in PD-1^+^CD8^+^ T cells in patients with epithelial ovarian cancer. Oncol. Lett..

[28] Centofanti E (2023). The spread of interferon-γ in melanomas is highly spatially confined, driving nongenetic variability in tumor cells. Proc. Natl Acad. Sci. USA.

[29] León-Castillo A (2020). Interpretation of somatic *POLE* mutations in endometrial carcinoma. J. Pathol..

[30] Kalbasi A, Ribas A (2020). Tumour-intrinsic resistance to immune checkpoint blockade. Nat. Rev. Immunol..

[31] Oaknin A (2020). Clinical activity and safety of the anti–programmed death 1 monoclonal antibody dostarlimab for patients with recurrent or advanced mismatch repair–deficient endometrial cancer: a nonrandomized phase 1 clinical trial. JAMA Oncol..

[32] Le, D. T. et al. PD-1 blockade in tumors with mismatch repair deficiency. *J. Clin. Oncol.* 33, 10.1200/jco.2015.33.18_suppl.lba100(2015).

[33] Konstantinopoulos PA (2019). Phase II study of avelumab in patients with mismatch repair deficient and mismatch repair proficient recurrent/persistent endometrial cancer. J. Clin. Oncol..

[34] Marabelle A (2020). Association of tumour mutational burden with outcomes in patients with advanced solid tumours treated with pembrolizumab: prospective biomarker analysis of the multicohort, open-label, phase 2 KEYNOTE-158 study. Lancet Oncol..

[35] Yarchoan M, Hopkins A, Jaffee EM (2017). Tumor mutational burden and response rate to PD-1 inhibition. N. Engl. J. Med..

[36] Spouge JL (1987). Strong conformational propensities enhance T cell antigenicity. J. Immunol..

[37] Turajlic S (2017). Insertion-and-deletion-derived tumour-specific neoantigens and the immunogenic phenotype: a pan-cancer analysis. Lancet Oncol..

[38] Herbst RS (2015). Pembrolizumab versus docetaxel for previously treated, PD-L1-positive, advanced non-small-cell lung cancer (KEYNOTE-010): a randomised controlled trial. Lancet.

[39] Chung HC (2019). Efficacy and safety of pembrolizumab in previously treated advanced cervical cancer: results from the phase II KEYNOTE-158 study. J. Clin. Oncol..

[40] Ott PA (2017). Safety and antitumor activity of pembrolizumab in advanced programmed death ligand 1–positive endometrial cancer: results from the KEYNOTE-028 study. J. Clin. Oncol..

[41] Tamura K (2019). Efficacy and safety of nivolumab in Japanese patients with uterine cervical cancer, uterine corpus cancer, or soft tissue sarcoma: multicenter, open-label phase 2 trial. Cancer Sci..

[42] Liu JF (2019). Safety, clinical activity and biomarker assessments of atezolizumab from a phase I study in advanced/recurrent ovarian and uterine cancers. Gynecol. Oncol..

[43] Kim CG (2021). Distinct exhaustion features of T lymphocytes shape the tumor-immune microenvironment with therapeutic implication in patients with non-small-cell lung cancer. J. Immunother. Cancer.

[44] Huang AC (2019). A single dose of neoadjuvant PD-1 blockade predicts clinical outcomes in resectable melanoma. Nat. Med..

[45] Wherry EJ (2007). Molecular signature of CD8^+^ T cell exhaustion during chronic viral infection. Immunity.

[46] Cascio S (2021). Cancer-associated MSC drive tumor immune exclusion and resistance to immunotherapy, which can be overcome by Hedgehog inhibition. Sci. Adv..

[47] Färkkilä A (2020). Immunogenomic profiling determines responses to combined PARP and PD-1 inhibition in ovarian cancer. Nat. Commun..

[48] Zhang Q, Jiang J (2021). Regulation of Hedgehog signal transduction by ubiquitination and deubiquitination. Int. J. Mol. Sci..

[49] Grund-Gröschke S, Stockmaier G, Aberger F (2019). Hedgehog/GLI signaling in tumor immunity—new therapeutic opportunities and clinical implications. Cell Commun. Signal..

[50] Lee JH, Tate CM, You JS, Skalnik DG (2007). Identification and characterization of the human Set1B histone H3-Lys^4^ methyltransferase complex. J. Biol. Chem..

[51] Zhang SM (2021). KDM5B promotes immune evasion by recruiting SETDB1 to silence retroelements. Nature.

[52] Pan D (2018). A major chromatin regulator determines resistance of tumor cells to T cell–mediated killing. Science.

[53] Miao D (2018). Genomic correlates of response to immune checkpoint therapies in clear cell renal cell carcinoma. Science.

[54] Grasso CS (2018). Genetic mechanisms of immune evasion in colorectal cancer. Cancer Discov..

[55] Snahnicanova Z (2020). Genetic and epigenetic analysis of the beta-2-microglobulin gene in microsatellite instable colorectal cancer. Clin. Exp. Med..

[56] Zaretsky JM (2016). Mutations associated with acquired resistance to PD-1 blockade in melanoma. N. Engl. J. Med..

[57] Shin DS (2017). Primary resistance to PD-1 blockade mediated by *JAK1/2* mutations. Cancer Discov..

[58] Trujillo JA (2019). Secondary resistance to immunotherapy associated with β-catenin pathway activation or PTEN loss in metastatic melanoma. J. Immunother. Cancer.

[59] Bellone S (2022). A phase 2 evaluation of pembrolizumab for recurrent Lynch-like versus sporadic endometrial cancers with microsatellite instability. Cancer.

[60] Gordhandas S (2020). Clinicopathologic features of endometrial cancer with mismatch repair deficiency. Ecancermedicalscience.

[61] Eisenhauer EA (2009). New response evaluation criteria in solid tumours: revised RECIST guideline (version 1.1). Eur. J. Cancer.

[62] Tsakiroglou AM (2020). Spatial proximity between T and PD-L1 expressing cells as a prognostic biomarker for oropharyngeal squamous cell carcinoma. Br. J. Cancer.

[63] Cheng DT (2015). Memorial Sloan Kettering-Integrated Mutation Profiling of Actionable Cancer Targets (MSK-IMPACT): a hybridization capture-based next-generation sequencing clinical assay for solid tumor molecular oncology. J. Mol. Diagn..

[64] Safdar NS (2022). Genomic determinants of early recurrences in low-stage, low-grade endometrioid endometrial carcinoma. J. Natl Cancer Inst..

[65] Chang MT (2018). Accelerating discovery of functional mutant alleles in cancer. Cancer Discov..

[66] Moukarzel LA (2021). The genetic landscape of metaplastic breast cancers and uterine carcinosarcomas. Mol. Oncol..

[67] Rosenthal R, McGranahan N, Herrero J, Taylor BS, Swanton C (2016). deconstructSigs: delineating mutational processes in single tumors distinguishes DNA repair deficiencies and patterns of carcinoma evolution. Genome Biol..

[68] Ashley CW (2019). Analysis of mutational signatures in primary and metastatic endometrial cancer reveals distinct patterns of DNA repair defects and shifts during tumor progression. Gynecol. Oncol..

[69] Niu B (2014). MSIsensor: microsatellite instability detection using paired tumor-normal sequence data. Bioinformatics.

