# Supplementary material for: Nivolumab for mismatch-repair-deficient or hypermutated gynecologic cancers: a phase 2 trial with biomarker analyses
Source: Nat Med. 2024 Apr 23;30(5):1330–8. doi: 10.1038/s41591-024-02942-7 (PMC11108776; doi:10.1038/s41591-024-02942-7)
Supplement: Supplementary file 1 — Full therapeutic protocol. [file 41591_2024_2942_MOESM1_ESM.pdf]

nature medicine

Article

https://doi.org/10.1038/s41591-024-02942-7

Nivolumab for mismatch-repair-deficient or hypermutated gynecologic cancers: a phase 2 trial with biomarker analyses

In the format provided by the authors and unedited

MSK PROTOCOL COVER SHEET

Phase II Trial of Single-Agent Nivolumab in Patients with Microsatellite Unstable/Mismatch Repair Deficient/Hypermutated Uterine Cancer

Principal Investigator/Department:

Claire Friedman, MD / Medicine

Memorial Sloan Kettering Cancer Center
1275 York Avenue
New York, New York 10065

Table of Contents

1.0 PROTOCOL SUMMARY AND/OR SCHEMA 3
2.0 OBJECTIVES AND SCIENTIFIC AIMS 3
3.0 BACKGROUND AND RATIONALE 4
4.0 OVERVIEW OF STUDY DESIGN/INTERVENTION 104
4.1 Design 104
4.2 Intervention 104
5.0 THERAPEUTIC/DIAGNOSTIC AGENTS 115
6.0 CRITERIA FOR SUBJECT ELIGIBILITY 137
6.1 Subject Inclusion Criteria 137
6.2 Subject Exclusion Criteria 137
7.0 RECRUITMENT PLAN 148
8.0 PRETREATMENT EVALUATION 148
9.0 TREATMENT/INTERVENTION PLAN 20
10.0 EVALUATION DURING TREATMENT/INTERVENTION 21
11.0 TOXICITIES/SIDE EFFECTS 18
12.0 CRITERIA FOR THERAPEUTIC RESPONSE/OUTCOME ASSESSMENT 31
13.0 CRITERIA FOR REMOVAL FROM STUDY 36
14.0 BIOSTATISTICS 37
15.0 RESEARCH PARTICIPANT REGISTRATION AND RANDOMIZATION PROCEDURES 40
15.1 Research Participant Registration 40
15.2 Randomization 41
16.0 DATA MANAGEMENT ISSUES 41
16.1 Quality Assurance 41
16.2 Data and Safety Monitoring 42
17.0 PROTECTION OF HUMAN SUBJECTS 43
17.1 Privacy 43
17.2 Serious Adverse Event (SAE) Reporting 43
17.2.1 415
18.0 INFORMED CONSENT PROCEDURES 45
19.0 REFERENCES 46
20.0 APPENDICES 448

1.0 PROTOCOL SUMMARY AND/OR SCHEMA

Phase II Trial of Single-Agent Nivolumab in Patients with Microsatellite Unstable/Mismatch Repair Deficient/Hypermutated Uterine Cancer

Recurrent or Persistent Endometrial Carcinoma, Uterine Carcinosarcoma, or Uterine Sarcoma

↓

Identify cases that are any of the following:

1. Microsatellite unstable (MSI-high or MSI-H)
2. Mismatch Repair Deficient (MMR-D)
3. Hypermutated (20 or more mutations on MSK-IMPACT)

↓

Nivolumab treatment: Nivolumab 480 mg IV once every 4 weeks.

Treatment will continue until time of disease progression or until development of unacceptable toxicity, whichever comes first. Patients who demonstrate radiologic progression by RECIST 1.1 criteria may be considered for continued therapy (but not primary efficacy analysis) if they are deemed to be clinically benefiting, according to predetermined permitted criteria (section 12.4)

CT or MRI CAP for assessment of disease response every 12 weeks
1 Cycle = 4 weeks (therefore there is 1 dose of nivolumab per treatment cycle)

2.0 OBJECTIVES AND SCIENTIFIC AIMS

2.1 Primary Objectives

2.1.1 To define the Progression-Free Survival rate at 24 weeks in patients with MSI-high, MMR-D, or hypermutated persistent or recurrent uterine cancer treated with single-agent nivolumab.

2.1.2 To define the proportion of patients who have objective tumor response (complete or partial) by RECIST 1.1 in patients with MSI-high, MMR-D, or hypermutated persistent or recurrent uterine cancer treated with single-agent nivolumab.

2.2 Secondary Objectives

2.2.1 To determine the progression-free survival rate by RECIST 1.1 and overall survival in this population of recurrent uterine cancer patients treated with nivolumab.

2.2.2 To determine the frequency and severity of adverse events associated with treatment with nivolumab as assessed by Common Terminology Criteria for Adverse Events (CTCAE) version 4.03.

2.2.3 To determine the duration of response and duration of disease control

2.3 Exploratory objectives

2.3.1 Correlate the somatic mutational burden* with clinical benefit from nivolumab, both overall response rate (ORR) and progression-free survival (PFS) rate at 24 weeks.

2.3.2 Correlate the somatic mutational burden identified by MSK-IMPACT* with the MSIsensor algorithm score*.

2.3.3 Correlate the MSIsensor score* with the MMR IHC status (immunohistochemical staining for mismatch repair enzyme proteins).

2.3.4. Correlate the pre-treatment immune phenotype (tumor infiltrating lymphocyte and PD-L1 status) with best overall response rate (ORR) and progression free survival rate at 24 weeks.

* Tissue will be analyzed for number of somatic mutations and MSIsensor score using MSK-IMPACT under the auspice of IRB #12-245. We will abstract the data collected under 12-245 from the EMR to answer questions in this study.

3.0 BACKGROUND AND RATIONALE

3.1 Rationale for Immunotherapy

Cancer of the uterine corpus is the most common gynecologic malignancy with an estimated 60,050 new cases in 2016 and 10,470 deaths in the United States [1]. The current standard of care for advanced carcinomas and carcinosarcomas of the uterus is combination chemotherapy with carboplatin and paclitaxel [2] and gemcitabine plus docetaxel for uterine leiomyosarcomas [3,4]. The overall response rate to these regimens is 57% and 27-36%, respectively, with complete responses being infrequent and all patients eventually dying from disease. More recent studies have used 6-month PFS as a criterion in the assessment of efficacy in advanced endometrial cancer; single-agent bevacizumab in this setting showed a PFS rate of 40.4% at 6 months and overall response rate of 13.5%[5]. There is a significant need for new therapeutic strategies that will provide long-lasting remissions in uterine cancer.

Nivolumab, a fully human, IgG4 (kappa) monoclonal antibody that binds to PD-1, has shown significant clinical activity as a single-agent in non-small cell lung cancer [6-8], melanoma [9], renal cell carcinoma [10-12], and non-Hodgkin lymphoma [13]. While it has been shown that host immune response plays a role in controlling malignant progression of epithelial ovarian cancer, thus suggesting immunotherapy as a rational therapeutic approach for this gynecologic malignancy[14], similar data in the setting of advanced uterine cancer has been lacking. In a study of 63 endometrial cancers, polymerase-e (POLE) mutated ultramutated tumors and microsatellite instability high (MSI-H) hypermutated tumors were found to be associated with high neoantigen loads, high number of tumor-infiltrating lymphocytes (TILs), and corresponding overexpression of PD-1 and PD-L1, suggesting POLE-mutant and MSI-H endometrial cancers as excellent candidates for PD-1 targeted immunotherapies [15]. This data is in keeping with previous data in melanoma demonstrating that an “inflammatory” gene signature corresponds to higher expression of PD-L1, which may serve as a mechanism of adaptive immune resistance and indicate a tumor that will respond to immunotherapeutic approaches [16].

In a study of mutational burden and mismatch repair status in colorectal cancer (CRC), 149 cases of CRC were analyzed by MSK-IMPACT, a targeted-exome capture assay of 341 cancer-associated genes. Ninety-eight percent of mismatch repair-proficient (MMR-P) cases had 16 or fewer somatic mutations detected with a median number of 6 mutations (range 0-16) whereas MMR-deficient (MMR-D) tumors had a median number of 49 somatic mutations (range 23-67) [17]. This study concluded that using a cutoff for mutational load, such as 20 somatic mutations in the MSK-IMPACT panel, could provide a highly sensitive and specific means of screening for MMR deficiency. While a straightforward approach such as this is appealing, a bioinformatic approach to interpreting mutational burden is likely to more accurately predict tumor MMR status or microsatellite instability (MSI). Such an approach to determining MSI status using paired tumor-normal sequence data generated by next generation sequencing has been developed [18] and provides a promising method of identifying MMR-D/MSI-high (MSI-H) cases that may be missed by conventional testing. Phase 2 trial results have demonstrated that clinical activity of the anti-programmed death 1 (anti-PD-1) immune checkpoint inhibitor pembrolizumab is enriched in MMR-D colorectal and other solid tumors [19]. These results, taken with the data from Howitt et al. summarized above, suggests that selecting patients with hypermutated, MMR-D, or MSI-H uterine cancers will enrich for response to anti-PD-1 therapy. While nivolumab has not been used in uterine cancer in a clinical trial setting, phase I trials in other solid tumors[20] and a case series using nivolumab in hypermutated uterine cancers[21] have shown the drug to be safe and have suggested efficacy in this setting, thus obviating the need for a phase I trial in this setting.

To determine how many patients with recurrent or persistent uterine cancer have a high burden of somatic mutations, we have analyzed 253 patients with uterine cancer by MSK-IMPACT to date under IRB protocol #12-245. These cases include 165 endometrial cancers and 88 uterine sarcomas, 23 (9.1%) of which have 20 or more somatic mutations (range 20-445; 22 endometrial cancers, 1 uterine sarcoma). We performed mutational signature decomposition analysis to determine the underlying mutational process in 17 of these hypermutated endometrial cancers. One outlier tumor with 445 somatic mutations was found to have a POLE *V411L* somatic hotspot mutation and had a predominantly POLE signature. The other 16 tumors, including 12 with MMR-deficiency by immunohistochemical (IHC) staining, 2 with unknown MMR status, and 2 that were MMR-proficient by IHC, had a predominant MSI-H mutational signature (Figure 1). Overall, mutational signature decomposition analysis from targeted sequencing data in hypermutated tumors revealed MMR deficiency even in cases where conventional testing with immunohistochemistry produced normal (MMR-proficient) results.

3.2 Nivolumab

Nivolumab (also referred to as BMS-936558 or MDX1106) is a human monoclonal antibody (HuMAb; immunoglobulin G4 [IgG4]-S228P) that targets the programmed death-1 (PD-1) cluster of differentiation 279 (CD279) cell surface membrane receptor. PD-1 is a negative regulatory molecule expressed by activated T and B lymphocytes. [20] Binding of PD-1 to its ligands, programmed death–ligands 1 (PD-L1) and 2 (PD-L2), results in the down-regulation of lymphocyte activation. Inhibition of the interaction between PD-1 and its ligands promotes immune responses and antigen-specific T-cell responses to both foreign antigens as well as self-antigens. Nivolumab is expressed in Chinese hamster ovary (CHO) cells and is produced using

standard mammalian cell cultivation and chromatographic purification technologies. The clinical study product is a sterile solution for parenteral administration.

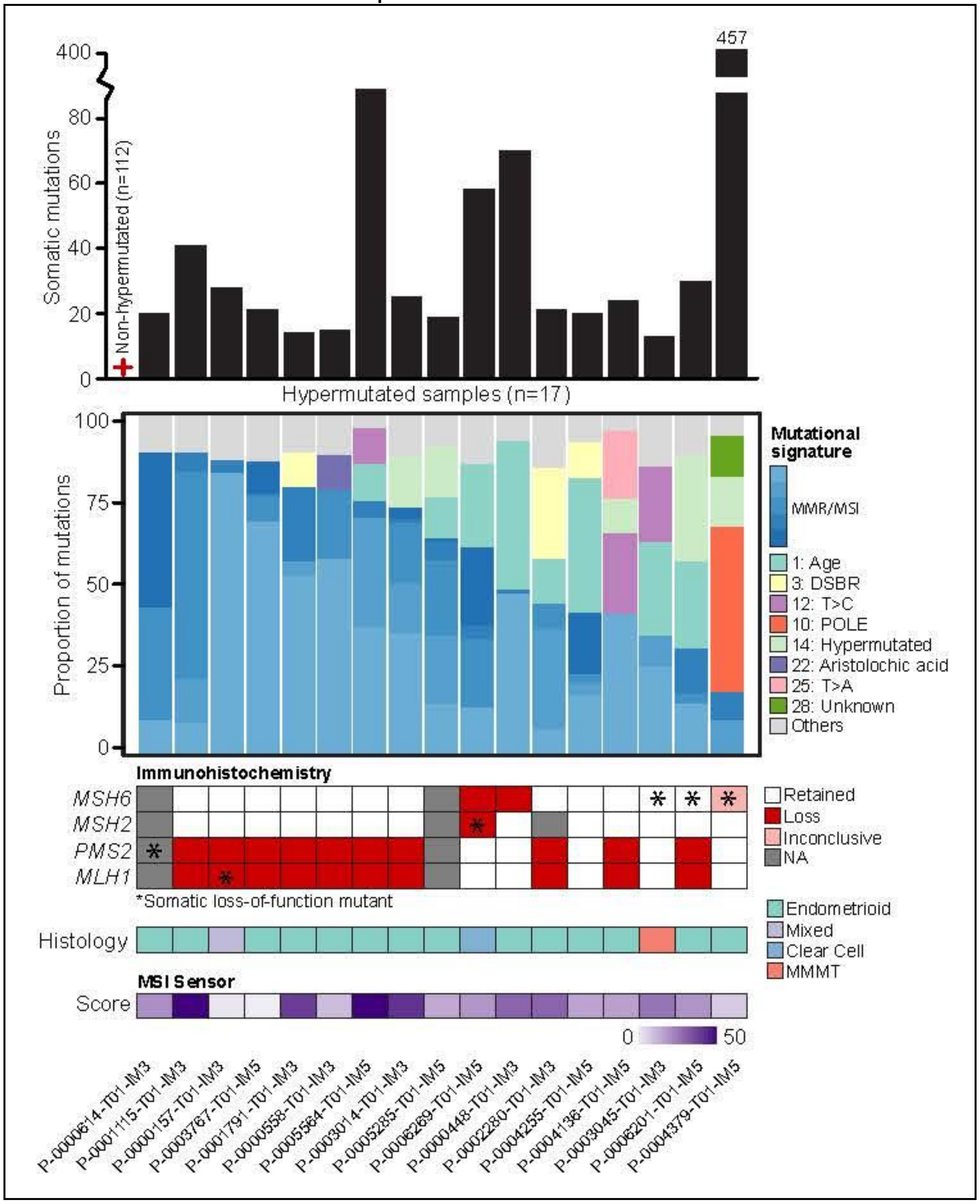

Figure 1. Origins of hypermutation. The count of somatic mutations from targeted sequencing is shown for 17 patients with apparent hypermutation. In red is the median and median absolute deviation of somatic mutations in 112 non-hypermutated endometrial tumors for comparison. Mutational signature decomposition analysis in the hypermutated tumors reveals the presence of an MMR/MSI dysfunction in all tumors at a frequency of 25% or greater. Other mutational signatures that reflected 10% of the total somatic mutations or more are also shown (see legend). Conventional immunohistochemical staining for MMR proteins indicates false negatives in tumors with evidence of MSI. Asterisks reflect the tumors in which somatic loss-of-function mutations were present in the genes indicated. Histologies of each of the tumors analyzed are also shown. MSI sensor for the indicated cases reflecting a complementary but orthogonal measure of MMR dysfunction in these tumors that is broadly concordant with mutational signature analysis, though the latter resolves equivocal cases.

OPDIVO™ (nivolumab) is approved for use in humans to treat several types of cancers, in the United States (US, Dec-2014), the European Union (EU, Jun-2015), Japan (Jul-2014), and several other countries.

3.2.1 Nonclinical Development of Nivolumab

Nivolumab has been shown to bind specifically to the human PD-1 receptor and not to related members of the CD28 family. [21] Nivolumab inhibits the interaction of PD-1 with its ligands, PD-L1 and PD-L2, resulting in enhanced T-cell proliferation and interferon-gamma (IFN-γ) release in vitro.[23,24] Nivolumab binds with high affinity to activated human T-cells expressing cell surface PD-1 and to cynomolgus monkey PD-1. [21] In a mixed lymphocyte reaction (MLR), nivolumab promoted a reproducible concentration-dependent enhancement of IFN- γ release. [23]

In intravenous (IV) repeat-dose toxicology studies in cynomolgus monkeys, nivolumab was well tolerated at doses up to 50 mg/kg, administered weekly for 5 weeks, and at doses up to 50 mg/kg, administered twice weekly for 27 doses. While nivolumab alone was well tolerated in cynomolgus monkeys, combination studies have highlighted the potential for enhanced toxicity when combined with other immunostimulatory agents. [21]

3.2.2 Clinical Development of Nivolumab

The PK, clinical activity, and safety of nivolumab have been assessed in subjects with non-small cell lung cancer (NSCLC), melanoma, and clear-cell renal cell carcinoma (RCC) in addition to other tumor types. Clinical activity and safety information derives primarily from that obtained from Phase 2/3 studies in subjects with advanced or metastatic squamous (SQ) and nonsquamous (NSQ) NSCLC, Phase 2/3 studies in subjects with unresectable or metastatic melanoma, and a Phase 2 study in subjects with advanced or metastatic clear-cell RCC [21]. Nivolumab is currently being investigated both as monotherapy and in combination with chemotherapy, targeted therapies, and other immunotherapies.

Nivolumab is approved in multiple countries including the US for treatment of previously treated, unresectable or metastatic melanoma and previously treated, metastatic squamous NSCLC, the EU for treatment of previously treated, unresectable or metastatic melanoma, and Japan for treatment of unresectable melanoma. See Appendix 1 for US Prescribing Information (USPI) and Appendix 2 for the EU Summary of Product Characteristics (SmPC).

<u>Clinical Pharmacokinetics</u>

The pharmacokinetics (PK) of nivolumab was studied in subjects over a dose range of 0.1 to 10 mg/kg administered as a single dose or as multiple doses of nivolumab every 2 or 3 weeks. The

geometric mean (% CV%) clearance (CL) was 9.5 mL/h (49.7%), geometric mean volume of distribution at steady state (Vss) was 8.0 L (30.4%), and geometric mean elimination half-life (t1/2) was 26.7 days (101%). Steady-state concentrations of nivolumab were reached by 12 weeks when administered at 3 mg/kg Q2W, and systemic accumulation was approximately 3-fold. The exposure to nivolumab increased dose proportionally over the dose range of 0.1 to 10 mg/kg administered every 2 weeks. The clearance of nivolumab increased with increasing body weight. The PPK analysis suggested that the following factors had no clinically important effect on the CL of nivolumab: age (29 to 87 years), gender, race, baseline LDH, PD-L1. A PPK analysis suggested no difference in CL of nivolumab based on age, gender, race, tumor type, baseline tumor size, and hepatic impairment.
Although ECOG status, baseline glomerular filtration rate (GFR), albumin, body weight, and mild hepatic impairment had an effect on nivolumab CL, the effect was not clinically meaningful. PPK and exposure response analyses have been performed to support use of 240 mg Q2W dosing of nivolumab in addition to the 3 mg/kg Q2W regimen. Using the PPK model, exposure of nivolumab at 240 mg flat dose was identical to a dose of 3 mg/kg for subjects weighing 80 kg, which was the approximate median body weight in nivolumab clinical trials. Across the various tumor types in the clinical program, nivolumab has been shown to be safe and well tolerated up to a dose level of 10 mg/kg, and the relationship between nivolumab exposure produced by 3 mg/kg and efficacy and safety has been found to be relatively flat. Given the similarity of nivolumab PK across tumor types and the similar exposures predicted following administration of 240 mg flat dose compared to 3 mg/kg, it is expected that the safety and efficacy profile of 240 mg nivolumab will be similar to that of 3 mg/kg nivolumab.
In addition, nivolumab 360 mg Q3W and nivolumab 480 mg Q4W are currently under investigation in monotherapy and combination oncology studies. The less frequent dosing regimens are designed to afford more convenience to the target patient populations and allow combination of nivolumab with other agents using alternative dosing regimens versus Q2W. These nivolumab dosing regimens were selected using PPK and exposure-response analyses modeling and simulation approaches such that they are predicted to provide approximately equivalent exposures (Cavgss) following administration of nivolumab 3 mg/kg Q2W. The model predicted that following administration of nivolumab 360 mg Q3W and 480 mg Q4W, Cavgss are expected to be similar to those following nivolumab 3 mg/kg or 240 mg Q2W, while Cminss are predicted to be 6% and ~16% lower, respectively, and are not considered to be clinically relevant. Following nivolumab 360 mg Q3W and 480 mg Q4W, Cmaxss are predicted to be approximately ~23% and ~43% greater, respectively, relative to that following nivolumab 3 mg/kg Q2W dosing; however, the range of nivolumab exposures (median and 90% prediction intervals) following administration of 360 mg Q3W and 480 mg Q4W regimens across the 35 to 160 kg weight range are predicted to be maintained well below the corresponding exposures observed with the well tolerated 10 mg/kg nivolumab Q2W dosing regimen, and are not considered to put participants at increased risk. Hence, a flat dose of 480 mg nivolumab is under investigation.

Clinical Efficacy

Nivolumab has demonstrated durable responses exceeding 6 months as monotherapy and in combination with ipilimumab in several tumor types, including NSCLC [6-8], melanoma [9], RCC [10-12], and some lymphomas [13]. In confirmatory trials, nivolumab as monotherapy demonstrated a statistically significant improvement in OS as compared with the current standard of care in subjects with advanced or metastatic NSCLC [6-8] and in subjects with unresectable or metastatic melanoma [9].

Clinical Safety

The overall safety experience with nivolumab, as a monotherapy or in combination with other therapeutics, is based on experience in approximately 8,600 subjects treated to date.

For monotherapy, the safety profile is similar across tumor types. There is no pattern in the incidence, severity, or causality of AEs to nivolumab dose level. In Phase 3 controlled studies, the safety profile of nivolumab monotherapy is acceptable in the context of the observed clinical efficacy, and manageable using established safety guidelines. Clinically relevant AEs typical of stimulation of the immune system were infrequent and manageable by delaying or stopping nivolumab treatment and timely immunosuppressive therapy or other supportive care.

3.2.3 Shorter Infusion Duration

Administration of nivolumab using a 30-minute infusion time has been evaluated in subjects with cancer. Previous clinical studies of nivolumab monotherapy for the treatment of cancer have used a 60-minute infusion duration wherein nivolumab has been safely administered up to 10 mg/kg over long treatment periods. Infusion reactions including high-grade hypersensitivity reactions have been uncommon across the nivolumab clinical program. In CA209010, a dose association was observed for infusion site reactions and hypersensitivity reactions (1.7% at 0.3 mg/kg, 3.7% at 2 mg/kg and 18.5% at 10 mg/kg). All the events were Grade 1-2 and were manageable. An infusion duration of 30 minutes for 3 mg/kg nivolumab (30% of the dose provided at 10 mg/kg) is not expected to present any safety concerns compared to the prior experience at 10 mg/kg nivolumab dose infused over a 60-minute duration. The safety of nivolumab 3 mg/kg administered as a 30-min infusion was assessed in CA209153 in patients (n=322) with previously treated advanced NSCLC (see Section 5.5.1.2). Overall, there were no clinically meaningful differences in the frequency of hypersensitivity/infusion-related reactions (of any cause or treatment-related) in cancer patients administered nivolumab over a 30 min infusion compared with that reported for patients with the 60 min infusion. Thus, it was shown that nivolumab can be safely infused over 30 min in subjects with cancer.

3.3 Translational Science Background

3.3.1 Mutational Burden and MSIsensor (Exploratory Biomarkers)

As detailed above, previous studies have shown that a count of the absolute number of somatic mutations can predict which tumors are MSI-H or MMR-D [17], and that the clinical benefit of PD-1/PD-L1 pathway blockade is enriched in MMR-D tumors [19]. In this trial, pre-treatment matched pairs of tumor and normal DNA from formalin-fixed paraffin embedded (FFPE) tissue will be evaluated by MSK-IMPACT under IRB protocol #12-245. MSK-IMPACT is a next generation sequencing assay of 410 cancer-associated genes performed in a CLIA-compliant laboratory. It identifies somatic point mutations, small insertions and deletions, structural rearrangements, and DNA copy number alterations in the tumors as compared to matched normal samples. Treating the number of somatic mutations in each patient's tumor as a continuous variable, we will seek to correlate the patient's tumor's mutational burden with clinical benefit from nivolumab, both overall response rate (ORR) and progression-free survival (PFS) at 24 weeks.

The MSIsensor algorithm is a method for assessing MSI, yielding a "high" or "low" result. We aim to correlate the MSK-IMPACT somatic mutation number with the MSIsensor score. The MSIsensor algorithm may also be correlated with the MMR IHC results from the tumor.
We will also explore the correlation of pre-treatment immune phenotype (tumor infiltrating lymphocyte and PD-L1 status,) with best overall response rate (ORR) and progression free survival rate at 24 weeks.

All patients on this study will also be consented to IRB protocol #12-245 in order to have MSK-IMPACT sequencing performed. All of the questions we seek to answer using MSK-IMPACT results in this protocol will use data abstracted from the EMR.

3.3.2 Analyzing the Inflammatory Phenotype in the Tumor Microenvironment (Exploratory Biomarker)

Immune infiltration of tumors has been demonstrated in multiple studies to be both prognostic and predictive of response to immunotherapies, with tumors exhibiting inflamed phenotypes being most responsive. Assessment of the immune subsets in the tumors will help to establish predictive markers on the basis of pre-treatment phenotype as well to determine whether clinical efficacy correlates with the degree of infiltration of specific immune cell subsets. In addition, both PD-L1 and indoleamine-2,3-dioxygenase (IDO) have been demonstrated to be up-regulated in tumors in response to immune infiltration and may serve as negative feedback mechanisms [24]. As such, pre-treatment FFPE will be evaluated for the presence of TILs and immune marker PD-L1. Evaluation of TILs and PD-L1 will be performed in the laboratory of Dr. Jedd Wolchok in collaboration with Dr. Dmitriy Zamarin. See appendix 4.

3.4 Inclusion of Women and Minorities

Participating institutions will not exclude potential subjects from participating in this or any study solely on the basis of ethnic origin or socioeconomic status. Every attempt will be made to enter all eligible patients into this protocol and therefore address the study objectives in a patient population representative of the entire uterine cancer population treated by participating institutions.

4.0 OVERVIEW OF STUDY DESIGN/INTERVENTION

4.1 Design

This is an MSKCC investigator-initiated, single-center, non-randomized, open-label, phase 2 study to evaluate the activity of nivolumab in patients with recurrent or persistent hypermutated, MMR-D, or MSI-H uterine cancer. Activity of nivolumab will be assessed by determining the percentage of patients who remain progression-free at 24 weeks (primary endpoint) or who have objective RECIST response (co-primary endpoint). The safety and tolerability of single-agent nivolumab in this population will also be evaluated.

Each cycle will be 28 days (4 weeks) in duration. Patients will receive study treatment with nivolumab until disease progression, development of intolerable toxicity, elective withdrawal from the study, study completion, or study termination. Patients who demonstrate radiologic progression by RECIST criteria may be considered for continued therapy (but not primary efficacy analysis) if they are deemed to be clinically benefiting, according to predetermined permitted criteria (section 12.4).

Safety will be evaluated through the monitoring of all serious and non-serious AEs and irAE's, graded according to the current version of National Cancer Institute's Common Terminology Criteria for Adverse Events (CTCAE v. 4.03). AEs will be collected from the time of study enrollment until 30 days after last dose of study drug dose.

4.2 Intervention

Eligible patients will undergo screening and baseline procedures per the Study Schedule. Study inclusion and exclusion criteria will be applied per section 6.0. Enrolled patients will receive

nivolumab 480 mg IV every 4 weeks. Each cycle will be 28 days in duration. Patients will receive study treatment until disease progression, intolerable toxicity, elective withdrawal from the study, study completion, or study termination.

Efficacy assessments will be performed at the initiation of study treatment and then every 12 weeks (approximately every 3 cycles) until radiologic and/or clinical disease progression. Patients who discontinue treatment for reasons other than progression will continue efficacy assessments until progression is demonstrated. Patients who demonstrate radiologic progression by RECIST criteria may be considered for continued therapy (but not primary efficacy analysis) if they are deemed to be clinically benefiting, according to pre-determined permitted criteria (section 12.4).

Nivolumab 480 mg will be infused IV over 30 minute every 4 weeks. (A treatment window of up to 3 days from the treatment due date will be permitted, provided there are at least 10 days between treatment infusions.)

5.0 THERAPEUTIC/DIAGNOSTIC AGENTS

5.1 Nivolumab

5.1.1 Physical and Chemical Properties

Nivolumab was selected for dosage form development and is also referred to as BMS-936558-01 or BMS-936558. Nivolumab is a soluble protein consisting of 4 polypeptide chains, which include 2 identical heavy chains and 2 identical light chains. The physical and chemical properties of nivolumab are provided in Table 5.1-1.

Table 5.1-1: Physical and Chemical Properties

BMS Number	BMS-936558-01
Other Names	Nivolumab, BMS-936558, MDX1106, ONO-4538, anti-PD-1
Molecular Weight	146,221 daltons (143,619.17 daltions, protein portion)
Appearance	Clear to opalescent, colorless to pale yellow liquid, light (few) particulates may be present
Solution pH	5.5 to 6.5

5.1.2 Pharmaceutical Properties and Formulation

<u>Description of the Dosage Form</u>
Nivolumab Injection, 100 mg/10 mL (10 mg/mL), Nivolumab Injection, 40 mg/4 mL (10 mg/mL), and Placebo for Nivolumab Injection, 100 mg/Vial (10 mL label fill) Nivolumab Injection, 100 mg/10 mL (10 mg/mL) or 40 mg/4 mL (10 mg/mL), is a clear to opalescent, colorless to pale yellow liquid, which may contain light (few) particulates. The drug product is a sterile, non-pyrogenic, single-use, isotonic aqueous solution formulated at 10 mg/mL in sodium citrate, sodium chloride, mannitol, diethylenetriaminepentacetic acid (pentetic acid), and polysorbate 80 (Tween 80), pH 6.0 and includes an overfill to account for vial, needle, and syringe holdup. It is supplied in 10-cc Type I flint glass vials, stoppered with butyl rubber stoppers and sealed with aluminum seals. The only difference between the two drug product presentations is the vial fill volume.

<u>Drug Product Preparation</u>
Nivolumab Injection, 100 mg/10 mL (10 mg/mL), Nivolumab Injection, 40 mg/4 mL (10 mg/mL), and Placebo for Nivolumab Injection, 100 mg/Vial (10 mL label fill)
Nivolumab injection is to be administered as an IV infusion through a 0.2-micron to 1.2-micron pore size, low-protein binding in-line filter at the protocol-specified doses and infusion times. It is

not to be administered as an IV push or bolus injection. Nivolumab injection can be infused undiluted (10 mg/mL) or diluted with 0.9% Sodium Chloride Injection, USP or 5% Dextrose Injection, USP to protein concentrations as low as 0.35 mg/mL. During drug product preparation and handling, vigorous mixing or shaking is to be avoided. Instructions for dilution and infusion of nivolumab injection may be provided in the clinical protocol, pharmacy binder, or pharmacy reference sheet. Care must be taken to assure sterility of the prepared solution as the product does not contain any antimicrobial preservative or bacteriostatic agent. No incompatibilities between nivolumab and polyvinyl chloride (PVC) and non-PVC/non-DEHP (di(2-ethylhexyl)phthalate) containers/IV components or glass bottles have been observed.

PRODUCT INFORMATION TABLE: Please see Appendix 1: US Prescribing Information

Product Description:(Other names = MDX-1106, ONO-4538, anti-PD-1

Product Description and Dosage Form	Potency	Primary Packaging (Volume)/ Label Type	Secondary Packaging (Qty) /Label Type	Appearance	Storage Conditions (per label)
Nivolumab (BMS-936558-01)* Injection drug product is a sterile, non-pyrogenic, single-use, isotonic aqueous solution formulated at 10 mg/mL	100 mg/Vial (10 mg/mL).	Carton of 5 or 10 vials	10-cc Type 1 flint glass vials stoppered with butyl stoppers and sealed with aluminum seals.	Clear to opalescent, colorless to pale yellow liquid. May contain particles	**BMS-936558-01 Injection** must be stored at 2 to 8 degrees C (36 to 46 degrees F) and protected from light and freezing

*Nivolumab may be labeled as BMS-936558-01 Solution for Injection

If stored in a glass front refrigerator, vials should be stored in the carton. Recommended safety measures for preparation and handling of nivolumab include laboratory coats and gloves.
For additional details on prepared drug storage and use time of nivolumab under room temperature/light and refrigeration, please refer to the BMS-936558 (nivolumab) Investigator Brochure section for “Recommended Storage and Use Conditions”
Recommended Storage and Use Conditions
Nivolumab Injection, 100 mg/10 mL (10 mg/mL) and 40 mg/4mL (10 mg/mL)
Vials of nivolumab injection must be stored at 2º to 8ºC (36º to 46ºF) and protected from light and freezing.

Undiluted Nivolumab Injection and Diluted Nivolumab Injection in the IV Container
The administration of nivolumab infusion must be completed within 24 hours of preparation. If not used immediately, the infusion solution may be stored under refrigeration conditions (2°to 8°C, 36°to 46°F) for up to 24 hours, and a maximum of 8 hours of the total 24 hours can be at room

temperature (20° to 25°C, 68° to 77°F) and room light. The maximum 8-hour period under room temperature and room light conditions includes the product administration period.

Placebo for Nivolumab Injection, 100 mg/Vial (10 mL label fill)
The same storage and use conditions for the active drug product also apply to the placebo for nivolumab injection.

6.0 CRITERIA FOR SUBJECT ELIGIBILITY

6.1 Subject Inclusion Criteria

1. Histologically confirmed diagnosis of metastatic or recurrent uterine cancer (endometrial carcinoma, carcinosarcoma, clear cell carcinoma, leiomyosarcoma, undifferentiated sarcoma, high grade endometrial stromal sarcoma) by Memorial Sloan Kettering Cancer Center. Carcinosarcomas, endometrioid and clear cell carcinomas that appears to have arisen in the ovary/fallopian tube or peritoneum are also eligible. Recurrence should not be amenable to curative approaches such as surgical resection or chemoradiotherapy.
2. Tumor is confirmed to be one of the following: 1. MSI-high, or 2. MMR-deficient, or 3. Hypermutated defined as ≥20 somatic mutations in the tumor by MSK-IMPACT
3. One or more prior lines of cytotoxic treatment for advanced disease (prior hormonal therapy is not considered to count as prior lines of therapy)
4. Measurable disease by RECIST 1.1 criteria
5. No known CNS metastases
6. ECOG Performance status 0-1
7. WBC ≥ 2000/uL, ANC ≥ 1500/uL, PLT ≥ 100,000/uL, HGB ≥ 8 g/dL
8. Serum creatinine ≤ 1.5 x ULN or creatinine clearance of ≥ 40mL/min by Cockroft-Gault formula
9. AST (SGOT) and ALT (SGPT) ≤ 3 x ULN
10. Total bilirubin ≤ 1.5 x ULN, except subjects with Gilbert's syndrome who can have total bilirubin ≤ 3.0 mg/dL
11. Able to sign voluntary written informed consent
12. Female, 18 years of age or older
13. Available archival tumor tissue or patient is willing to undergo new biopsy
14. Premenopausal women of child bearing potential must have a normal urine or serum beta-HCG prior to enrollment, and must agree to use effective contraception during treatment with nivolumab and for at least 5 months following the last dose of nivolumab.

6.2 Subject Exclusion Criteria

1. Disease eligible for potentially curative treatment with standard chemotherapy, surgical resection, or chemoradiotherapy.
2. Known or suspected autoimmune disease, except for subjects with vitiligo, diabetes mellitus, resolved childhood asthma/atopy, residual hypothyroidism due to an autoimmune immune condition only requiring thyroid hormone replacement, psoriasis not requiring systemic treatment, or conditions not expected to recur in the absence of an external trigger.
3. Serious uncontrolled medical disorder or active infection which would impair the ability of the subject to receive protocol therapy or whose control would be jeopardized by protocol therapy
4. History of bowel obstruction, refractory ascites, or bowel perforation due to advanced disease within the past 3 months from start of study treatment.

5. Prior therapy with an anti-PD-1, anti-PD-L1, anti-PD-L2, anti-CD137, anti-CTLA-4 antibody or any other antibody or drug specifically targeting T cell co-stimulation or immune checkpoint pathways
6. Patients who have a condition that requires systemic treatment with either corticosteroids within 7 days of enrollment (systemic corticosteroid therapy is defined as >10 mg daily prednisone or its equivalent); or who require other immunosuppressive medications within 14 days of study drug administration. Inhaled or topical steroids and adrenal replacement doses > 10 mg daily prednisone equivalents are permitted in the absence of active autoimmune disease.
7. Prior history of malignancy or a concurrent malignancy, with the exception of cutaneous basal cell carcinoma or squamous cell carcinoma, superficial bladder cancer, or in situ carcinoma of the uterine cervix, prostate, or breast, unless a complete remission was achieved at least 3 years prior to study entry and no additional therapy is required or anticipated to be required during the study period
8. Breastfeeding women, pregnant women
9. Prisoners or subjects who are involuntarily incarcerated
10. Subjects who are compulsorily detained for treatment of either a psychiatric or physical illness
11. Positive test for hepatitis B virus surface antigen (HBV sAg) or hepatitis C virus ribonucleic acid (HCV antibody) indicating acute or chronic infection (if patient has documented Hepatitis B and C from within 6 months of enrollment, these tests do not need to be repeated.)
12. Known history of testing positive for human immunodeficiency virus (HIV) or known acquired immunodeficiency syndrome (AIDS)
13. Known allergy or Adverse Drug Reaction to nivolumab, or a history of allergy to study drug components.

7.0 RECRUITMENT PLAN

Potential research subjects will be identified by a member of the patient's treatment team, the protocol investigator or research team within the Gynecologic Medical Oncology Group at Memorial Sloan Kettering Cancer Center (MSKCC). Patient recruitment will occur in the Gynecologic Medical Oncology clinics at MSKCC. The investigator will discuss the study with suitable participants, and should the patient consent to proceed with protocol therapy, will enroll their patients in the research study. Approximately 1-2 patients will accrue onto this study per month. All participants will be women.

8.0 PRETREATMENT EVALUATION

Within 28 days prior to treatment start:

- Written informed consent
- History and Physical examination
- Review of concomitant medications
- ECOG Performance status
- Toxicity assessment
- 12-lead ECG
- Radiographic tumor measurements (CT or MRI C/A/P)
- Request fresh or archival tumor (10-20 FFPE slides or tissue block).

- Consent patient to enrollment on 12-245 for MSK-IMPACT testing (if patient not previously tested)

Within 14 days prior to treatment start:

- Complete Blood Count (CBC) with differential
- Comprehensive metabolic profile (Na, K, Cl, bicarbonate, BUN, creatinine, glucose, Ca, Mg, phosphate, total protein, albumin, ALT, AST, total bilirubin, alkaline phosphatase)
- LDH
- Amylase and lipase
- Thyroid function tests (TSH, free T3 and free T4)
- CA125 (only for patients with endometrial carcinoma or carcinosarcoma)
- Urinalysis
- Vital signs (temperature, blood pressure, pulse rate, and respiratory rate) will be measured on study days noted in the Schedule of Assessments.
- Serum or urine beta-HCG (pregnancy test) ONLY for women of child bearing potential

The following clinical laboratory tests will be performed (see the Schedule of Assessments, Section 10.0 for the time points of each test):

- Coagulation parameters: Activated partial thromboplastin time (PTT) and International normalized ratio (INR) to be assessed at baseline and as clinically indicated
- Thyroid Stimulating Hormone
    - free T3 and free T4, and TSH
    - repeat thyroid function tests approximately every 8 weeks (every other cycle)
- Other laboratory tests
    - Hepatitis B and C testing (HBV sAg and HCV Ab or HCV RNA) if these were not performed within 6 months of enrollment.

Table 1. Hematology Laboratory Tests

Basophils	Mean corpuscular volume
Eosinophils	Monocytes
Hematocrit	Neutrophils
Hemoglobin	Platelet count
Lymphocytes	Red blood cell count
Mean corpuscular hemoglobin	Total white cell count
Mean corpuscular hemoglobin concentration	

Table 2. Clinical chemistry (Serum or Plasma) Laboratory Tests

Albumin	Glucose
Alkaline phosphatase	Lactate dehydrogenase
Alanine aminotransferase	Lipase
Amylase	Magnesium
Aspartate aminotransferase	Potassium
Bicarbonate	Phosphate
CA-125	Sodium
Calcium	Thyroid stimulating hormone
Chloride	Total bilirubin[a]
Creatinine	Total protein
Free T3	Urea or blood urea nitrogen, depending on local practice
Free T4	

[a] If Total bilirubin is ≥ 1.5 x ULN and < 3xULN (and no evidence of Gilbert's syndrome) then fractionate into direct and indirect bilirubin to consider Gilbert's syndrome

Table 3. Urinalysis Tests[a]

Bilirubin	pH
Blood	Protein
Glucose	Specific gravity
Ketones	Colour and appearance

[a] Microscopy should be used as appropriate to investigate white blood cells and use the high power field for red blood cells

9.0 TREATMENT/INTERVENTION PLAN

All subjects will receive single-agent nivolumab. Each cycle will be 4 weeks in duration. Patients will receive study treatment until disease progression, intolerable toxicity, elective withdrawal from the study, study completion, or study termination. Patients who demonstrate radiologic progression by RECIST criteria may be considered for continued therapy (but not primary efficacy analysis) if they are deemed to be clinically benefiting, according to predetermined permitted criteria (section 12.4)

Nivolumab 480 mg will be infused IV over 30 minutes every 4 weeks. A treatment window of up to 3 days from the treatment due date will be permitted.

Efficacy assessments will be performed every 12 weeks (3 cycles) from initiation of study treatment until disease progression. Patients who discontinue treatment for reasons other than progression will continue efficacy assessments until progression.

Safety will be evaluated in this study through the monitoring of all serious and non-serious AEs and irAEs, graded according to the current version of the National Cancer Institute. AEs will be collected from the time of study enrollment until 30 days after last dose of study drug delivered.

Pre- and post-treatment blood and serum samples, as well as fresh or archival tumor tissue (when available) will be collected for biomarker analysis (as per Appendix 4).

10.0 EVALUATION DURING TREATMENT/INTERVENTION

All assessments to be performed pre-infusion unless stated otherwise. A Cycle is 28 Days.

**Parameter**	**Screening**		**Cycle 1**		**Cycle 2 onward**	**End of Treatment** [h]	**Follow-up** [h]
**Day**	**28 days before** [a]	**Within 14 days before**	**Day 1**	**Day 15**	**Day 1**		
Consent	x						
Medical History	x					x	
Concomitant Medications	x		x	x	x	x	
Request Archival tumor tissue	x						
Physical Examination	x		x		x		
ECOG	x		x		x		
Weight	x		x		x		
Vital Signs (BP, HR, and temperature), height	x		x	x	x		
Adverse Event Assessment	x		x		x	x	Report AEs until 4 weeks after last dose study drug
CBC		x	x	x	x	x	
CMP (AST, ALT, alkaline phosphatase, calcium, Na, K, Cl, glucose		x	x	x	x	x	
Serum or urine pregnancy test		x					

ONLY for WOCBP							
Magnesium		x	x		x	x	
Phosphate		x	x		x	x	
LDH		x	x		x	x	
Amylase		x	x		x	x	
Lipase		x	x		x	x	
TSH [f]		x	Every 8 weeks			x	
Free T3 [f]		x	Every 8 weeks			x	
Free T4 [f]		x	Every 8 weeks			x	
CA-125[b]		x	x		x	x	
PT/INR		x	As clinically indicated				
Urinalysis		x	As clinically indicated				
Research bloods [c]		x	x	x	Every 12 weeks		
12- Lead ECG	x						
Hepatitis B,C screening	x[g]						
Nivolumab [d,e]			x		x		
Radiographic disease assessment by CT or MRI CAP [d]	x		Every 12 weeks				

[a] If screening laboratory assessments are performed within 3 days prior to Day 1 they do not need to be repeated at Cycle 1 Day 1.
[b] CA-125 only for endometrial carcinoma, carcinosarcoma or clear cell carcinoma, at start of each cycle
[c] Research bloods will consist of 4 CPT tubes and 2 Streck tubes. Research bloods will be drawn at following time points: baseline, cycle 1 day 15, cycle 2 day 1, cycle 3 day 1 and every 12 weeks until progression. The baseline draw may be up to 14 days prior to Cycle 1 Day 1. Please see IMF requisition form and Appendix 4 for details of blood processing and delivery.
[d] ±3 day window for all parameters except for Radiographic disease assessment which has a ±7 day window as clinically indicated.
[e] ±3 day window for nivolumab treatment days
[f] Thyroid function tests (TSH, Free T3, Free T4) will be drawn at baseline and then every 8 weeks (every other cycle) starting Cycle 2
[g] Hepatitis screening for Hep B and C must be negative for patient to be eligible. Patients with a positive test for hepatitis B virus surface antigen (HBV sAg) or hepatitis C virus ribonucleic acid (HCV antibody) indicating acute or chronic infection will not be eligible. If patient has documented Hepatitis B and C screening tests from within 6 months of enrollment, these tests do not need to be repeated.
WOCBP = women of child-bearing potential
[h] ±7 day window for end of treatment and follow –up.

11.0 TOXICITIES/SIDE EFFECTS

11.1 Nivolumab

11.1.1 Summary of Adverse Events

A cumulative review of available data from completed and ongoing studies was performed 21. There have been some reports of adverse events with BMS-936558 in clinical studies. Based on the nature and frequency of the observed events, the events identified as ADRs are listed by system organ class in Table 11.0-1. As there is potential for hepatic toxicity with nivolumab, drugs with a predisposition to hepatoxicity should be used with caution in patients treated with nivolumab-containing regimen.

ADRs reported in clinical studies of nivolumab that are considered by the Sponsor to be causally related to nivolumab are listed by system organ class and frequency. The frequency of ADRs is defined using the following convention: very common (≥1/10); common (≥1/100 to < 1/10); uncommon (≥1/1,000 to < 1/100); rare (≥1/10,000 to < 1/1,000); and very rare (< 1/10,000).

As of 29-April-2015, the following ADRs have been reported in clinical studies where nivolumab was given as monotherapy (Table 11.0-1).

Table 11.0-1: Adverse Drug Reactions in Subjects Treated with Nivolumab Monotherapy

System Organ Class	Preferred Term	Overall Frequency[a]	*At least one event with outcome:*		
			**Serious**	**Life Threatening**	**Fatal**
**Cardiac disorders**	Arrhythmia [b] (including ventricular arrhythmia)	Uncommon	X		X
	Tachycardia [b]	Uncommon	X		
**Endocrine disorders**	Hypothyroidism	Common	X		
	Hyperthyroidism	Common	X		
	Hyperglycemia	Common	X	X	
	Adrenal insufficiency	Uncommon	X		
	Hypopituitarism [c]	Uncommon	X		
	Hypophysitis	Uncommon	X		
	(Autoimmune) Thyroiditis [c]	Uncommon	X		
	Diabetic ketoacidosis	Uncommon	X	X	
	Diabetes mellitus [c]	Uncommon	X	X	
**Eye disorders**	Uveitis (including iridocyclitis)	Uncommon	X		
**Gastrointestinal disorders**	Diarrhea	Very common	X		
	Nausea	Very common	X		
	(Autoimmune) Colitis	Common	X		X
	Stomatitis [c] (including mucosal inflammation/ ulceration, mouth ulceration)	Common	X		
	Vomiting	Common	X		
	Abdominal pain	Common	X		X
	Constipation [c]	Common	X		
	Dry mouth [b]	Common			
	Pancreatitis	Uncommon	X		
	Duodenal ulcer [b]	Uncommon	X		
**General disorders and administration site conditions**	Fatigue (including asthenia)	Very common	X		X
	Pyrexia	Common	X		
	Edema (including peripheral edema, generalized edema, orbital edema, swelling face)	Common	X		
**Hepatobiliary disorders**	(Autoimmune) hepatitis	Common	X	X	
**Immune system disorders**	Infusion related reaction	Common	X	X	
	Anaphylactic reaction	Uncommon	X	X	

	Hypersensitivity	Uncommon	X	X	
**Infections and infestations**	Upper respiratory tract infection [b]	Common	X		
	Bronchitis [b]	Uncommon	X		
**Investigations**	Lipase increased	Common	X		
	Amylase increased	Common	X		
	Alanine aminotransferase increased	Common	X	X	
	Aspartate aminotransferase increased	Common	X	X	
	Blood bilirubin increased	Common	X		X
**Metabolism and nutrition disorders**	Hyponatremia [c]	Common	X		
	Decreased appetite	Common	X		X
**Musculoskeletal and connective tissue disorders**	Musculoskeletal pain [b,d]	Common	X		
	Arthralgia (including arthritis, polyarthritis, and osteoarthritis)	Common	X		
	Polymyalgia rheumatica [b]	Uncommon	X		
**Neoplasms benign, malignant, and unspecified (including cysts and polyps)**	Histiocytic necrotizing lymphadenitis [b] (Kikuchi lymphadenitis)	Uncommon			
**Nervous system disorders**	Neuropathy peripheral [e]	Common	X		
	Headache	Common	X		
	Dizziness [b] (including vertigo, vertigo positional)	Common	X		
	Guillain-Barre syndrome	Uncommon	X		
	Demyelination	Uncommon	X		
	Myasthenic syndrome [b]	Uncommon	X		
	Autoimmune neuropathy (including facial and abducens nerve paresis)	Uncommon	X		
	Encephalitis	Uncommon	X	X	X
**Renal and urinary disorders**	Nephritis	Common	X		
	Renal failure (including acute kidney injury)	Uncommon	X	X	X
	Tubulointerstitial nephritis	Uncommon	X		
**Respiratory, thoracic, and mediastinal disorders**	Pneumonitis (including interstitial lung disease)	Common	X	X	X
	Dyspnea	Common	X	X	X

	Cough	Common	X		
	Lung infiltration	Uncommon	X		
**Skin and subcutaneous tissue disorders**	Rash [f]	Very common	X		
	Pruritus	Very common	X		
	Vitiligo [b]	Common	X		
	Dry skin [b]	Common			
	Erythema [b]	Common			
	Alopecia [b]	Common			
	Erythema multiforme	Uncommon	X		
	Psoriasis [b]	Uncommon	X		
	Rosacea [b]	Uncommon			
	Toxic epidermal necrolysis [b] (including Stevens Johnson syndrome)	Rare	X		X
	Urticaria [b]	Uncommon			
**Vascular disorders**	Hypertension [b]	Common	X		
	Vasculitis [b]	Uncommon			

a Overall frequency includes reported serious and non-serious ADRs
b New ADR added in this IB
c New ADR added to monotherapy table (previously reported in combination therapy
d Musculoskeletal pain is a composite term which includes back pain, bone pain, musculoskeletal chest pain, musculoskeletal discomfort, myalgia, neck pain, pain in extremity, pain in jaw, and spinal pain.
e Neuropathy peripheral is a composite term which includes burning sensation, peripheral motor neuropathy, Polyneuropathy, and peripheral sensory neuropathy.
f Rash is a composite term which includes maculo-papular rash, rash generalised, rash erythematous, rash pruritic, rash follicular, rash macular, rash papular, rash pustular, rash vesicular, dermatitis, dermatitis acneiform, dermatitis allergic, dermatitis exfoliative, and drug eruption.

There have been no additional ADRs identified from postmarketing experience with BMS-936558.

11.1.2 Immune-Related Adverse Event (irAEs) General Definition, Monitoring, and Management

Guidelines for the management of adverse events and toxicities are detailed in Appendix 3.

For the purposes of this protocol, an immune-related adverse reaction irAE is defined as an adverse reaction of unknown etiology associated with drug exposure and consistent with an immune phenomenon. Efforts should be made to rule out neoplastic, infectious, metabolic, toxin or other etiologic causes prior to labeling an event an irAEs. Serologic, immunologic, and histologic (biopsy) data should be used to support the diagnosis of an immune-related toxicity. Suspected immune-related adverse reactions must be documented on an AE or SAE form.

Overall, immune-related AEs commonly start within 3 to 10 weeks from initiation of therapy and are in most cases successfully managed by delaying doses, discontinuing dosing, and/or through administering symptomatic or immunosuppressive therapy, including corticosteroids, as mentioned below. Immune-related AEs generally resolved within days to weeks in the majority of subjects.

Patients should be informed of and carefully monitored for evidence of clinically significant systemic immune-mediated adverse reactions (e.g., systemic lupus erythematosus-like diseases)

or organ-specific immune-mediated adverse reaction (e.g., rash, colitis, uveitis, hepatitis or thyroid disease). If an immune-mediated adverse reaction is noted, appropriate work-up (including biopsy if possible) should be performed, and steroid therapy may be considered if clinically necessary.

It is unknown if systemic corticosteroid therapy has an attenuating effect on nivolumab activity. However, clinical anti-tumor responses have been maintained in patients treated with corticosteroids and discontinued from ipilimumab. If utilized, corticosteroid therapy should be individualized for each patient. For example, prior experience suggests that colitis manifested as ≥ grade 3 diarrhea requires corticosteroid treatment. If an irAE is documented, in general, delay protocol therapy and initiate corticosteroids earlier to obtain resolution with the possibility for resuming protocol therapy rather than waiting for higher grade events.

Potential safety concerns and recommended management guidelines regarding pulmonary toxicities, GI toxicities, hepatotoxicities, endocrinopathies, dermatologic toxicities, and other toxicities of concern are summarized below and described in detail in Appendix 3.

Pulmonary Adverse Events

Pulmonary AEs have been observed following treatment with nivolumab and have occurred after a single dose and after as many as 48 treatments. The frequency of pulmonary AEs may be greater with nivolumab combination therapies than with nivolumab monotherapy. The majority of cases reported were Grade 1 or 2, and subjects presented with either asymptomatic radiographic changes (eg, focal ground glass opacities and patchy infiltrates) or with symptoms of dyspnea, cough, or fever. Subjects with reported Grade 3 or 4 pulmonary AEs were noted to have more severe symptoms, more extensive radiographic findings, and hypoxia. Pulmonary AEs have been reported in subjects with a variety of tumor types; however, there have been numerically more cases in subjects with NSCLC. It is not clear whether the underlying NSCLC is a distinct risk factor, or if subjects with NSCLC are more likely to develop radiographic changes and symptoms for which it is difficult to distinguish between nivolumab related and unrelated causes. At this time, no other underlying risk factor, including prior radiotherapy, presence of lung metastases, or underlying pulmonary medical history, has yet to be identified.

Asymptomatic subjects were typically managed with dose delay. Subjects with Grade 2 pneumonitis were managed with dose delay, treated with corticosteroids, and had resolution of pneumonitis within days to weeks. In cases where nivolumab treatment was restarted, recurrence of pneumonitis was infrequently reported across the nivolumab program. Subjects with more severe cases of pneumonitis can be difficult to treat. In a few cases, subjects who did not initially respond to corticosteroids were administered anti-tumor necrosis factor therapy (infliximab) and/or cyclophosphamide. In some of these cases, pneumonitis began to resolve following the use of these additional therapies.

Guidelines on the recommended management of pneumonitis and other pulmonary AEs are found in Appendix 3. Early recognition and treatment of pneumonitis is critical to its management. Subjects should be advised to seek medical evaluation promptly if they develop new-onset dyspnea, cough, or fever or if they have worsening of these baseline symptoms. As respiratory symptoms are common in subjects with cancer (eg, NSCLC), it is important that an evaluation/work-up distinguishes between non-drug-related causes (eg, infection or progression of disease) and a possible drug-related pulmonary toxicity as the management of these events can be quite different. For symptomatic nivolumab-related pneumonitis, the principal treatment is corticosteroids (Appendix 3). All subjects with Grade 3-4 pneumonitis should discontinue

nivolumab and initiate treatment with high doses of corticosteroids. Consultation with a BMS medical monitor should be sought for all suspected cases of pneumonitis.

Gastrointestinal Adverse Events

Gastrointestinal AEs have been observed following treatment with nivolumab. Most cases of diarrhea were of low grade (Grade 1-2). Colitis occurred less frequently than diarrhea. High-grade cases of diarrhea and colitis were managed with corticosteroids and, in all cases, the events resolved.

The recommended management of GI AEs is provided in Appendix 3. Early recognition and treatment of diarrhea and colitis are critical to their management. Subjects should be advised to seek medical evaluation if they develop new-onset diarrhea, blood in stool, or severe abdominal pain or if they have worsening of baseline diarrhea. As GI symptoms are common in subjects with cancer, it is important that an evaluation/work-up distinguishes between non-drug-related causes (eg, infection or progression of disease) and a possible drug-related AE as the management can be quite different. The principal treatment for high-grade GI AEs is corticosteroids (Appendix 3). Caution should be taken in the use of narcotics in subjects with diarrhea, colitis, or abdominal pain as pain medicines may mask the signs of colonic perforation. Consultation with a BMS medical monitor should be sought for all moderate- and high-grade cases of GI AEs.

Diverticular Perforation

The prevalence of diverticulosis in the general population is common and increases with age from 10% under 40 years of age to approximately 50% over 60 years of age. Approximately 10% to 25% of subjects with diverticulosis develop diverticulitis. Perforation occurs in 50% to 70% of instances of complicated diverticulitis. [27,28] Corticosteroids, non-steroidal anti-inflammatory drugs (NSAIDs), and opioid analgesics are known risk factors for diverticular perforation. [27] Given the high prevalence of diverticulosis and diverticulitis in the general population, it is expected that some nivolumab-treated subjects will have these conditions concurrently with their malignancy. Cases of diverticular perforation while on concomitant corticosteroids (6 cases) or NSAID (1 case) were observed in nivolumab program. While there is insufficient evidence to suggest that diverticulosis or diverticulitis is a predisposing factor for GI perforation following nivolumab administration, clinical caution should be exercised, as appropriate, for subjects on concomitant medications of corticosteroids, NSAID, or opioid analgesics. In addition, be vigilant for signs and symptoms of potential perforation, especially in subjects with known diverticular disease.

Hepatic Adverse Events

Hepatic AEs, including elevated liver function tests (LFTs) and, infrequently, DILI, have been observed following treatment with nivolumab. Most cases were of low or moderate grade. Higher-grade hepatic AEs, including DILI, were managed with corticosteroids (with or without mycophenolate mofetil) and, in almost all cases, the events resolved.

The recommended management of hepatic AEs is provided in Appendix 3. Early recognition and treatment of elevated LFTs and DILI are critical to their management. Subjects should be advised to seek medical evaluation if they notice jaundice (yellow appearance of skin or sclera) or if they develop bruising, bleeding, or right-sided abdominal pain. Physicians should monitor LFTs prior to each nivolumab treatment. As LFT abnormalities are common in subjects with cancer, it is important that an evaluation/work-up distinguishes between non-drug-related causes (eg, infection, progression of disease, concomitant medications, or alcohol) and a possible drug-related AE as the management can be quite different. The principal treatment for high-grade hepatic AEs is corticosteroids (Appendix 3). Consultation with a BMS medical monitor should be sought for all moderate- and high-grade hepatic AEs.

Endocrinopathies

Endocrinopathies have been observed following treatment with nivolumab (Section 5.5). Most cases were of low or moderate grade. The events have typically been identified through either routine periodic monitoring of specific laboratories (eg, TSH) or as part of a work-up for associated symptoms (eg, fatigue). Events may occur within weeks of beginning treatment, but also have been noted to occur after many months (while still on treatment). More than 1 endocrine organ may be involved (eg, hypophysitis [pituitary inflammation] may need to be evaluated at the time adrenal insufficiency or thyroid disorder is suspected). Moderate- to high-grade cases were managed with hormone replacement therapy and, in some cases, with the addition of corticosteroids. In some cases, nivolumab treatment was held until adequate hormone replacement was provided.

Guidelines on the recommended management of endocrinopathies are provided in Appendix 3. Early recognition and treatment of endocrinopathies are critical to its management. Subjects should be advised to seek medical evaluation if they notice new-onset fatigue, lightheadedness, or difficulty with vision or if baseline fatigue worsens. As fatigue is common in subjects with cancer, it is important that an evaluation/work-up distinguishes between non-drug-related causes (eg, progression of disease, anemia, concomitant medications, or depression) and a possible drug-related AE as the management can be quite different. The principal management of endocrinopathies is hormone replacement therapy. For subjects with moderate- or high-grade events, corticosteroids may also be used (Appendix 3). Consultation with a BMS medical monitor should be sought for all moderate- and high-grade cases of endocrinopathies.

Skin Adverse Events

Rash and pruritus were the most common skin AEs observed following treatment with nivolumab. The rash was typically focal with a maculopapular appearance occurring on the trunk, back, or extremities. Most cases have been of low or moderate grade. In some cases, rash and pruritus resolved without intervention. Topical corticosteroids have been used for some cases of rash. Anti-histamines have been used for some cases of pruritus. More severe cases responded to systemic corticosteroids.

Subjects should be advised to seek medical evaluation if they notice new-onset rash. Early consultation with a dermatology specialist and a biopsy should be considered if there is uncertainty as to the cause of the rash, or if there is any unusual appearance or clinical feature associated with it. Other drugs that may cause rash should be considered in the differential and, if possible, discontinued. Three cases of toxic epidermal necrolysis occurred in subjects receiving either nivolumab+ipilimumab and prophylaxis with trimethoprim/sulfamethoxazole, alternate dosing with ipilimumab and nivolumab, or nivolumab monotherapy. These cases highlight the possible importance of discontinuing suspected drugs in the management of rash. In addition, careful evaluation of potential benefit-risk is necessary when considering the use of nivolumab or ipilimumab in a patient who has previously experienced a severe or life-threatening skin adverse reaction on a prior immune-stimulating therapy.

The principal treatment for skin AEs, such as rash and pruritus, consists of symptomatic management. Topical corticosteroids can be used for low- to moderate-grade focal rash. Systemic corticosteroids should be used for diffuse and high-grade rash. Consultation with a BMS medical monitor should be sought for all moderate- and high-grade cases of skin AEs.

Renal Adverse Events

Elevated creatinine and biopsy-confirmed tubulointerstitial nephritis and allergic nephritis have been infrequently observed following treatment with nivolumab. The frequency of renal AEs may be greater with nivolumab combination therapies than with nivolumab monotherapy. Most cases

were Grade 2 or 3 and based on creatinine elevation. subjects with a history of RCC or prior nephrectomy did not appear to be at higher risk. Events were managed with corticosteroids and, in all cases, renal function partially or fully improved.

The recommended management of renal AEs is provided in Appendix 3. Physicians should monitor creatinine regularly. As creatinine abnormalities are common in subjects with cancer and other comorbidities, it is important that an evaluation/work-up distinguishes between non-drug-related causes (eg, dehydration, concomitant medications, hypotension, or progression of disease) and a possible drug-related AE as the management can be quite different. The principal treatment for renal AEs is corticosteroids (Appendix 3). Consultation with a BMS medical monitor should be sought for all moderate- and high-grade cases of renal AEs.

<u>Neurologic Adverse Events</u>
Neurologic AEs have been uncommonly observed following treatment with nivolumab. The frequency of neurologic AEs may be greater with nivolumab + ipilimumab combination therapies than with nivolumab monotherapy or other nivolumab combinations. Neurologic AEs can manifest as central abnormalities (eg, aseptic meningitis, encephalopathy, or encephalitis) or peripheral sensory/motor neuropathies (eg, Guillain-Barre Syndrome, myasthenia gravis complicated with sepsis and fatality).

The recommended management of neurologic AEs is provided in Appendix 3. Early recognition and treatment of neurologic AEs is critical to its management. Subjects should be advised to seek medical evaluation if they notice impairment in motor function (eg, weakness), changes in sensation (eg, numbness), or symptoms suggestive of possible central nervous system abnormalities such as new headache or mental status changes. As neurologic symptoms can be common in subjects with cancer, it is important that an evaluation/work-up distinguishes between non-drug-related causes (eg, progression of disease, concomitant medications, or infection) and a possible drug-related AE as the management can be quite different. The principal treatments for neurologic toxicity are dose delay, corticosteroids, and IV immunoglobulin as outlined in the safety algorithm (Appendix 3). For high-grade related neurological AEs, nivolumab should be discontinued. Consultation with a BMS medical monitor should be sought for all moderate- and high-grade cases of neurologic AEs.

<u>Lipase/Amylase Elevations</u>
Asymptomatic elevations in lipase and amylase have been reported. In monotherapy studies, lipase and amylase levels were not systematically monitored, so an estimate of the frequency of asymptomatic lipase/amylase elevations is unknown. In studies evaluating the safety of the nivolumab + ipilimumab combination in multiple tumor types, lipase and amylase levels were systematically monitored, and elevations in any grade of lipase/amylase were consistently noted in approximately 10% to 30% of subjects. Very few subjects reported associated symptoms (eg, abdominal pain) or radiographic findings (eg, stranding) consistent with pancreatitis. Thus, there does not seem to be clinical significance to the elevated laboratory values.

As lipase/amylase abnormalities are not uncommon in subjects with cancer, it is important that an evaluation/work-up distinguishes between non-drug-related causes (eg, progression of disease, concomitant medications, or alcohol) and a possible drug-related cause as the management can be quite different. The recommended management of nivolumab-related elevated lipase/amylase values centers around close observation. Physicians should ensure that subjects have no associated symptoms consistent with pancreatitis, such as abdominal pain. Corticosteroids do not seem to alter the natural history of lipase/amylase elevations. Laboratory values tend to fluctuate on a day-to-day basis and eventually return to baseline or low grade over the course of weeks, whether or not subjects receive corticosteroids. Asymptomatic elevations should be monitored

approximately on a biweekly basis, and nivolumab should be held per protocol instructions. For sustained asymptomatic Grade 4 elevations, nivolumab should be discontinued per protocol instructions. For subjects with elevated lipase/amylase and symptoms consistent with possible pancreatitis, nivolumab should be discontinued, and consultation with a gastroenterologist should be considered. Consultation with a BMS medical monitor should be sought for all high-grade cases of elevated lipase/amylase.

Uveitis and Visual Complaints
Immune therapies have been uncommonly associated with visual complaints. Inflammation of components within the eye (eg, uveitis) is an uncommon, but clinically important, event. Uveitis may occur more frequently with nivolumab + ipilimumab combination therapy than with nivolumab monotherapy or nivolumab in combination with other therapies. An ophthalmologist should evaluate visual complaints with examination of the conjunctiva, anterior and posterior chambers, and retina. Topical corticosteroids may be used to manage low-grade events. Low-grade events that do not resolve and high-grade events should be managed with systemic corticosteroids. Consultation with a BMS medical monitor should be sought for all cases of ocular inflammatory events. Complaints of double vision should also prompt medical evaluation. In addition to ocular inflammatory events, a work-up should also consider pituitary inflammation as a cause.

Infusion Reactions
Infusion reactions, including high-grade hypersensitivity reactions, following administration of nivolumab are uncommon. Investigators are advised to monitor for fever, chills, shakes, itching, rash, hypertension or hypotension, or difficulty in breathing during and immediately after administration of nivolumab.

Dose Modifications: dose reductions or dose escalations are not permitted.

Dose Delay Criteria
Because of the potential for clinically meaningful nivolumab-related AEs requiring early recognition and prompt intervention, management algorithms have been developed for suspected AEs of selected categories. [see current Investigator Brochure and Appendix for citation examples].

Dose delay criteria apply for all drug-related adverse events (regardless of whether or not the event is attributed to nivolumab). All study drugs must be delayed until treatment can resume.

Dose delay criteria apply for all drug-related AEs. Nivolumab must be delayed until treatment can resume.

Nivolumab administration should be delayed for the following:
Any Grade ≥ 2 non-skin, drug-related AE, with the following exceptions:
• Grade 2 drug-related fatigue or laboratory abnormalities do not require a treatment delay
• Any Grade 3 skin, drug-related AE

Any Grade 3 drug-related laboratory abnormality, with the following exceptions for lymphopenia, leukopenia, AST, ALT, total bilirubin, or asymptomatic amylase or lipase:
• Grade 3 lymphopenia or leukopenia does not require dose delay.
• If a subject enrolled with a baseline AST, ALT, or total bilirubin that was within normal limits, and develops new Grade ≥ 2 AST, ALT, or bilirubin toxicity delay dosing until less than or equal to grade 1.

• If a subject enrolled with a baseline AST, ALT, or total bilirubin greater than normal but less than or equal to Grade 1 toxicity range, and develops a new Grade ≥ 3 AST, ALT, or bilirubin toxicity, delay dosing until less than equal to grade 1.
• Any Grade ≥ 3 drug-related amylase or lipase abnormality that is not associated with symptoms or clinical manifestations of pancreatitis does not require dose delay. The Investigator should be consulted for such Grade ≥ 3 amylase or lipase abnormalities.
Any AE, laboratory abnormality, or intercurrent illness which, in the judgment of the investigator warrants delaying the dose of study medication.

Subjects who require delay of nivolumab should be re-evaluated weekly or more frequently if clinically indicated and resume nivolumab dosing when re-treatment criteria are met.

Criteria to Resume Treatment
Subjects may resume treatment with study drug when the drug-related AE(s) resolve to Grade ≤1 or baseline value, with the following exceptions:
• Subjects may resume treatment in the presence of Grade 2 fatigue
• Subjects who have not experienced a Grade 3 drug-related skin AE may resume treatment in the presence of Grade 2 skin toxicity.

11.1.3 Overdose, Warnings, and Precautions
There is no available information concerning overdose with nivolumab. Depending on the symptoms and/or signs leading to the suspicion of overdose, supportive medical management should be provided. There is no specific antidote.

Overdose

An overdose is defined as the accidental or intentional administration of any dose of a product that is considered both excessive and medically important. All occurrences of overdose must be reported as an SAE.

Other Safety Considerations

Any significant worsening noted during interim or final physical examinations, electrocardiograms, x rays, and any other potential safety assessments, whether or not these procedures are required by the protocol, should also be recorded as a nonserious or serious AE, as appropriate, and reported accordingly.

12.0 CRITERIA FOR THERAPEUTIC RESPONSE/OUTCOME ASSESSMENT

Antitumor Effect
Response and progression will be evaluated in this study using the new international criteria proposed by the revised Response Evaluation Criteria in Solid Tumors (RECIST) guideline (version 1.1) [22]. Changes in the largest diameter (unidimensional measurement) of the tumor lesions and the shortest diameter in the case of malignant lymph nodes are used in the RECIST criteria.

12.1 Definitions

Evaluable for toxicity. All patients who receive at least one dose of nivolumab will be evaluable for toxicity. Patients will be considered evaluable for toxicity from the time of their first treatment on study.

Evaluable for objective response. Only those patients who have measurable disease present at baseline, have received at least one dose of therapy, and have had their disease reevaluated will be considered evaluable for response. These patients will have their response classified according to the definitions stated below. (Note: Patients who exhibit objective disease progression prior to the end of cycle 1 will also be considered evaluable.)

Evaluable Non-Target Disease Response. Patients who have lesions present at baseline that are evaluable but do not meet the definitions of measurable disease, have received at least one dose of therapy, and have had their disease reevaluated will be considered evaluable for non-target disease. The response assessment is based on the presence, absence, or unequivocal progression of the lesions.

12.2 Disease Parameters

Measurable disease. Measurable lesions are defined as those that can be accurately measured in at least one dimension (longest diameter to be recorded) as >20 mm by chest x-ray, as >10 mm with CT scan, or >10 mm with calipers by clinical exam. All tumor measurements must be recorded in millimeters (or decimal fractions of centimeters).

Note: Tumor lesions that are situated in a previously irradiated area will not be considered measurable unless progression is documented or a biopsy is obtained to confirm persistence at least 90 days following completion of radiation therapy.

Malignant lymph nodes. To be considered pathologically enlarged and measurable, a lymph node must be >15 mm in short axis when assessed by CT scan (CT scan slice thickness recommended to be no greater than 5 mm). At baseline and in follow-up, only the short axis will be measured and followed.

Non-measurable disease. All other lesions (or sites of disease), including small lesions (longest diameter <10 mm or pathological lymph nodes with ≥ 10 to <15 mm short axis), are considered non-measurable disease. Bone lesions, leptomeningeal disease, ascites, pleural/pericardial effusions, lymphangitis cutis/pulmonitis, inflammatory breast disease, and abdominal masses (not followed by CT or MRI), are considered as non-measurable.

Note: Cystic lesions that meet the criteria for radiographically defined simple cysts should not be considered as malignant lesions (neither measurable nor non-measurable) since they are, by definition, simple cysts.

'Cystic lesions' thought to represent cystic metastases can be considered as measurable lesions, if they meet the definition of measurability described above. However, if non-cystic lesions are present in the same patient, these are preferred for selection as target lesions.

Target lesions. All measurable lesions up to a maximum of 2 lesions per organ and 5 lesions in total, representative of all involved organs, should be identified as target lesions and recorded and measured at baseline. Target lesions should be selected on the basis of their size (lesions with the longest diameter), be representative of all involved organs, but in addition should be those that lend themselves to reproducible repeated measurements. It may be the case that, on occasion, the largest lesion does not lend itself to reproducible measurement in which circumstance the next largest lesion which can be measured reproducibly should be selected. A sum of the diameters (longest for non-nodal lesions, short axis for nodal lesions) for all target lesions will be calculated and reported as the baseline sum diameters. If lymph nodes are to be included in the sum, then only the short axis is added into the sum. The baseline sum diameters will be used as reference to further characterize any objective tumor regression in the measurable dimension of the disease.

Non-target lesions. All other lesions (or sites of disease) including any measurable lesions over and above the 5 target lesions should be identified as non-target lesions and should also be recorded at baseline. Measurements of these lesions are not required, but the presence, absence, or in rare cases unequivocal progression of each should be noted throughout follow-up.

12.3 Methods for Evaluation of Measurable Disease

All measurements should be taken and recorded in metric notation using a ruler or calipers. All baseline evaluations should be performed as closely as possible to the beginning of treatment and never more than 4 weeks before the beginning of the treatment. The same method of assessment and the same technique should be used to characterize each identified and reported lesion at baseline and during follow-up. Imaging-based evaluation is preferred to evaluation by clinical examination unless the lesion(s) being followed cannot be imaged but are assessable by clinical exam.

Clinical lesions: Clinical lesions will only be considered measurable when they are superficial (e.g., skin nodules and palpable lymph nodes) and ≥10 mm diameter as assessed using calipers (e.g., skin nodules). In the case of skin lesions, documentation by color photography, including a ruler to estimate the size of the lesion, is recommended.

Chest x-ray: Lesions on chest x-ray are acceptable as measurable lesions when they are clearly defined and surrounded by aerated lung. However, CT is preferable.

Conventional CT and MRI: This guideline has defined measurability of lesions on CT scan based on the assumption that CT slice thickness is 5 mm or less. If CT scans have slice thickness greater than 5 mm, the minimum size for a measurable lesion should be twice the slice thickness. MRI is also acceptable in certain situations (e.g. for body scans), but NOT lung.

CA125: CA125 alone cannot be used to assess response. If CA125 is initially above the upper normal limit, it must normalize for a patient to be considered in complete clinical response.

12.4 Response Criteria

Evaluation of Target Lesions

Complete Response (CR): Disappearance of all target lesions. Any pathological lymph nodes (whether target or non-target) must have reduction in short axis to <10 mm.

Partial Response (PR): At least a 30% decrease in the sum of the diameters of target lesions, taking as reference the baseline sum diameters.

Progressive Disease (PD): At least a 20% increase in the sum of the diameters of target lesions, taking as reference the smallest sum on study (this includes the baseline sum if that is the smallest on study). In addition to the relative increase of 20%, the sum must also demonstrate an absolute increase of at least 5 mm. (Note: the appearance of one or more new lesions is also considered progressions).

Stable Disease (SD): Neither sufficient shrinkage to qualify for PR nor sufficient increase to qualify for PD, taking as reference the smallest sum diameters while on study.

Evaluation of Non-Target Lesions

Complete Response (CR): Disappearance of all non-target lesions and normalization of tumor marker level. All lymph nodes must be non-pathological in size (<10 mm short axis). Note: If CA125 is initially above the upper normal limit, it must normalize for a patient to be considered in complete clinical response.

Non-CR/Non-PD: Persistence of one or more non-target lesion(s) and/or maintenance of CA125 level above the normal limits.

Progressive Disease (PD): Appearance of one or more new lesions and/or unequivocal progression of existing non-target lesions. Unequivocal progression should not normally trump target lesion status. It must be representative of overall disease status change, not a single lesion increase.

Although a clear progression of “non-target” lesions only is exceptional, the opinion of the treating physician should prevail in such circumstances, and the progression status should be confirmed at a later time by the review panel (or Principal Investigator).

Evaluation of Best Overall Response

The best overall response is the best response recorded from the start of the treatment until disease progression/recurrence (taking as reference for progressive disease the smallest measurements recorded since the treatment started). The patient's best response assignment will depend on the achievement of both measurement and confirmation criteria.

For Patients with Measurable Disease (i.e., Target Disease)

Target Lesions	Non-Target Lesions	New Lesions	Overall Response	Best Overall Response when Confirmation is Required*
CR	CR	No	CR	≥4 wks. Confirmation**
CR	Non-CR/Non-PD	No	PR	≥4 wks. Confirmation**
CR	Not evaluated	No	PR	
PR	Non-CR/Non-PD/not evaluated	No	PR	
SD	Non-CR/Non-PD/not evaluated	No	SD	Documented at least once ≥4 wks. from baseline**
PD	Any	Yes or No	PD	no prior SD, PR or CR
Any	PD***	Yes or No	PD	
Any	Any	Yes	PD	

* See RECIST 1.1 manuscript for further details on what is evidence of a new lesion.
** Only for non-randomized trials with response as primary endpoint.

***	In exceptional circumstances, unequivocal progression in non-target lesions may be accepted as disease progression.

Note: Patients with a global deterioration of health status requiring discontinuation of treatment without objective evidence of disease progression at that time should be reported as "*symptomatic deterioration.*" Every effort should be made to document the objective progression even after discontinuation of treatment.

For Patients with Non-Measurable Disease (i.e., Non-Target Disease)

Non-Target Lesions	New Lesions	Overall Response
CR	No	CR
Non-CR/non-PD	No	Non-CR/non-PD*
Not all evaluated	No	not evaluated
Unequivocal PD	Yes or No	PD
Any	Yes	PD

* 'Non-CR/non-PD' is preferred over 'stable disease' for non-target disease since SD is increasingly used as an endpoint for assessment of efficacy in some trials so to assign this category when no lesions can be measured is not advised.

Duration of Response

Duration of overall response: The duration of overall response is measured from the time measurement criteria are met for CR or PR (whichever is first recorded) until the first date that recurrent or progressive disease is objectively documented (taking as reference for progressive disease the smallest measurements recorded since the treatment started). The duration of overall CR is measured from the time measurement criteria are first met for CR until the first date that progressive disease is objectively documented.

Duration of stable disease: Stable disease is measured from the start of the treatment until the criteria for progression are met, taking as reference the smallest measurements recorded since the treatment started, including the baseline measurements.

Progression-Free Survival

Progression-Free Survival (PFS) is defined as the duration of time from start of treatment to time of recurrence, progression (as defined by RECIST 1.1), or death, whichever occurs first.

Survival

Survival is defined as the duration of time from start of treatment to time of death or the date of last contact

Permitted Deviations from RECIST

The study's efficacy objectives will be evaluated according to the standard, unmodified RECIST v1.1 criteria described above, and that, within the context of this protocol, the only purpose of the

modifications to the criteria is to allow certain patients to continue the study treatment despite meeting RECIST criteria for progression of disease.

The response to immunotherapy may differ from the typical responses observed with cytotoxic chemotherapy including the following (Wolchok et al 2009, Nishino et al 2013):

- Response to immunotherapy may be delayed
- Response to immunotherapy may occur after POD by conventional criteria
- The appearance of new lesions may not represent POD with immunotherapy
- SD while on immunotherapy may be durable and represent clinical benefit.

As long as they are receiving treatment on protocol, patients will be permitted to continue study treatment after RECIST v 1.1 criteria for POD are met at the first 12 week (+/- 7 days) CT if they meet all of the following criteria:

- No decrease in performance status
- No requirement for immediate alternative treatment or urgent palliative treatment
- Progression limited to an increase of 40% in the sum of diameters of target lesions
- No more than 4 new lesions included in the sum.

For patients who continue treatment in the case of radiologic progression at the first 12 week (+/- 7 days) CT:

- At any subsequent CT scan patients who have stable disease as compared to the 12 week (+/- 7 days) CT scan will be allowed to continue on study treatment.
- Patients who continue treatment in the case of radiologic progression at the first 12 week (+/- 7 days) CT, and later experience a PR or CR (as compared to baseline CT) will be recorded as delayed responses.

Modification of RECIST as described may discourage the early discontinuation of Nivolumab and provide a more complete evaluation of its anti-tumor activity than would be seen with conventional response criteria. Nonetheless, the efficacy analysis will be conducted by programmatically deriving each efficacy endpoint based on RECIST v 1.1 criteria.

The primary reason for study treatment discontinuation should be documented on the eCRF.

Of note, clinically significant deterioration is considered to be a rapid tumor progression that necessitates treatment with anti-cancer therapy other than nivolumab or with symptomatic progression that requires urgent medical intervention (e.g., central nervous system metastasis, respiratory failure due to tumor, spinal cord compression)

13.0 CRITERIA FOR REMOVAL FROM STUDY

Patients may withdraw from the study at any time. Any patient who withdraws will be encouraged to return to the study center for a treatment completion visit. Patients who discontinue early should return within 30 days following the final dose of study treatment. The primary reason for discontinuation must be recorded in the medical record.

Patients may be withdrawn from the study if they experience any of the following:

- Disease progression, per investigator assessment
- Intolerable toxicity of study drugs

Other reasons for patient discontinuation may include, but are not limited to, the following:

- Change in patient eligibility
- Non-compliance
- Patient decision
- If treatment is delayed for more than 28 consecutive days

The investigator has the right to discontinue a patient from the study for any medical condition that the investigator determines may jeopardize the patient's safety if he or she continues in the study; for reasons of noncompliance (e.g., missed doses, visits); or if the investigator determines it is in the best interest of the patient.

14.0 BIOSTATISTICS

This study aims to assess the activity of single-agent nivolumab in patients with recurrent or persistent MSI-H, MMR-D, or hypermutated uterine cancer. All patients will be treated with nivolumab at a dose of 480 MG given every 4 weeks, until disease progression, or intolerable toxicity. The objective response would be considered the best response observed at either the 12 or 24 week radiographic evaluation.

The null hypothesis relating to uninteresting levels of activity was chosen in accordance with previously published reports of cytotoxic and biologic and anti-vascular agent activity in endometrial cancers. Further details are outlined in Aghajanian, et al. Phase II trial of bevacizumab in recurrent or persistent endometrial cancer: A Gynecologic Oncology Group Study. J Clin Oncol 29: 2259-2265, 2011. In that study, bevacizumab was considered to be an active agent with 7/52 evaluable patients (13.5%) achieving objective response, and 21/52 patients (40.4%) surviving progression free for at least 6 months.

<u>Evaluation of overall response rate (ORR)</u>
ORR (defined as complete response (CR)+ partial response (PR)) will be the primary endpoint. A 5% ORR will be considered non-promising and 25% of higher will be considered promising for further study. Forty patients provide 95% power and Type 1 error of 2.5% using a Simon two stage design. There will be an interim analysis after 23 patients and if 2 or more responders (CR+PR) are observed out of 23 patients then the study will continue to the second stage. At the end of the study we require 6 or more responders out of 40 patients to declare this study positive and the agent worthy for further investigation.

Unacceptable response rate: 0.05
Desirable response rate: 0.25
Error rates: Type I = 0.025 ; Type II = 0.05
(using minimax Simon 2 stage design)

# responses required in Stage I	# patients accrued in Stage I	# responses required in Stage I+II	Total Sample Size
>=2	23	>=6	40

<u>Evaluation of progression-free survival (PFS) at 24 weeks</u>
PFS at 24 weeks is the co-primary endpoint. For PFS rate at 24 weeks, we consider a 25% rate as non-promising and a rate of 50% or higher as indicative of promising clinical activity. There will be an interim analysis after the first 23 patients are accrued, and if 5 or more of the first 17 patients accrued are progression free at 24 weeks then the study will continue to the second stage. At the end of the study we require 16 or more patients to be progression free at 24 weeks in order to declare the study positive and the agent worthy for further investigation. This study will have 91% power and 2.5% Type 1 error to show activity in terms of 24 weeks PFS rate. The study will continue if *either* overall response rate (ORR) *or* progression-free survival rate (PFS) is promising. If we see 1 or fewer objective responses in stage 1 out of 23 patients then we will hold accrual to determine if at least 5/17 remain progression free at 24 weeks. The first 17 patients will be included in the assessment of 24 week PFS rate.

Unacceptable 24 week PFS rate: 0.25
Desirable 24 week PFS rate: 0.50
Error rates: Type I = 0.025 ; Type II = 0.09
(using minimax Simon design with binary endpoint))

# patients with increase in PFS required in Stage I	# patients accrued in Stage I	# patients with increase in PFS required in Stage I+II	Total Sample Size
>=5	17	>=16	40

The overall Type I error of the study is 5%, the combined Type I error of the two primary endpoints of the study.

Based on historical rates of recurrent MSI-H, MMR-D, or hypermutated uterine cancer in our study population, we anticipate successful enrollment of 1 patients per month yielding a total accrual time of 24 months. If 2 or more responders (CR+PR) are seen in the first 23 patients **or** 5 patients of the first 17 enrolled remain progression free at 24 weeks, whichever is met first, then accrual will continue to stage II. If we see 1 or fewer responders (CR+PR) in the first 23 patients enrolled, then we will halt accrual between stages and wait until we get 24 week PFS results on the first 17 patients accrued in order to determine if we can go onto stage II based on promising PFS.

Efficacy Variables
The intent of this protocol is to assess single-agent nivolumab for activity in patients with recurrent MSI-H, MMR-D, or hypermutated uterine cancers. There are no treatment comparisons involved.

The principal parameters employed to evaluate the efficacy of the combination are:

- The rate of ORR and duration of overall objective response (ORR = CR + PR).
- The frequency and severity of observed adverse effects.
- Progression-free survival rate at 24 weeks for all patients evaluable for this endpoint.

Evaluable for efficacy (response and PFS): those who have measurable disease at baseline and have received at least one dose of therapy and had at least one post-baseline efficacy assessment. Patients who are evaluable for response and are lost to follow up or die before the 24 week PFS assessment will be considered as events (failures). The primary analysis for efficacy will include only patients who are evaluable for response. A sensitivity analysis will include all patients who have measurable disease at baseline and have received at least one dose of therapy, but do not have a post baseline assessment of response, considering them as non responders for ORR and events for PFS.
For safety patients who received at least one dose of therapy will be included.

Definitions of Statistics to be Reported

- Disease Control Rate (DCR), defined as the percentage of patients with complete response (CR) + partial response (PR) + stable disease (SD) ≥12 weeks from the start of treatment will be reported and the 90% confidence interval will be estimated using exact binomial proportions. This will be done following RECIST criteria.
- Progression free survival (PFS), defined as the duration of time from start of treatment to time of recurrence, progression, or death due to any cause, whichever occurs first. Patients will be censored at last follow up date. The Kaplan Meier estimate of median PFS will be reported as well as the PFS rate at 24 weeks
- Overall survival (OS), defined as the duration of time from start of treatment until the date of death due to any cause. Patients will be censored at last follow up date. The Kaplan Meier estimate of median OS will be reported.
- Duration of response (DOR), defined as the time from which measurement criteria are met for CR or PR (whichever status is recorded first using RECIST) until the first date of documented disease progression, will be estimated using the Kaplan Meier method. Patients without documented progression will be censored at last follow up.
- Adverse events by the current version of Common Terminology Criteria for Adverse Events version 4 (CTCAE v4.03) will be tabulated in order to assess the safety profile and tolerability therapy in treated patients.

Safety Analysis

The rates of irAE, SAE, therapy completion rate, and protocol completion will be reported.

Safety will be measured by the frequency of grade 3 or 4 treatment-related clinically significant toxicities (NCI Common Terminology Criteria for Adverse Events version 4.0), aside from Grade 3 or 4 hematologic or laboratory abnormalities, unless they are deemed unexpected or clinically significant.
Frequencies of toxicities will be tabulated. The grade 3 or 4 treatment-related non-hematologic/non-laboratory toxicity will be estimated at the end of the trial along with the 95% confidence interval. With 40 patients, these rates can be estimated within ±16%.

AEs will be collected from the time of study enrollment until 4 weeks after last dose of study drug delivered.

Exploratory Objectives:

Objective 1: Correlate mutational burden with clinical benefit from nivolumab, both overall response rate (ORR) and progression-free survival (PFS) rate at 24 weeks. Mutational burden will be measured by the number of somatic mutations found in genomic profile by IMPACT (continuous number). Two separate analyses will be done for each efficacy outcome : ORR or PFS at 24 weeks which are considered binary here and thus the analysis will test whether the median number of somatic mutation is different in the group of responders vs non responders. A non parametric test will be used to test differences in the distribution of somatic mutations in the 2 groups (responders vs non responders) such as Wilcoxon rank sum test.

Objective 2: Correlate the somatic mutational burden identified by MSK-IMPACT with the MSIsensor algorithm score. The MSK-IMPACT data will also be analyzed by the MSIsensor algorithm. We will assess the relationship between MSIsensor score and the number of somatic mutations. MSIsensor score is a binary variable; any score <10 is MSI stable, any score 10 or higher is MSI high. We will test whether the median mutational burden (continuous variable) is different among patients with MSIsensor score < 10 (MSI stable) or MSIsensor score 10 or higher (MSI high). Wilcoxon rank sum test will be used to test differences in 2 groups.

Objective 3: Correlate the MSIsensor score with the MMR IHC status (immunohistochemical staining for mismatch repair enzyme proteins). We will test for agreement between the MSIsensor score (binary) with MMR status by IHC which is measured as retained or absent (binary). The goal of the above aims is to explore the agreement between “MMR IHC absent”, and “MSIsensor score high” and “mutational burden high”. McNemar’s test will be used to test agreement between MSI sensor and MMR status.

Objective 4: Test for association between pre-treatment immune phenotype (tumor infiltrating lymphocytes and PD-L1) and best overall response rate (ORR) and progression free survival rate at 24 weeks. TILs and PD-L1 are both biomarkers without a specific cutoff that defines positive and negative. For these reasons these will be analyzed as continuous variables. Two separate analysis will be done for each efficacy outcome : ORR or PFS at 24 weeks which are considered binary here and thus the analysis will test whether the median biomarker value is different in the group of responders vs non responders. Wilcoxon rank sum test will be used to test differences in 2 groups.

15.0 RESEARCH PARTICIPANT REGISTRATION AND RANDOMIZATION PROCEDURES

15.1 Research Participant Registration

Confirm eligibility as defined in the section entitled Criteria for Patient/Subject Eligibility.

Obtain informed consent, by following procedures defined in section entitled Informed Consent Procedures.

During the registration process registering individuals will be required to complete a protocol specific Eligibility Checklist.

The individual signing the Eligibility Checklist is confirming whether or not the participant is eligible to enroll in the study. Study staff are responsible for ensuring that all institutional requirements

necessary to enroll a participant to the study have been completed. See related Clinical Research Policy and Procedure #401 (Protocol Participant Registration).

15.2 Randomization

N/A

16.0 DATA MANAGEMENT ISSUES

A research study assistant (RSA) will be assigned to study. The responsibilities of the RSA include project compliance, data collection, extraction and data entry, data reporting, coordination of the activities of the protocol study team and, and of the flow of regulatory paperwork.

The data collected for the study will be entered into a secure database (CRDB). All routine blood test results required per the protocol will be captured in CRDB in addition to baseline medical conditions and disease information, response assessments, off-study documentation, and toxicity grade and attribution. Source documentation will be available to support the computerized patient record.

MSK will hold the IND. MSK will be responsible for all safety monitoring. All SAEs will be reported to the MSKCC IRB. The safety of the study will be monitored by the MSKCC Data and Safety Monitoring Committee.

Weekly registration reports will be generated by the RSA and reviewed by the PI to monitor patient accruals and completeness of registration data. Routine data quality reports will be generated to assess missing data and inconsistencies.

Accrual rates and extent and accuracy of evaluations and follow up will be monitored periodically throughout the study. Recurrent lapses in data collection, deviations or violations will be discussed with the study team and a corrective plan will be generated. Accrual goals and factors impacting accrual goals will be discussed at the weekly New Patient/Protocol meetings.

If accrual proceeds more quickly than anticipated, it may be slowed or staggered at the discretion of the Principal Investigator, to account for safety concerns or data management resources.

16.1 Quality Assurance

The data and safety monitoring plan at Memorial Sloan Kettering Cancer Center was approved by the National Cancer Institute in September 2001. The plan addressed the new policies set forth by the NCI and the document entitled "Policy of the National Cancer Institute for data and safety monitoring of clinical trials" which can be found at http://grants.nih.gov/grants/guide/noticefiles/not98-084.html. The DSM plans at MSKCC were established and are monitored by the Office of Clinical Research. The MSKCC data and safety monitoring plan can be found on the MSKCC Internet at http://inside2/clinresearch/Documents/MSKCC Data and Safety Monitoring Plans.pdf. There are several different mechanisms by which clinical trials are monitored for data, safety and quality. There are institutional processes in place for quality assurance (e.g., protocol monitoring, compliance and data verification audits, therapeutic response, and staff education on clinical research QA) and departmental procedures for quality control, plus there are two institutional

committees that are responsible for monitoring the activities of our clinical trials programs. Memorial Sloan Kettering Cancer Center has set up three distinct monitoring processes for our clinical trials program. There are two sub-committees that have the responsibility of data and safety monitoring. These are joint sub-committees with dual-reporting responsibilities. The Data and Safety Monitoring Committee (DSMC) is the sub-committee responsible for monitoring all Phase 1, 2, 1/2, pilot and non-phase clinical trials. The Data and Safety Monitoring Board (DSMB) is the sub-committee responsible for monitoring Phase 3 randomized clinical trials.

The DSMC convenes once per quarter and monitors the risk participants are exposed to, the progress of the study, the adequacy of the data storage and whether sufficient data are being entered into the CRDB. The DSMC monitors phase 1, 2, 1/2, pilot and non-phase trials that are not being monitored by an industrial sponsor, and which meet the NCI definition of a Clinical Trial. This trial will qualify for monitoring by the DSMC.

During the protocol development and review process, each protocol will be assessed for its level of risk and degree of monitoring required, and the monitoring procedures will be established at the time of protocol activation. A detailed description of the data to be collected, process of data collection (i.e., data manager and/or data management office), database that will be utilized for data collection and storage (e.g., Clinical Research Database (CRDB), user-supported software), reporting requirements of the data to the institution (IRB), the sponsor and/or governing agency.

16.2 Data and Safety Monitoring

The Data and Safety Monitoring (DSM) Plans at Memorial Sloan Kettering Cancer Center were approved by the National Cancer Institute in September 2001. The plans address the new policies set forth by the NCI in the document entitled “Policy of the National Cancer Institute for Data and Safety Monitoring of Clinical Trials” which can be found at: http://www.cancer.gov/clinicaltrials/conducting/dsm-guidelines/page1. The DSM Plans at MSKCC were established and are monitored by the Office of Clinical Research. The MSKCC Data and Safety Monitoring Plans can be found on the MSKCC Intranet at: http://smskpsps9/dept/ocr/OCR%20Website%20Documents/Clinical%20Research%20Quality%20Assurance%20(CRQA)/MSKCC%20Data%20and%20Safety%20Monitoring%20Plan.pdf

17.0 PROTECTION OF HUMAN SUBJECTS

17.1 Privacy

The consent indicates that individualized de identified information collected for the purposes of this study may be shared with other qualified researchers. Only researchers who have received approval from MSK will be allowed to access this information which will not include protected health information, such as the participant's name, except for dates. It is also stated in the Research Authorization that their research data may be shared with other qualified researchers.

17.2 Serious Adverse Event (SAE) Reporting

An adverse event is considered serious if it results in ANY of the following outcomes:

- Death
- A life-threatening adverse event
- An adverse event that results in inpatient hospitalization or prolongation of existing hospitalization
- A persistent or significant incapacity or substantial disruption of the ability to conduct normal life functions
- A congenital anomaly/birth defect
- Important Medical Events (IME) that may not result in death, be life threatening, or require hospitalization may be considered serious when, based upon medical judgment, they may jeopardize the patient or subject and may require medical or surgical intervention to prevent one of the outcomes listed in this definition

Note: Hospital admission for a planned procedure/disease treatment is not considered an SAE.

SAE reporting is required as soon as the participant signs consent. SAE reporting is required for 30-days after the participant's last investigational treatment or intervention. Any events that occur after the 30-day period and that are at least possibly related to protocol treatment must be reported.

If an SAE requires submission to the IRB office per IRB SOP RR-408 'Reporting of Serious Adverse Events', the SAE report must be sent to the IRB within 5 calendar days of the event. The IRB requires a Clinical Research Database (CRDB) SAE report be submitted electronically to the SAE Office as follows:

For IND/IDE trials: Reports that include a Grade 5 SAE should be sent to saegrade5@mskcc.org. All other reports should be sent to saemskind@mskcc.org.

For all other trials: Reports that include a Grade 5 SAE should be sent to saegrade5@mskcc.org. All other reports should be sent to sae@mskcc.org.

The report should contain the following information:

Fields populated from CRDB:

- Subject's initials
- Medical record number

- Disease/histology (if applicable)
- Protocol number and title

Data needing to be entered:

- The date the adverse event occurred
- The adverse event
- The grade of the event
- Relationship of the adverse event to the treatment (drug, device, or intervention)
- If the AE was expected
- The severity of the AE
- The intervention
- Detailed text that includes the following
    - A explanation of how the AE was handled
    - A description of the subject's condition
    - Indication if the subject remains on the study
- If an amendment will need to be made to the protocol and/or consent form
- If the SAE is an Unanticipated Problem

The PI's signature and the date it was signed are required on the completed report.

For IND/IDE protocols:
The CRDB SAE report should be completed as per above instructions. If appropriate, the report will be forwarded to the FDA by the SAE staff through the IND Office.
Serious Adverse Event Collection and Reporting - All Serious Adverse Events must be reported to BMS Worldwide Safety.

Site specific forms will be requested for review.

The sponsor/investigator will be required to reconcile SAEs reported in the clinical database with SAE cases transmitted to BMS Global Pharmacovigilance (GPV&E); worldwide.safety@bms.com . BMS requests this is initiated by the sponsor investigator up to quarterly and prior to the database lock or final data summary. The process will be further defined. During reconciliation, any events found to not be reported previously to BMS must be sent to Worldwide.Safety@BMS.com.

For studies conducted under an Investigator IND in the US, any event that is both serious and unexpected must be reported to the Food and Drug Administration (FDA) as soon as possible and no later than 7 days (for a death or life-threatening event) or 15 days (for all other SAEs) after the investigator's or institution's initial receipt of the information. BMS will be provided with a simultaneous copy of all adverse events filed with the FDA.

All SAEs should simultaneously be faxed or e-mailed to BMS at:

Global Pharmacovigilance & Epidemiology

Bristol-Myers Squibb Company

Fax Number: 609-818-3804

Email: Worldwide.safety@bms.com

<u>The report should contain the following information:</u>

Fields populated from CRDB:

- Subject's name (generate the report with only initials if it will be sent outside of MSK)
- Medical record number
- Disease/histology (if applicable)
- Protocol number and title

Data needing to be entered:

- The date the adverse event occurred
- The adverse event
- The grade of the event
- Relationship of the adverse event to the treatment (drug, device, or intervention)
- If the AE was expected
- The severity of the AE
- The intervention
- Detailed text that includes the following
    - A explanation of how the AE was handled
    - A description of the subject's condition
    - Indication if the subject remains on the study
    - If an amendment will need to be made to the protocol and/or consent form.

The PI's signature and the date it was signed are required on the completed report.

Ethical conduct of the study

The study will be performed in accordance with ethical principles that have their origin in the Declaration of Helsinki and are consistent with ICH/Good Clinical Practice, and applicable regulatory requirements Subject data protection.

17.2.1

18.0 INFORMED CONSENT PROCEDURES

Before protocol-specified procedures are carried out, consenting professionals will explain full details of the protocol and study procedures as well as the risks involved to participants prior to their inclusion in the study. Participants will also be informed that they are free to withdraw from the study at any time. All participants must sign an IRB/PB-approved consent form indicating their consent to participate. This consent form meets the requirements of the Code of Federal Regulations and the Institutional Review Board/Privacy Board of this Center. The consent form will include the following:

1. The nature and objectives, potential risks and benefits of the intended study.
2. The length of study and the likely follow-up required.

3. Alternatives to the proposed study. (This will include available standard and investigational therapies. In addition, patients will be offered an option of supportive care for therapeutic studies.)
4. The name of the investigator(s) responsible for the protocol.
5. The right of the participant to accept or refuse study interventions/interactions and to withdraw from participation at any time.

Before any protocol-specific procedures can be carried out, the consenting professional will fully explain the aspects of patient privacy concerning research specific information. In addition to signing the IRB Informed Consent, all patients must agree to the Research Authorization component of the informed consent form.

Each participant and consenting professional will sign the consent form. The participant must receive a copy of the signed informed consent form.

19.0 REFERENCES

1 Siegel, R. L., Miller, K. D. & Jemal, A. Cancer statistics, 2016. *CA: a cancer journal for clinicians* **66**, 7-30, doi:10.3322/caac.21332 (2016).

2 Miller, D. *et al.* Late-Breaking Abstract 1: Randomized phase III noninferiority trial of first line chemotherapy for metastatic or recurrent endometrial carcinoma: A Gynecologic Oncology Group study. *Gynecologic oncology* **125**, 771, doi:http://dx.doi.org/10.1016/j.ygyno.2012.03.034 (2012).

3 Hensley, M. L. *et al.* Fixed-dose rate gemcitabine plus docetaxel as second-line therapy for metastatic uterine leiomyosarcoma: a Gynecologic Oncology Group phase II study. *Gynecologic oncology* **109**, 323-328, doi:10.1016/j.ygyno.2008.02.024 (2008).

4 Hensley, M. L., Blessing, J. A., Mannel, R. & Rose, P. G. Fixed-dose rate gemcitabine plus docetaxel as first-line therapy for metastatic uterine leiomyosarcoma: a Gynecologic Oncology Group phase II trial. *Gynecologic oncology* **109**, 329-334, doi:10.1016/j.ygyno.2008.03.010 (2008).

5 Aghajanian, C. *et al.* Phase II trial of bevacizumab in recurrent or persistent endometrial cancer: a Gynecologic Oncology Group study. *Journal of clinical oncology : official journal of the American Society of Clinical Oncology* **29**, 2259-2265, doi:10.1200/jco.2010.32.6397 (2011).

6 Borghaei, H. *et al.* Nivolumab versus Docetaxel in Advanced Nonsquamous Non-Small-Cell Lung Cancer. *The New England journal of medicine* **373**, 1627-1639, doi:10.1056/NEJMoa1507643 (2015).

7 Brahmer, J. *et al.* Nivolumab versus Docetaxel in Advanced Squamous-Cell Non-Small-Cell Lung Cancer. *The New England journal of medicine* **373**, 123-135, doi:10.1056/NEJMoa1504627 (2015).

8 Rizvi, N. A. *et al.* Activity and safety of nivolumab, an anti-PD-1 immune checkpoint inhibitor, for patients with advanced, refractory squamous non-small-cell lung cancer (CheckMate 063): a phase 2, single-arm trial. *The Lancet. Oncology* **16**, 257-265, doi:10.1016/s1470-2045(15)70054-9 (2015).

9 Weber, J. S. *et al.* Nivolumab versus chemotherapy in patients with advanced melanoma who progressed after anti-CTLA-4 treatment (CheckMate 037): a randomised, controlled, open-label, phase 3 trial. *The Lancet. Oncology* **16**, 375-384, doi:10.1016/s1470-2045(15)70076-8 (2015).

10 McDermott, D. F. *et al.* Survival, Durable Response, and Long-Term Safety in Patients With Previously Treated Advanced Renal Cell Carcinoma Receiving Nivolumab. *J Clin Oncol* **33**, 2013-2020, doi:10.1200/jco.2014.58.1041 (2015).
11 Motzer, R. J. *et al.* Nivolumab versus Everolimus in Advanced Renal-Cell Carcinoma. *The New England journal of medicine* **373**, 1803-1813, doi:10.1056/NEJMoa1510665 (2015).
12 Motzer, R. J. *et al.* Nivolumab for Metastatic Renal Cell Carcinoma: Results of a Randomized Phase II Trial. *J Clin Oncol* **33**, 1430-1437, doi:10.1200/jco.2014.59.0703 (2015).
13 Ansell, S. M. *et al.* PD-1 blockade with nivolumab in relapsed or refractory Hodgkin's lymphoma. *The New England journal of medicine* **372**, 311-319, doi:10.1056/NEJMoa1411087 (2015).
14 Tse, B. W., Collins, A., Oehler, M. K., Zippelius, A. & Heinzelmann-Schwarz, V. A. Antibody-based immunotherapy for ovarian cancer: where are we at? *Annals of oncology : official journal of the European Society for Medical Oncology / ESMO* **25**, 322-331, doi:10.1093/annonc/mdt405 (2014).
15 Howitt, B. E., Shukla, S. A., Sholl, L. M. & et al. ASsociation of polymerase e–mutated and microsatellite-instable endometrial cancers with neoantigen load, number of tumor-infiltrating lymphocytes, and expression of pd-1 and pd-l1. *JAMA Oncology* **1**, 1319-1323, doi:10.1001/jamaoncol.2015.2151 (2015).
16 Gajewski, T. F., Louahed, J. & Brichard, V. G. Gene signature in melanoma associated with clinical activity: a potential clue to unlock cancer immunotherapy. *Cancer journal (Sudbury, Mass.)* **16**, 399-403, doi:10.1097/PPO.0b013e3181eacbd8 (2010).
17 Battaglin, F. *et al.* Using mutational load in next generation sequencing (NGS) to identify mismatch repair (MMR) deficiency in colorectal cancer (CRC). *ASCO Meeting Abstracts* **33**, 3565 (2015).
18 Niu, B. *et al.* MSIsensor: microsatellite instability detection using paired tumor-normal sequence data. *Bioinformatics* **30**, 1015-1016, doi:10.1093/bioinformatics/btt755 (2014).
19 Le, D. T. *et al.* PD-1 Blockade in Tumors with Mismatch-Repair Deficiency. *The New England journal of medicine*, doi:10.1056/NEJMoa1500596 (2015).
20 Brahmer, J. R. *et al.* Safety and activity of anti-PD-L1 antibody in patients with advanced cancer. *The New England journal of medicine* **366**, 2455-2465, doi:10.1056/NEJMoa1200694 (2012).
21 Santin, A. D. *et al.* Regression of Chemotherapy-Resistant Polymerase epsilon (POLE) Ultra-Mutated and MSH6 Hyper-Mutated Endometrial Tumors with Nivolumab. *Clinical cancer research : an official journal of the American Association for Cancer Research* **22**, 5682-5687, doi:10.1158/1078-0432.ccr-16-1031 (2016).
22 Sharpe, A. H., Wherry, E. J., Ahmed, R. & Freeman, G. J. The function of programmed cell death 1 and its ligands in regulating autoimmunity and infection. *Nature immunology* **8**, 239-245, doi:10.1038/ni1443 (2007).
23 B-M., S. Vol. Version 14 (Lawrenceville, NJ, 2015).
24 Velu, V. *et al.* Enhancing SIV-specific immunity in vivo by PD-1 blockade. *Nature* **458**, 206-210, doi:10.1038/nature07662 (2009).
25 Wang, C. *et al.* In vitro characterization of the anti-PD-1 antibody nivolumab, BMS-936558, and in vivo toxicology in non-human primates. *Cancer immunology research* **2**, 846-856, doi:10.1158/2326-6066.cir-14-0040 (2014).
26 Spranger, S. *et al.* Up-regulation of PD-L1, IDO, and T(regs) in the melanoma tumor microenvironment is driven by CD8(+) T cells. *Science translational medicine* **5**, 200ra116, doi:10.1126/scitranslmed.3006504 (2013).
27 Chapman, J. *et al.* Complicated diverticulitis: is it time to rethink the rules? *Annals of surgery* **242**, 576-581; discussion 581-573 (2005).
28 Hart, A. R., Kennedy, H. J., Stebbings, W. S. & Day, N. E. How frequently do large bowel diverticula perforate? An incidence and cross-sectional study. *European journal of gastroenterology & hepatology* **12**, 661-665 (2000).

29 Morris, C. R. *et al.* Anti-inflammatory drugs, analgesics and the risk of perforated colonic diverticular disease. *The British journal of surgery* **90**, 1267-1272, doi:10.1002/bjs.4221 (2003).

20.0 APPENDICES

Appendix 1. US Prescribing Information.

Appendix 2. EU Summary of Product Characteristics.

Appendix 3. Management Algorithms for Adverse Effects.

Appendix 4. Translational science
