# Supplementary material for: Nivolumab for mismatch-repair-deficient or hypermutated gynecologic cancers: a phase 2 trial with biomarker analyses
Source: Nat Med. 2024 Apr 23;30(5):1330–8. doi: 10.1038/s41591-024-02942-7 (PMC11108776; doi:10.1038/s41591-024-02942-7)
Supplement: Supplementary file 2 — Reporting Summary [file 41591_2024_2942_MOESM2_ESM.pdf]

nature portfolio

Corresponding author(s): Clarie Friedman
Dmitriy Zamarin

Last updated by author(s): Feb 19, 2024

Reporting Summary

Nature Portfolio wishes to improve the reproducibility of the work that we publish. This form provides structure for consistency and transparency in reporting. For further information on Nature Portfolio policies, see our Editorial Policies and the Editorial Policy Checklist.

Statistics

For all statistical analyses, confirm that the following items are present in the figure legend, table legend, main text, or Methods section.

n/a	Confirmed	
☐	☒	The exact sample size (*n*) for each experimental group/condition, given as a discrete number and unit of measurement
☒	☐	A statement on whether measurements were taken from distinct samples or whether the same sample was measured repeatedly
☐	☒	The statistical test(s) used AND whether they are one- or two-sided<br>*Only common tests should be described solely by name; describe more complex techniques in the Methods section.*
☐	☒	A description of all covariates tested
☐	☒	A description of any assumptions or corrections, such as tests of normality and adjustment for multiple comparisons
☐	☒	A full description of the statistical parameters including central tendency (e.g. means) or other basic estimates (e.g. regression coefficient) AND variation (e.g. standard deviation) or associated estimates of uncertainty (e.g. confidence intervals)
☐	☒	For null hypothesis testing, the test statistic (e.g. *F*, *t*, *r*) with confidence intervals, effect sizes, degrees of freedom and *P* value noted<br>*Give P values as exact values whenever suitable.*
☒	☐	For Bayesian analysis, information on the choice of priors and Markov chain Monte Carlo settings
☒	☐	For hierarchical and complex designs, identification of the appropriate level for tests and full reporting of outcomes
☐	☒	Estimates of effect sizes (e.g. Cohen's *d*, Pearson's *r*), indicating how they were calculated

Our web collection on statistics for biologists contains articles on many of the points above.

Software and code

Policy information about availability of computer code

Data collection	No code was used
Data analysis	Analyses were performed using GraphPad Prism 9.5 and R 4.2.3

For manuscripts utilizing custom algorithms or software that are central to the research but not yet described in published literature, software must be made available to editors and reviewers. We strongly encourage code deposition in a community repository (e.g. GitHub). See the Nature Portfolio guidelines for submitting code & software for further information.

Data

Policy information about availability of data

All manuscripts must include a data availability statement. This statement should provide the following information, where applicable:

- Accession codes, unique identifiers, or web links for publicly available datasets
- A description of any restrictions on data availability
- For clinical datasets or third party data, please ensure that the statement adheres to our policy

MSK IMPACT data will be released on cBioPortal upon publication. mplF images will be available from the BioImage Archive. Whole exome sequencing data will be deposited in dbGAP.

Research involving human participants, their data, or biological material

Policy information about studies with human participants or human data. See also policy information about sex, gender (identity/presentation), and sexual orientation and race, ethnicity and racism.

Reporting on sex and gender	All patients enrolled on this study are female (based on the fact we enrolled only patients with advanced or recurrent gynecologic cancers).
Reporting on race, ethnicity, or other socially relevant groupings	Race and ethnicities are documented as reported by the patient. These are reported in Table 1 as part of the demographic results, but no additional analyses were performed based on self-reported race or ethnicity.
Population characteristics	Eligible patients had recurrent endometrial cancer or a carcinosarcoma, endometrioid or clear cell carcinoma that appeared to have originated in the ovary/fallopian tube or peritoneum, and met one of the following criteria: dMMR, as determined by loss of expression assessed by immunohistochemistry of one or more of the MMR proteins (MSH2, MSH6, MLH1, and PMS2); 2) MSI-H, as determined by next-generation sequencing (NGS) using Memorial Sloan Kettering Cancer Center-Integrated Mutation Profiling of Actionable Cancer Targets (MSK-IMPACT; MSIsensor); or 3) hypermutated tumors, defined as 20 or more non-synonymous somatic mutations on MSK-IMPACT. The median age was 64 years (range, 36-87 years), and 77% of patients were White, 11% were Black, and 6% were Asian. Most patients (83%) had endometrioid endometrial cancer. All patients had a good performance status, with an ECOG of 0 or 1.
Recruitment	Patients were recruited from the Gynecologic Medical Oncology clinics at Memorial Sloan Kettering Cancer Center (MSK). The study was offered to patietns who were judged to be potentially eligible by their treating physicians. MSK is a tertiary referral center, and thus the patient population may not be reflective of the general gynecologic cancer population. In order to participate in the clinical trial, patients had to have a good performance status and organ function; thus, they are likely to be healthier than the general cancer population.
Ethics oversight	This study was reviewed and approved by the Institutional Review Board at Memorial Sloan Kettering Cancer Center.

Note that full information on the approval of the study protocol must also be provided in the manuscript.

Field-specific reporting

Please select the one below that is the best fit for your research. If you are not sure, read the appropriate sections before making your selection.

☒ Life sciences ☐ Behavioural & social sciences ☐ Ecological, evolutionary & environmental sciences

For a reference copy of the document with all sections, see nature.com/documents/nr-reporting-summary-flat.pdf

Life sciences study design

All studies must disclose on these points even when the disclosure is negative.

Sample size	The co-primary objectives were to define 1) PFS24, and 2) the proportion of patients who achieved objective tumor response (ORR) by RECIST v1.1. The sample size calculation for this study was based on a non-promising ORR of 5% and a promising ORR of 25%. To that end, we used a Simon two-stage minimax design. In the first stage, we enrolled 23 eligible patients, and at least 2 patients were required to achieve a response to proceed to stage II. In stage II, an additional 17 patients were enrolled. Among the total 40 patients, if 6 or more patients achieved a response, this treatment regimen would be declared promising. This decision rule had a type I error rate of 0.025 and a type II error rate of 0.05.
Data exclusions	No data were excluded.
Replication	Due to limited human samples, no replication was performed.
Randomization	This was a single arm, phase 2 study. There was only one study group, all of whom received single agent nivolumab. Thus, randomization is not applicable to this study.
Blinding	Blinding was not performed as not relevant in a single arm phase 2 study.

Reporting for specific materials, systems and methods

We require information from authors about some types of materials, experimental systems and methods used in many studies. Here, indicate whether each material, system or method listed is relevant to your study. If you are not sure if a list item applies to your research, read the appropriate section before selecting a response.

Materials & experimental systems

n/a	Involved in the study
☐	☒ Antibodies
☒	☐ Eukaryotic cell lines
☒	☐ Palaeontology and archaeology
☒	☐ Animals and other organisms
☐	☒ Clinical data
☒	☐ Dual use research of concern
☒	☐ Plants

Methods

n/a	Involved in the study
☒	☐ ChIP-seq
☒	☐ Flow cytometry
☒	☐ MRI-based neuroimaging

Antibodies

Antibodies used

The antibody panel included FOXP3 (236A/E7, Biocare), programmed death ligand-1 (PD-L1, 1:400, E1L3N, Cell Signaling), CD8 (4B11, 1:500, Leica), PAX8 (EPR18715, 1:1000, Abcam), PD-1 (EPR4877(2), 1:400, Abcam), TOX (E6I3Q, 1:7000, Cell Signaling), as well as 4′,6-diamidino-2-phenylindole (DAPI).

Validation

Please see attached document.

Clinical data

Policy information about clinical studies

All manuscripts should comply with the ICMJE guidelines for publication of clinical research and a completed CONSORT checklist must be included with all submissions.

Clinical trial registration

NCT03241745

Study protocol

The study protocol is provided as a supplementary document with the manuscript submission.

Data collection

This was a single-center, investigator-initiated, single-arm, phase II study conducted at MSK. The study opened to accrual on August 3, 2017. The first patient consented on September 27, 2017, and the final patient on May 24, 2021. The trial has completed as of July 1, 2022.

Outcomes

The co-primary objectives were to define 1) PFS24, and 2) the proportion of patients who achieved objective tumor response (ORR) by RECIST v1.1.1 Secondary objectives included PFS, OS, safety and toxicity, DOR, and DCR. Exploratory objectives were to 1) Correlate the somatic mutational burden with ORR and PFS24; 2) Correlate the somatic mutational burden with MSIsensor score; 3) Correlate MSIsensor score with MMR immunohistochemistry status; 3) Correlate the pre-treatment immune phenotype with ORR and PFS24. Patients were evaluable for efficacy if they had received at least 1 dose of therapy and had at least 1 post-baseline efficacy assessment. Patients who were evaluable for response and were lost to follow-up or died before the 24-week PFS assessment were considered events. PFS was calculated from start of treatment to progression/recurrence or death or last follow-up, whichever occurred first. OS was calculated from start of treatment to death or last follow-up, whichever occurred first. DOR was calculated from time of response (for complete response or partial response) to progression, death, or last follow-up. OS, PFS, and DOR rates were estimated using the Kaplan-Meier method. Adverse events were tabulated. Correlation of response with translational parameters was performed by dichotomizing patients based on PFS24, and distribution of the continuous biomarkers (e.g., percentages of CD8+PD1+ cells) between the 2 groups was compared using Mann-Whitney test. TMBs were compared using the Mann-Whitney test, and comparisons of frequency of mutations were performed using two-tailed Fisher exact tests. For exploratory translational analyses no adjustments for multiple comparisons were performed.

Plants

Seed stocks

Report on the source of all seed stocks or other plant material used. If applicable, state the seed stock centre and catalogue number. If plant specimens were collected from the field, describe the collection location, date and sampling procedures.

Novel plant genotypes

Describe the methods by which all novel plant genotypes were produced. This includes those generated by transgenic approaches, gene editing, chemical/radiation-based mutagenesis and hybridization. For transgenic lines, describe the transformation method, the number of independent lines analyzed and the generation upon which experiments were performed. For gene-edited lines, describe the editor used, the endogenous sequence targeted for editing, the targeting guide RNA sequence (if applicable) and how the editor was applied.

Authentication

Describe any authentication procedures for each seed stock used or novel genotype generated. Describe any experiments used to assess the effect of a mutation and, where applicable, how potential secondary effects (e.g. second site T-DNA insertions, mosiacism, off-target gene editing) were examined.
