# Supplementary material for: Nivolumab for mismatch-repair-deficient or hypermutated gynecologic cancers: a phase 2 trial with biomarker analyses
Source: Nat Med. 2024 Apr 23;30(5):1330–8. doi: 10.1038/s41591-024-02942-7 (PMC11108776; doi:10.1038/s41591-024-02942-7)
Supplement: Supplementary file 3 — Antibody validations. [file 41591_2024_2942_MOESM3_ESM.pdf]

**ANTIBODY VALIDATIONS:**

1. FOXP3 (236A/E7, 1:100 Biocare).

**Product name:**
Anti-FOXP3 antibody [236A/E7]
**Description:**
Mouse monoclonal [236A/E7] to FOXP3
**Specificity:**
The epitope recognized by FOXP3 antibody [236A/E7] (ab20034) is between amino acids 105-236. FOXP3 antibody [236A/E7] (ab20034) is expected to detect full length FOXP3 as well as both cleaved forms.
**Tested applications**
Suitable for: mIHC, IHC-P, WB
**Species reactivity**
Reacts with: Human
**Immunogen**
Recombinant full length protein corresponding to Human FOXP3.
**Positive control**
IHC-P: Human tonsil and thymus tissue. mIHC: Human breast cancer tissue.
**Reference (over 500)**
Roncador G, et al. FOXP3, a selective marker for a subset of adult T-cell leukaemia/lymphoma. Leukemia. 2005 Dec; 19(12):2247-53.

2. Programmed death ligand-1 (PD-L1, 1:400, E1L3N, Cell Signaling)

**Product name:**
Anti-PD-L1 antibody [E1L3N]
**Description:**
Rabbit IgG
**Specificity:**
Human PD-L1
**Tested applications**
Suitable for: mIHC, IHC-P, WB
**Species reactivity**
Reacts with: Human
**Immunogen**
Monoclonal antibody is produced by immunizing animals with a synthetic peptide corresponding to residues near the carboxy terminus of human PD-L1 protein.
**Positive control**
IHC: human lung cancer, human placenta, cell pellets expressing PD-L1
**References (over 800)**
Akhilesh Mishra et al., Non-invasive PD-L1 quantification using [18F]DK222-PET imaging in cancer immunotherapy. J Immunother Cancer. 2023 Oct;11(10):e007535.

3. CD8 (4B11, 1:500, Leica)

**Product name:**
Anti-CD8 antibody
**Description:**
Mouse IgG
**Specificity:**
Human CD8
**Tested applications**

Suitable for: IHC
**Species reactivity**
Reacts with: Human
**Immunogen**
Synthetic peptide derived from the carboxy terminal region of the human CD8 alpha chain coupled to a N-terminal cysteine, with the sequence C-KSDGKPSLSARYV (amino acid range 223-235).
**Positive control**
IHC: Human lymph node, T cell lymphoma, human tonsil
**References**
O'Connor MH et al. A follicular regulatory Innate Lymphoid Cell population impairs interactions between germinal center Tfh and B cells. " Communications biology, 2021.

4. PAX8 (EPR18715, 1:1000, Abcam)

**Product name:**
Anti-PAX8 antibody
**Description:**
Rabit monoclonal
**Specificity:**
Human PAX8
**Tested applications**
Suitable for: IHC
**Species reactivity**
Reacts with: Human
**Immunogen**
Recombinant fragment. This information is proprietary to Abcam and/or its suppliers.
**Positive control**
IHC: Human thyroid carcinoma and endometrium carcinoma tissues.
**References**
Li M et al. Novel Missense Variants in PAX8 and NKX2-1 Cause Congenital Hypothyroidism. Int J Mol Sci 24:N/A (2023).

5. PD-1 (EPR4877(2), 1:400, Abcam)

**Product name:**
Anti-PD-1 antibody
**Description:**
Rabit monoclonal
**Specificity:**
Human PD-1
**Tested applications**
Suitable for: IHC
**Species reactivity**
Reacts with: Human
**Immunogen**
Synthetic peptide within Human PD1 aa 1-100. The exact sequence is proprietary.
**Positive control**
IHC: Human tonsil tissue.
**References**

Nakamura M  et al. Tertiary Lymphoid Structures and Chemokine Landscape in Virus-Positive and Virus-Negative Merkel Cell Carcinoma. Front Oncol 12:811586 (2022).

6. TOX (E6I3Q, 1:7000, Cell Signaling)

**Product name:**
Anti-TOX antibody
**Description:**
Rabit monoclonal
**Specificity:**
Human, rat, and mouse TOX
**Tested applications**
Suitable for: IHC
**Species reactivity**
Reacts with: Human, rat, mouse
**Immunogen**
Monoclonal antibody is produced by immunizing animals with a synthetic peptide corresponding to residues surrounding Ala522 of human Tox protein.
**Positive control**
IHC: Human colon cancer, human renal cancer, human gastric cancer, human spleen
**References**
Cheng et al., Identification of Differentially Expressed Genes and Prediction of Expression Regulation Networks in Dysfunctional Endothelium. Genes (Basel). 2022 Aug 30;13(9):1563.
